# Mesozooplankton taurine production and prokaryotic uptake in the northern Adriatic Sea

**DOI:** 10.1002/lno.11544

**Published:** 2020-06-25

**Authors:** Elisabeth L. Clifford, Daniele De Corte, Chie Amano, Paolo Paliaga, Ingrid Ivančić, Victor Ortiz, Mirjana Najdek, Gerhard J. Herndl, Eva Sintes

**Affiliations:** ^1^ Department of Functional and Evolutionary Ecology University of Vienna Vienna Austria; ^2^ Research and Development Center for Marine Biosciences Japan Agency for Marine‐Earth Science and Technology (JAMSTEC) Yokosuka Japan; ^3^ Department of Natural and Health Sciences Juraj Dobrila University of Pula Pula Croatia; ^4^ Center for Marine Research Ruđer Bošković Institute Rovinj Croatia; ^5^ Royal Netherlands Institute for Sea Research (NIOZ), Department of Marine Microbiology and Biogeochemistry Utrecht University Den Burg The Netherlands; ^6^ Instituto Español de Oceanografía (IEO) Centro Oceanográfico de Baleares Palma de Mallorca Spain

## Abstract

Dissolved free taurine, an important osmolyte in phytoplankton and metazoans, has been shown to be a significant carbon and energy source for prokaryotes in the North Atlantic throughout the water column. However, the extent of the coupling between taurine production and consumption over a seasonal cycle has not been examined yet. We determined taurine production by abundant crustacean zooplankton and its role as a carbon and energy source for several prokaryotic taxa in the northern Adriatic Sea over a seasonal cycle. Taurine concentrations were generally in the low nanomolar range, reaching a maximum of 22 nmol L^−1^ in fall during a *Pseudonitzschia* bloom and coinciding with the highest zooplankton taurine release rates. Taurine accounted for up to 5% of the carbon, 11% of the nitrogen, and up to 71% of the sulfur requirements of heterotrophic prokaryotes. Members of the *Roseobacter* clade, *Alteromonas*, Thaumarchaeota, and Euryarchaeota exhibited higher cell‐specific taurine assimilation rates than SAR11 cells. However, cell‐specific taurine and leucine assimilation were highly variable in all taxa, suggesting species and/or ecotype specific utilization patterns of taurine and dissolved free amino acids. Copepods were able to cover the bulk taurine requirements of the prokaryotic communities in fall and winter and partly in the spring–summer period. Overall, our study emphasizes the significance of taurine as a carbon and energy source for the prokaryotic community in the northern Adriatic Sea and the importance of crustacean zooplankton as a significant source of taurine and other organic compounds for the heterotrophic prokaryotic community.

Zooplankton and various phytoplankton species are significant sources of taurine and other bioreactive compounds in the marine environment (Clifford et al. 2017; Landa et al. 2019). Similar to dimethylsulfoniopropionate (DMSP) and glycine betaine, taurine serves as an important osmolyte in algae and metazoans (Yancey 2005; Tevatia et al. 2015). Taurine can either be respired by prokaryotes through the Krebs cycle and therefore, used as an energy source, or be incorporated into their biomass (Cook and Denger 2006), contributing 16% ± 16% to 21% ± 15% to the heterotrophic bulk C‐biomass production in open ocean bacterioplankton communities throughout the water column (Clifford et al. 2019). Taurine ATP‐binding cassette transporters and enzymes involved in taurine degradation are widespread in marine prokaryotic communities both in the open ocean and in coastal waters (Hanson et al. 2014; Williams and Cavicchioli 2014). Taurine concentrations in coastal waters vary in the nanomolar range (up to ∼ 30 nmol L^−1^) (Lu et al. 2014) and are slightly higher than in open ocean surface waters, with concentrations ranging between 0.1 and 15.7 nmol L^−1^ (Clifford et al. 2017). However, the role of taurine for coastal prokaryotic communities has not been assessed yet.

Members of the abundant *Rhodobacteraceae* and SAR11 clade play a prominent role in utilizing biologically labile organic compounds, such as taurine (Steindler et al. 2011; Lenk et al. 2012), amino acids, and DMSP (Malmstrom et al. 2004*a*). SAR11 is also responsible for a large fraction of taurine assimilation in the open ocean, especially in epipelagic waters (Clifford et al. 2019). *Pelagibacter ubique* is able to grow on taurine‐C alone in natural seawater media (Steindler et al. 2011). Additionally, *Rhodobacteraceae* have been proposed to utilize taurine as a C‐ and energy source in freshwater and marine environments (Simon et al. 2017). Members of the *Roseobacter* clade (Lenk et al. 2012) and *Sulfitobacter* (Amin et al. 2015) utilize taurine as a C‐source; additionally, the latter can also utilize the breakdown product sulfite as an energy source (Park et al. 2007). Also the archaeal groups Euryarchaeota and Thaumarchaeota are capable of using taurine (Sauder et al. 2017; Clifford et al. 2019).

Dissolved organic matter (DOM) released by crustacean zooplankton and phytoplankton can generate shifts in the prokaryotic community composition on a time scale of a few hours to days (Valdés et al. 2017; Choi et al. 2018). Members of SAR11 and *Rhodobacteraceae* are often present in high abundances during bloom events (Alonso and Pernthaler 2006; Choi et al. 2018), utilizing taurine and other low‐molecular weight organic compounds (Li et al. 2018). Toward the decline of a bloom, however, other prokaryotic groups dominate the community, such as Bacteroidetes, degrading high‐molecular weight organic compounds (Alonso and Pernthaler 2006; Choi et al. 2018).

The northern Adriatic Sea is a highly dynamic system, influenced by freshwater input, especially from the Po River, and by the northwards flow of waters from the oligotrophic central Adriatic (Kraus et al. 2016). To elucidate the link between taurine release by crustacean zooplankton and uptake by prominent prokaryotic taxa, we investigated taurine production and utilization dynamics over a seasonal cycle in the northern Adriatic Sea.

## *Materials and methods*

### Sampling location

Sampling was conducted ∼ 1 km off the coast of Rovinj, Croatia (45.08347°N, 13.60518°E) over a seasonal cycle: in April 2015 (late spring), June 2015 (early summer), November 2015 (fall), and February 2016 (winter) using the R/V *Burin*. Each sampling campaign consisted of 7–10 sampling days. Seawater was collected with 5‐liter Niskin bottles from three different depth layers (5, 10, and 15 m). Water from the Niskin bottles was dispensed into prerinsed polycarbonate flasks and further processed as described below. Zooplankton samples were collected using vertical plankton tows (200 *μ*m mesh size; hoisted at 15 m min^−1^) from 15 to 30 m depth to the surface. The samples were transferred to the laboratory of the Center of Marine Research (Ruder Bošković Institute) at Rovinj within 30 min and immediately processed as described below. Sea surface temperature was obtained from https://oceandata.sci.gsfc.nasa.gov/MODIS-Aqua (NASA Goddard Space Flight Center et al. 2018). Discrete temperature measurements from water samples were obtained with a thermometer on board.

### Inorganic nutrients

Seawater samples were filtered through precombusted Whatman GF/F filters and stored in polyethylene bottles at −20°C. Analyses of inorganic nutrients (nitrite, nitrate, ammonia, dissolved inorganic nitrogen, silicate, phosphate, dissolved organic phosphate) were conducted following standard protocols (Strickland and Parson 1972).

### Dissolved free taurine and dissolved free amino acid concentrations

Five milliliters seawater samples were taken from the 100 mL polycarbonate flasks with 20 mL syringes and gently filtered through prerinsed 0.2 *μ*m pore‐size Acrodisc filters (25 mm; Pall, Supor membrane) into precombusted (at 450°C for 4 h) glass vials. Afterward, the filtered seawater samples were stored at −20°C until further analyses using high‐pressure liquid chromatography (HPLC) and fluorescence detection after precolumn *ortho*‐phthaldialdehyde derivatization (Clifford et al. 2017). Concentrations of taurine and 19 dissolved free amino acid (DFAA) species were measured. The limit of detection (LOD) and limit of quantification (LOQ) for taurine were 0.01 nmol L^−1^ and 0.05 nmol L^−1^, respectively. The LOD of DFAAs ranged between 0.01 and 0.06 nmol L^−1^, while the LOQ of DFAAs varied between 0.03 and 0.2 nmol L^−1^. Detailed information on LOD, LOQ, the linearity (*R* ^2^), and the recovery for taurine and the different DFAA species are reported elsewhere (Clifford et al. 2017, 2019). Valine and methionine were coeluting; hence, their concentrations are given as the sum of Val/Met. Considering the high precision and accuracy of the method, samples were measured only once, hence without replicates. However, randomly selected duplicate samples were analyzed in each HPLC run. Measurements between duplicates varied by 0.99% and 2.57% for taurine and leucine, respectively, using percentile spread precision calculation (Hyslop and White 2009). Bias estimates ranged between 1.85% and 0.38% for taurine and leucine, respectively (Hyslop and White 2009). Percentile spread calculation was used to limit the effect of outliers on the precision estimation (Hyslop and White 2009). Within the 43 duplicates measured, three outliers were detected for both taurine and leucine (*Z*‐score > 1.5).

### Dissolved free taurine and DFAA release by crustacean mesozooplankton

The crustacean mesozooplankton were concentrated over a 63 *μ*m Nitex screen avoiding any contact of the specimens to air and subsequently transferred into 0.2 *μ*m filtered seawater from the same location. Between 44 and 125 specimens of crustacean mesozooplankton (Supporting Information Tables S1, S2) were sorted using a dissecting microscope and transferred into 500 mL precombusted glass jars filled with 0.2 *μ*m filtered seawater from the same location. Subsequently, the crustacean zooplankton were incubated under dim light at in situ temperature using a running seawater system.

Mixed crustacean mesozooplankton incubations were performed in triplicate, with the exception of the first experiment in April (done in duplicate, Supporting Information Tables S1, S2). Seawater samples were taken prior to the addition of the zooplankton and at the beginning of the incubation (immediately after adding the zooplankton) to determine taurine and DFAA concentrations and prokaryotic abundance. Afterward, samples were collected at 0.5 h intervals for 2 h and at 3, 5, and 8 h after starting the incubation. Additionally, a control consisting of 0.2 *μ*m filtered seawater without zooplankton added was sampled at the same time intervals as the zooplankton incubations. Subsamples of dissolved free taurine and DFAA concentrations (4 mL) as well as bacterial abundance (1.5 mL) were taken at each time point and stored at −20°C until further analyses. Release rates were calculated using linear regression analyses until the time point when the first DFAA species or taurine did not further increase in concentration (Verity 1985; Clifford et al. 2017) (Supporting Information Tables S3, S4). Regressions with *R* ^2^ < 0.60 and *p* value > 0.05 were excluded from calculating release rates (Supporting Information Tables S3, S4).

After incubation, the zooplankton specimens were fixed with formaldehyde (4% final concentration) and stored at 4°C. The length and diameter of the zooplankton specimens (Supporting Information Table S1) were determined under a dissecting microscope with a calibrated Stereo Lumar V.12 (ZEN software) to estimate their biovolume using the formula of an ellipsoid (*V* = $\frac{4}{3}\mathrm{\pi a}b^{2}$) (Lawrence et al. 1987) where *a* represents the prosome length/2 and *b* the prosome diameter/2. The biovolume was converted into C‐biomass using a factor of 0.08 pg C *μ*m^−3^ (Beers and Stewart 1970).

The release rates of taurine and leucine by the crustacean zooplankton community for each season were multiplied by the copepod abundance obtained from the literature (Kamburska and Fonda‐Umani 2006) (Supporting Information Table S5) to estimate in situ bulk release rates. Turnover rates of taurine and leucine were estimated by dividing the in situ mean bulk release rates by the mean seasonal dissolved free taurine and leucine concentrations, respectively. A detailed description of these calculations is given elsewhere (Clifford et al. 2017).

Taurine release experiments with *Mytilus* sp. were also performed. However, the release rates could not be reliably estimated due to the large variability in the taurine concentrations over time in the incubations, probably associated with the high variability in filtration rates of these organisms. It is noteworthy, however, that *Mytilus* sp. released copious amounts of taurine right at the start of the incubations (data not shown).

### Prokaryotic and picophytoplankton abundance

To determine microbial abundance, seawater samples (1.5 mL) were fixed with glutaraldehyde (0.5% final concentration) at 4°C for 10 min and stored at −80°C after flash‐freezing in liquid nitrogen. At the home lab, the samples were thawed to room temperature and 0.5 mL subsamples were stained with SYBR Green I (1x final concentration) in the dark for 10 min. One micrometer fluorescent beads (Molecular Probes, 1 × 10^5^ mL^−1^ final concentration) were added to the samples as an internal standard. Total prokaryotes (including cyanobacteria) were enumerated by flow cytometry (Accuri C6, Becton Dickinson) based on their signature in a plot of green fluorescence vs. side scatter (Brussaard 2004). *Prochlorococcus*, *Synechococcus*, and photoautotrophic picoeukaryotes were also enumerated by flow cytometry based on their distinct autofluorescence signals. However, phytoplankton might be underestimated by the Accuri flow cytometer (Gérikas Ribeiro et al. 2016).

Primary production data were obtained from the Ocean Productivity website (http://www.science.oregonstate.edu/ocean.productivity/). These primary production estimates are based on remote sensing of surface waters (Behrenfeld and Falkowski 1997). Primary production rates are shown as the mean values corresponding to the sampling week. Figures were generated using Ocean Data View (ODV, version 4, https://odv.awi.de) (Schlitzer 2018).

### Microbial taurine assimilation and respiration measurements

Taurine assimilation and respiration rates were assessed as previously described (Clifford et al. 2019). Briefly, duplicate seawater samples (10 mL) and one formaldehyde‐killed blank were spiked with ^14^C‐taurine (Biotrend, specific activity: 60 mCi/mmol; final concentration: 40 nmol L^−1^) and incubated in ∼ 120 mL Biological Oxygen Demand flasks sealed with a rubber stopper at in situ temperature in the dark for 2–6 h (Hobbie and Crawford 1969) depending on the expected activity. The 40 nmol L^−1^ taurine concentration added to the samples represented the saturating substrate concentration (Supporting Information Fig. S1). Incubations were terminated by adding 0.8 mL sulfuric acid (2 mol L^–1^ H_2_SO_4_) injected with a syringe through the rubber stopper to acidify the sample. The ^14^CO_2_ originating from the respired ^14^C‐taurine was trapped in a filter‐paper wick (Whatman nº1) placed in a plastic cup in the headspace of the flasks after adding 200 *μ*L of phenethylamine. Subsequently, the samples were shaken at room temperature to facilitate ^14^CO_2_ trapping for 1–2 h. The fixed seawater samples were filtered onto 0.2 *μ*m polycarbonate filters (Millipore GTTP, 25 mm diameter). The filters and the filter paper wicks were placed into scintillation vials and after adding Filter Count scintillation cocktail (Perkin Elmer), the disintegrations per minute (DPMs) were determined in the scintillation counter (Canberra Packard Tricarb 2900 TR, Perkin Elmer Packard, U.S.A.). The DPMs of the two replicates from each sample were averaged and DPMs from the killed control subtracted.

The DPMs measured from the filter were used to determine taurine assimilation and the DPMs obtained from the paper wick to estimate respiration rates. The sum of taurine assimilation and respiration represents the total taurine uptake. Measured taurine concentrations were used to correct the taurine assimilation and respiration rates for the external isotope dilution by multiplying the rates by (Tau_meas_ + Tau_add_)/Tau_add_, where Tau_meas_ is the measured taurine concentration in nmol L^−1^ in the corresponding seawater sample and Tau_add_ the nmol L^−1^ taurine added (i.e., 40 nmol L^−1^). Taurine uptake rates (assimilation plus respiration) were divided by the environmental taurine concentrations to obtain the taurine turnover rates. Taurine assimilation efficiency as a percentage of total uptake was estimated from the taurine assimilation rate divided by the taurine uptake rate and multiplied by 100.

### Leucine incorporation measurements

The centrifugation method was used to measure the ^3^H‐leucine incorporation into prokaryotic protein as described elsewhere (Kirchman 2001). Four replicates of 1.2 mL seawater and two trichloroacetic acid (TCA)‐killed controls were inoculated with 40 nmol L^−1 3^H‐leucine (Biotrend, specific activity: 120 Ci/mmol) and incubated at in situ temperature in the dark for 2–6 h, depending on the expected activity. After the incubation, samples were fixed with TCA (final concentration 5%). Fixed samples were centrifuged at 21,000 × *g* at 4°C for 10 min. The resulting pellet was washed with 5% TCA and centrifuged again (21,000 × *g* at 4°C for 10 min). Subsequently, 1 mL of Filter Count scintillation cocktail (Perkin Elmer) was added to the pellet. DPMs were determined in a Canberra Packard Tricarb 2900 TR (Perkin Elmer Packard, U.S.A.) and converted into leucine incorporation rates. The dissolved free leucine concentration of the corresponding seawater samples was used to calculate the external isotope dilution factor for each sample ([Leu_meas_ + Leu_add_]/Leu_add_, where Leu_meas_: nmol L^−1^ leucine measured, Leu_add_: nmol L^−1^ leucine added, i.e., 40 nmol L^−1^). Leucine incorporation rates were divided by the corresponding in situ dissolved free leucine concentrations to calculate the leucine turnover rates.

Leucine incorporation rates were converted to heterotrophic prokaryotic C‐biomass production by applying the theoretical conversion factor of 1.55 kg C mol^−1^ leucine corrected for the external isotope dilution by taking the concentration of the ambient leucine into account (Kirchman 2001).

### Microautoradiography combined with catalyzed reporter deposition fluorescence in situ hybridization

Microautoradiography combined with catalyzed reporter deposition fluorescence in situ hybridization (MICRO‐CARD‐FISH) analyses were performed once per season to determine the abundance of specific prokaryotic taxa taking up taurine and leucine. Briefly, 2–10 mL water samples and one formaldehyde‐killed control (2% final concentration) were spiked with ^3^H‐taurine or ^3^H‐leucine at a final concentration of 40 nmol L^−1^ and subsequently incubated in the dark at in situ temperature for 2–6 h, depending on the expected prokaryotic activity. After the incubation, samples were filtered onto 0.2 *μ*m polycarbonate filters, and processed as described elsewhere (Sintes and Herndl 2006). Information on the oligonucleotide probes, the hybridization conditions, and the target phylogenetic groups examined is given in Supporting Information Table S6. The Eub338‐I coverage of bacteria is > 87% according to our search against Silva database with TestProbe v3.0 (https://www.arb-silva.de/search/testprobe) (Quast et al. 2012). However, Planctomycetes and Verrumicrobia were not efficiently detected with this probe (Daims et al. 1999). Thus, probes Eub338‐II, adding a higher coverage of Planctomycetes taxa (47%), and Eub338‐III, adding additional 20% and 60% coverage to Chloroflexi and Verrumicrobia taxa, respectively, were used in this study in combination with Eub338‐I (Daims et al. 1999). Worden et al. (2000) reported that some specific *Synechococcus* strains showed mismatches to the Eub338‐I probe; however, this probe's coverage of Cyanobacteria is 80.5% according to our search against Silva database (https://www.arb-silva.de/search/testprobe) (Quast et al. 2012). Thus, no significant bias in the detection of major marine taxa is expected using this set of probes. After CARD‐FISH hybridization, the filters were exposed to photographic emulsion (Carestream NTB) at 4°C for 12 d (for taurine) and 2 d (for leucine), when the number of cells with attached silver grains did not further increase as determined in previous experiments (Clifford et al. 2019). Filters were examined for total prokaryotic cells (i.e., 4′,6‐diamidino‐2‐phenylindole‐stained [DAPI]), probe‐positive cells (cells hybridized with the specific fluorescing oligonucleotide probe), and total prokaryotes and probe‐stained cells assimilating the radiolabeled substrate (cells with silver grains attached to the cell). The halo area of the silver grains surrounding cells, indicating the uptake of the respective radiolabeled substrate, was quantified as previously described (Sintes and Herndl 2006).

Microautoradiography cannot distinguish radiolabeled substrate taken up from that incorporated into macromolecules. However, considering that bulk uptake corresponded to the sum of assimilation and respiration rates, and that respired molecules are not likely to remain inside the cells for long, we expect that single cell activity assessed by MICRO‐CARD‐FISH resembles assimilation. Moreover, the CARD‐FISH protocol involves permeabilizing the cells' membranes and several washing steps, favoring leakage of nonincorporated radiolabeled molecules. Hence, the term “assimilation” is used for the MICRO‐CARD‐FISH results.

To determine the proportion of C, N, and/or S requirements of the different prokaryotic groups potentially provided by taurine and leucine uptake, the bulk taurine and leucine assimilation rates of these taxa were calculated by multiplying the taxon's average single cell assimilation rates (nmol cell^−1^ d^−1^) by the number of prokaryotes of that particular taxon assimilating taurine or leucine. The bulk taurine and leucine release rates by copepods were estimated as described above using copepod abundance data from this area obtained from the literature (Kamburska and Fonda‐Umani 2006).

### Contribution of taurine‐C, ‐N, and ‐S to bulk prokaryotic biomass production and single cell activity of specific prokaryotic groups

The contribution of taurine assimilation to the heterotrophic bulk and single cell prokaryotic C‐biomass production was estimated as follows: The taurine assimilation rates were converted to taurine‐derived C assimilated, while heterotrophic biomass production was derived from the leucine incorporation rates as described above.

The potential contributions of taurine‐N and taurine‐S to the N‐ and S‐requirements of the heterotrophic prokaryotic bulk community were calculated applying the atomic ratio of bacterial cells of C : N of 4 : 1 and C : S of 26 : 1 (Fagerbakke et al. 1996). Similarly, the contribution of taurine‐C, taurine‐N, and taurine‐S to the requirements of specific prokaryotic groups was estimated based on quantitative MICRO‐CARD‐FISH (Sintes and Herndl 2006). Estimates of single cell assimilation of taurine were converted to single cell taurine‐derived C, taurine‐derived N, and taurine‐derived S as described above for the bulk rates, whereas single cell biomass production was estimated from the leucine assimilation of the specific prokaryotic taxa.

### Statistical analysis

The Kruskal–Wallis test was used to test significant differences between the depth layers. Kruskal–Wallis test followed by a Mann–Whitney *U*‐test was used to test significant differences between the different seasons as a nonparametric equivalent for a parametric post hoc test. Results were assumed to be significantly different at a *p* value of ≤ 0.05. Statistical analyses were conducted using IBM SPSS Statistics 20.

## *Results*

### Physicochemical characteristics of the water column

**Fig. 1.**
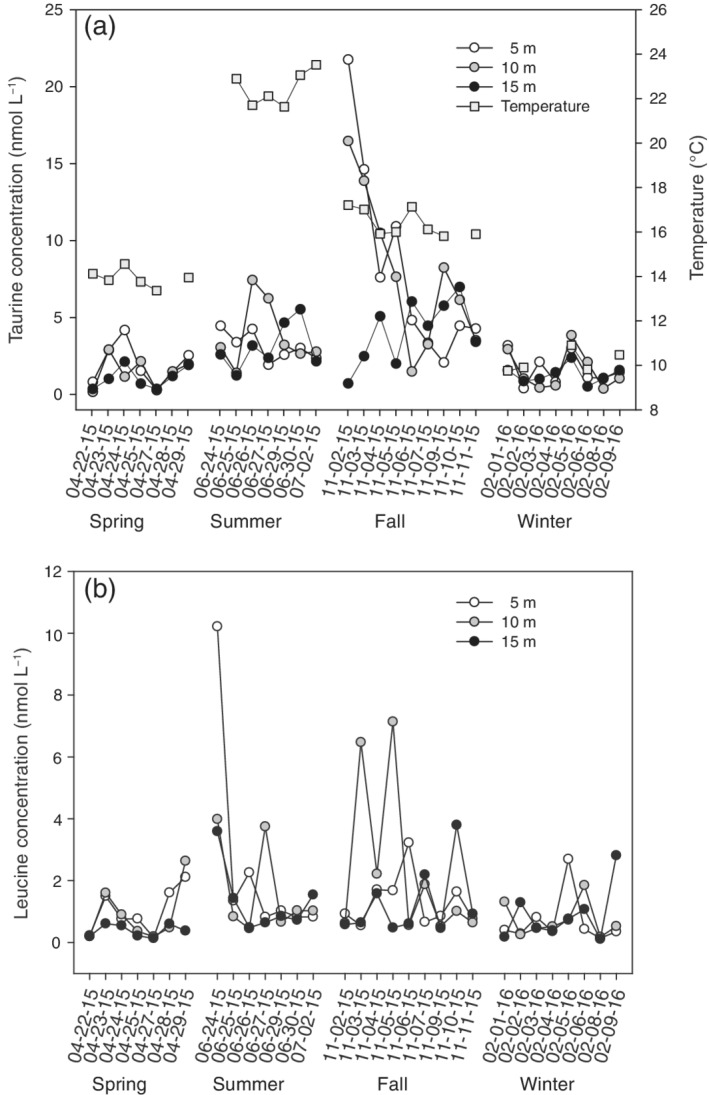
Taurine (**a**) and leucine (**b**) concentrations at 5, 10, and 15 m depth in the northern Adriatic Sea over a seasonal cycle. Surface seawater temperature (°C) is shown in panel (**a**).

Water temperature showed typical seasonal dynamics for the northern Adriatic Sea, with winter temperatures of ∼ 10°C increasing during spring and reaching in the stratified water column in summer 23°C at the sea surface (Fig. 1a).

Nitrate (${\mathrm{NO}}_{3}^{-}$) concentrations were highest in summer (5.2 ± 4.2 *μ*mol L^−1^), whereas nitrite (${\mathrm{NO}}_{2}^{-}$) concentrations peaked in fall (0.3 ± 0.1 *μ*mol L^−1^) and winter (0.4 ± 0.1 *μ*mol L^−1^) (Supporting Information Table S7). Ammonium (${\mathrm{NH}}_{4}^{+}$) did not show a clear pattern over the seasonal cycle ranging between 0.3 ± 0.2 and 0.7 ± 0.8 *μ*mol L^−1^. Phosphate (${\mathrm{PO}}_{4}^{3-}$) and dissolved organic phosphorus (DOP) followed similar trends, with highest concentrations in spring (${\mathrm{PO}}_{4}^{3-}$: 0.06 ± 0.02 *μ*mol L^−1^, DOP: 0.4 ± 0.1 *μ*mol L^−1^; Supporting Information Table S7). Dissolved silicate concentrations decreased from spring (5.2 ± 2.1 *μ*mol L^−1^) to winter (2.8 ± 0.9 *μ*mol L^−1^).

### Microbial abundance and primary production

Total prokaryotic abundance (Supporting Information Fig. S2) increased from spring (2 × 10^5^ cells mL^−1^) toward the fall when it reached 1 × 10^6^ cells mL^−1^, and decreased again toward the winter (3 × 10^5^ cells mL^−1^).

Picoeukaryotic abundance was highest in early fall (up to 7.6 × 10^3^ cells mL^−1^) coinciding with a diatom bloom (Supporting Information Fig. S3). Picoeukaryotic abundance during spring and summer was significantly lower than in fall and winter. *Synechococcus* abundance ranged between 4.5 × 10^3^ and 69.3 × 10^3^ cells mL^−1^ with highest concentrations in the winter (Supporting Information Fig. S4a). *Prochlorococcus* abundance was highest in the fall (up to 7.1 × 10^3^ cells mL^−1^) and substantially lower during the other seasons (Supporting Information Fig. S4b; *p* ≤ 0.05). Primary production estimates obtained from remote sensing peaked during the first sampling week in fall with about 1510 mg C m^−2^ d^−1^ (Supporting Information Fig. S5).

### DFAA and dissolved taurine concentrations

Total DFAA concentrations (Supporting Information Table S8) peaked in fall during the diatom bloom (up to 379 nmol L^−1^). Serine, asparagine, aspartic‐ and glutamic acid, glycine, and alanine were the dominant DFAA species throughout the annual cycle (Supporting Information Table S8). The highest taurine concentrations were also measured during the fall phytoplankton bloom (up to 21.8 nmol L^−1^) (Fig. 1a). Leucine concentrations peaked at the beginning of the summer period (up to 10.2 nmol L^−1^) and during the bloom (up to 7.1 nmol L^−1^). However, daily variations in leucine concentration (Fig. 1b) were more pronounced than its seasonal variation (*p* ≥ 0.05). Taurine/DFAA ratios (Supporting Information Table S8) were highest in summer (0.05 ± 0.03) and fall (0.06 ± 0.04).

### Dissolved free taurine and DFAA release rates by mixed crustacean zooplankton communities dominated by copepods

The spring zooplankton community consisted mainly of the copepod species *Acartia* sp. and *Centropagus* sp., whereas the summer communities were dominated by the Cladocera species *Penilia avirostris* besides *Acartia* sp. and *Centropagus* sp. (Supporting Information Table S1). Fall and winter communities were dominated by copepod species, mainly consisting of the Calanoida *Acartia* sp., *Centropagus* sp., *Calanus* sp., the Cyclopoida *Oithona* sp., *Oncaea* sp. and *Temora stylifera* and the Harpaticoida *Euterpina* sp. and *Microsetella* sp., and other unidentified Calanoida.

**Fig. 2.**
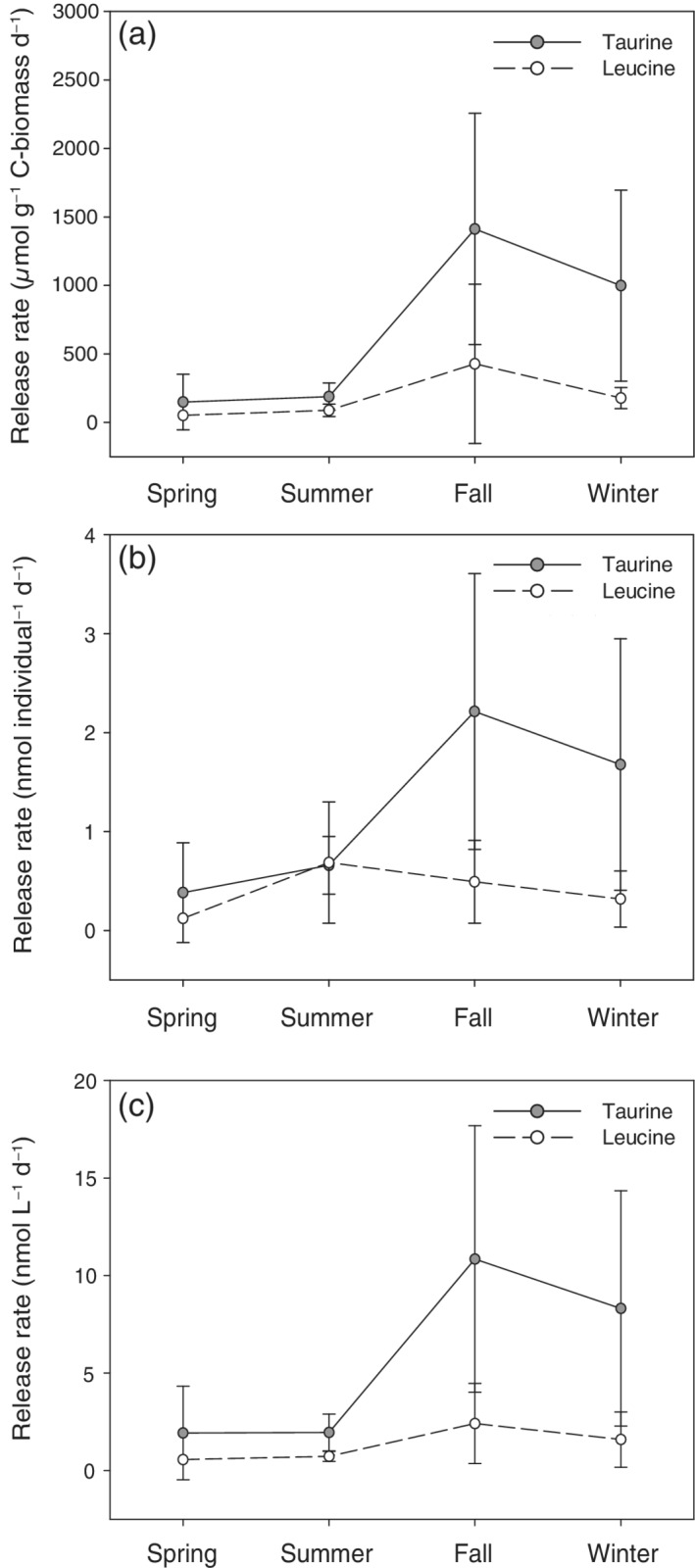
Mean ± standard deviation of taurine‐ and leucine release rates by crustacean zooplankton per g C‐biomass (**a**) and per individual (**b**) in the northern Adriatic Sea over a seasonal cycle. Release rates by the bulk copepod community (average abundances retrieved from Kamburska and Fonda‐Umani 2006; Supporting Information Table S5) of taurine and leucine are shown in panel (**c**).

The main DFAA species released by the zooplankton assemblages throughout the different seasons were glycine, serine, glutamic acid, arginine, and alanine (Supporting Information Tables S3, S4). The DFAA release rates per biomass unit and per individual were highly variable over the different seasons with higher release rates in fall (12280 ± 9125 *μ*mol g^−1^ C‐biomass d^−1^ corresponding to 16 ± 9 nmol individual^−1^ d^−1^) than in spring and summer (spring: 951 ± 1167 *μ*mol g^−1^ C‐biomass d^−1^, 2 ± 3 nmol individual^−1^ d^−1^; summer: 970 ± 875 *μ*mol g^−1^ C‐biomass h^−1^, 3 ± 2 nmol individual^−1^ d^−1^) (*p* ≤ 0.05). The ratio of taurine to total DFAA released ranged, on average, between 0.2 ± 0.1 and 0.3 ± 0.3. Leucine was only occasionally released in spring, summer, and winter. Its contribution to the total DFAA (Supporting Information Table S3) ranged from 0.9% during winter to 10% during fall. Taurine release rates per unit biomass and per specimens followed similar patterns as DFAA. Significantly higher release rates were obtained in fall (1412 ± 844 *μ*mol g^−1^ C‐biomass d^−1^, 2.2 ± 1.4 nmol individual^−1^ d^−1^) than in the other sampling months (*p* ≤ 0.05) (Fig. 2a,b).

Estimated mean bulk taurine release rates (Fig. 2c) based on monthly determined rates and copepod abundance data from this region retrieved from the literature (Supporting Information Table S5) were highest in fall, yielding 10.8 ± 6.8 nmol L^−1^ d^−1^, and in winter with 8.3 ± 6.0 nmol L^−1^ d^−1^. Lowest bulk release rates were estimated for spring (1.9 ± 2.4 nmol L^−1^ d^−1^) and summer (1.9 ± 0.9 nmol L^−1^ d^−1^). Consequently, the estimated taurine turnover rates based on mesozooplankton release rates were highest in winter (on average 5.3 ± 3.8 d^−1^, Supporting Information Table S9) and ranged between 0.6 and 1.6 d^−1^ in the other seasons (spring: 1.2 ± 1.5 d^−1^; summer: 0.6 ± 0.3 d^−1^; fall: 1.6 ± 1.0 d^−1^, on average, Supporting Information Table S9).

### Bulk taurine assimilation and respiration and leucine incorporation

**Fig. 3.**
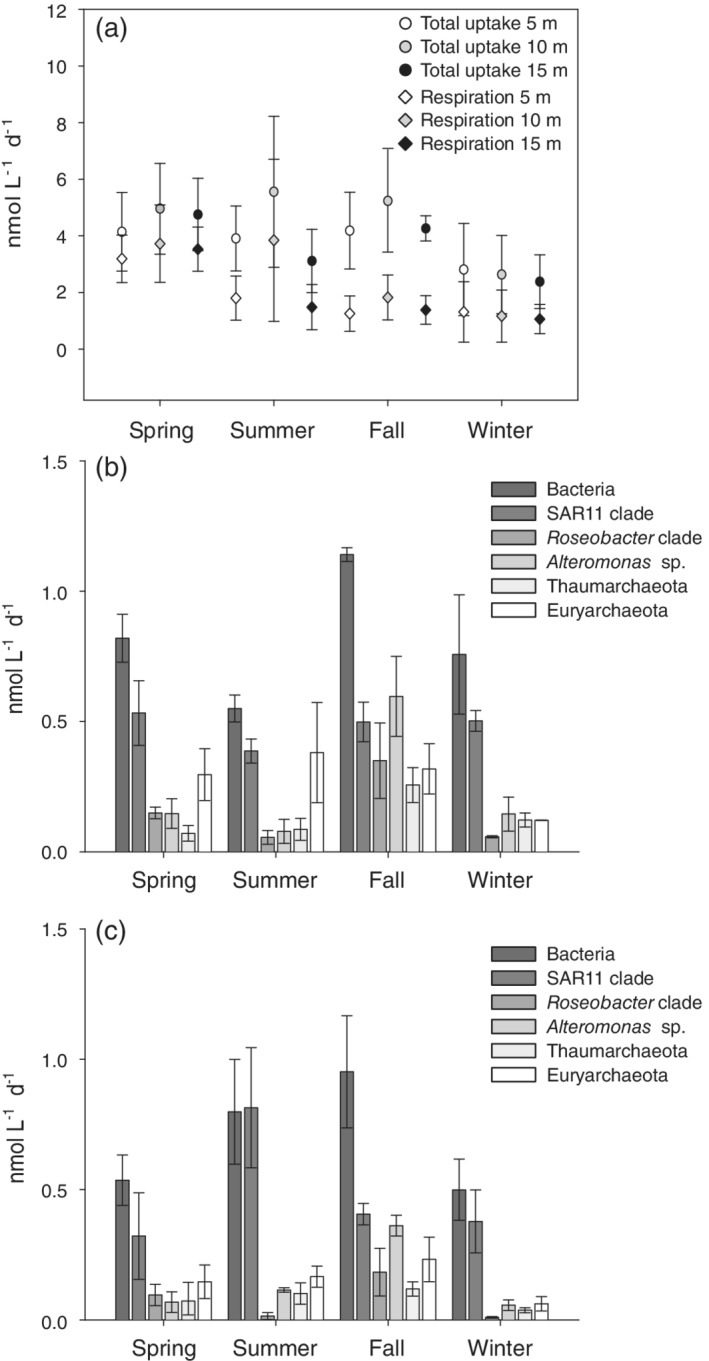
Bulk taurine uptake (respiration + assimilation) and respiration by the prokaryotic community measured with ^14^C‐taurine (**a**). Bulk assimilation rates of taurine (**b**) and leucine (**c**) by the prokaryotic taxa assessed with MICRO‐CARD‐FISH (^3^H‐taurine or ^3^H‐leucine) in the northern Adriatic Sea. Mean ± standard deviation of all three depth layers are shown in panels (**b**) and (**c**).

**Fig. 4.**
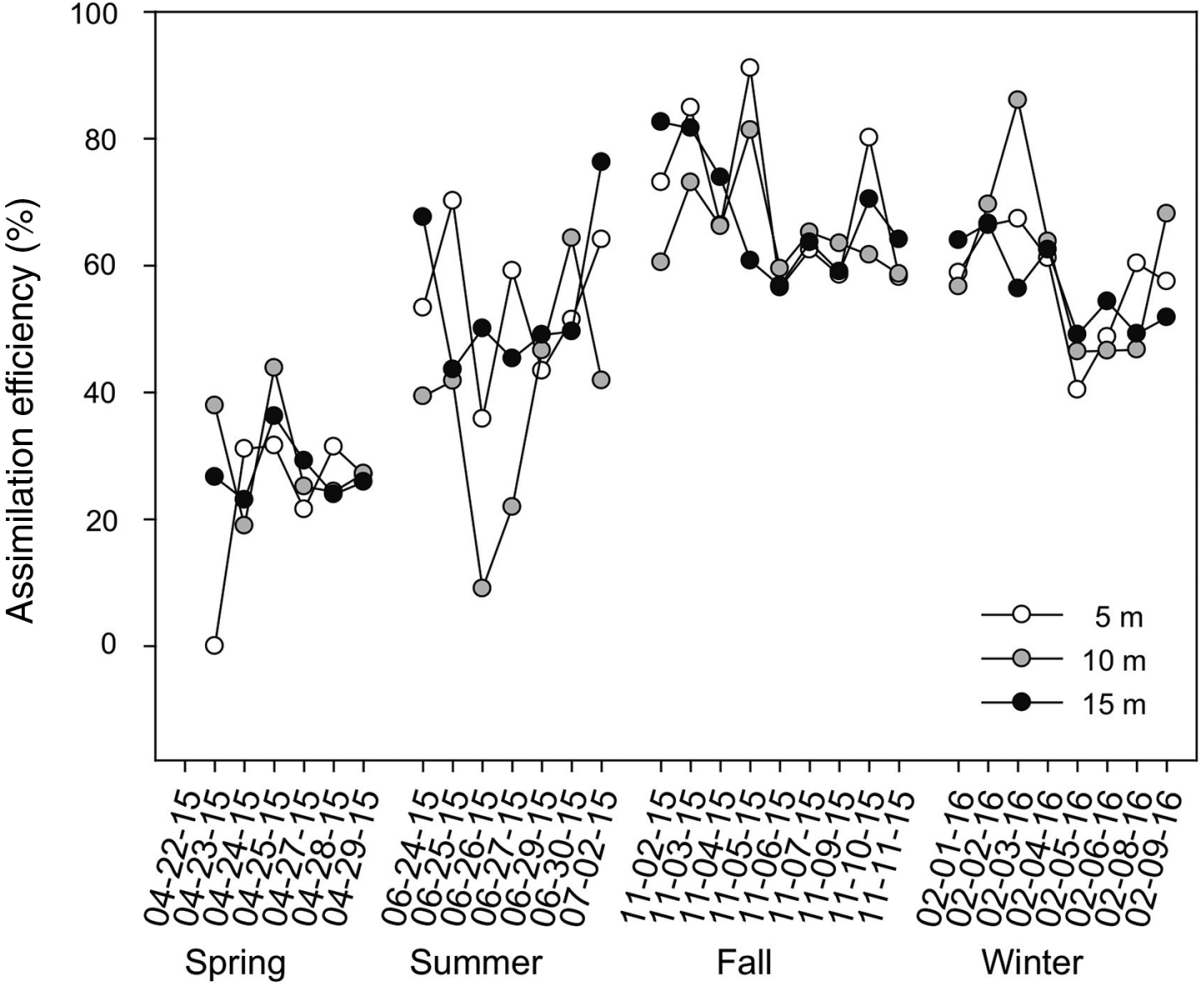
Taurine assimilation efficiency as a percentage of total uptake at the 5, 10, and 15 m depth horizon in the northern Adriatic Sea over a seasonal cycle.

The bulk prokaryotic community exhibited similar total taurine uptake rates (respiration + assimilation) from spring to fall (spring: 4.6 ± 1.4 nmol L^−1^ d^−1^, summer: 4.2 ± 2.0 nmol L^−1^ d^−1^, fall: 4.6 ± 1.4 nmol L^−1^ d^−1^; Fig. 3a). In winter, total taurine uptake rates were lower (winter: 2.6 ± 1.3 nmol L^−1^ d^−1^; Fig. 3a) than in the other seasons. Bulk taurine respiration rates (Supporting Information Fig. S6a) peaked in spring (3.5 ± 1.0 nmol L^−1^ d^−1^) and decreased significantly toward the winter (summer: 2.4 ± 2.0 nmol L^−1^ d^−1^, fall: 1.5 ± 0.7 nmol L^−1^ d^−1^, winter: 1.2 ± 0.8 nmol L^−1^ d^−1^; *p* ≤ 0.05). In contrast, bulk taurine assimilation (Supporting Information Fig. S6b) increased considerably from spring (spring: 1.4 ± 0.3 nmol L^−1^ d^−1^) toward the phytoplankton bloom in fall (3.1 ± 0.9 nmol L^−1^ d^−1^) (*p* ≤ 0.05), and decreased again in winter (1.4 ± 1.4 nmol L^−1^ d^−1^). Consequently, taurine assimilation efficiency (Fig. 4) was significantly higher in fall (68% ± 10%) as compared to the other seasons (spring: 29% ± 6%; summer: 49% ± 16%; winter 58% ± 10%, *p* ≤ 0.05). Bulk taurine assimilation correlated with the ambient taurine concentrations (*R* ^2^ = 0.60, Supporting Information Fig. S7), mainly driven by the higher taurine assimilation rates and concentrations in fall (*R* ^2^ = 0.62).

Leucine incorporation rates were highly variable in summer (5.1 ± 6.4 nmol L^−1^ d^−1^), with a strong peak observed at the beginning of the summer sampling period (Supporting Information Fig. S8). During fall, significantly higher rates (2.9 ± 0.6 nmol L^−1^ d^−1^, *p* ≤ 0.05) as compared to spring (1.2 ± 0.3 nmol L^−1^ d^−1^) and winter (0.6 ± 0.2 nmol L^−1^ d^−1^) were observed.

Taurine turnover rates (Supporting Information Table S9) based on prokaryotic taurine uptake were significantly higher in spring (4.8 ± 4.0 d^−1^) than in summer (1.4 ± 0.7 d^−1^) and fall (1.1 ± 0.9 d^−1^) (*p* ≤ 0.05), while during winter (2.2 ± 1.6 d^−1^) they increased, albeit not significantly. In contrast, leucine turnover rates (Supporting Information Table S9) were similar throughout spring (2.8 ± 2.2 d^−1^), summer (2.9 ± 1.3 d^−1^), and fall (3.2 ± 2.1 d^−1^). Only during the winter, leucine turnover rates were slightly lower (1.4 ± 1.2 d^−1^) than in the other seasons, albeit not significantly (*p* ≥ 0.05).

### Single‐cell taurine and leucine uptake by specific prokaryotic groups assessed by MICRO‐CARD‐FISH

Bulk taurine assimilation (Supporting Information Fig. S9a) and bulk leucine incorporation (Supporting Information Fig. S9b) correlated linearly with the silver grain area associated to the prokaryotic cells assimilating taurine and leucine, respectively, as assessed by MICRO‐CARD‐FISH.

**Fig. 5.**
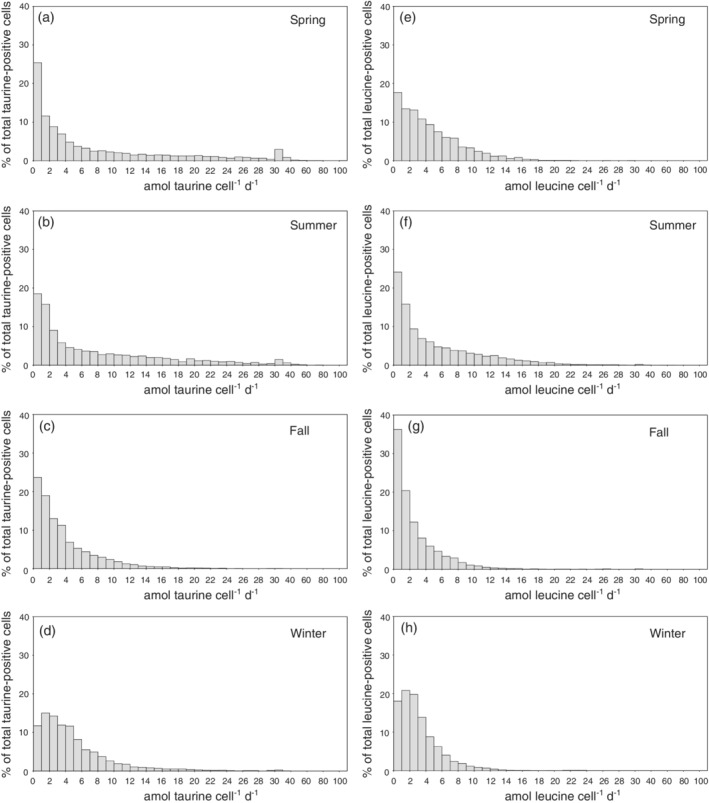
Distribution of single‐cell taurine (**a**–**d**) and leucine assimilation (**e**–**h**) determined by MICRO‐CARD‐FISH in the northern Adriatic Sea expressed as percentages of the abundance of DAPI‐stained cells assimilating taurine (**a**–**d**) and leucine (**e**–**h**) in different seasons.

**Fig. 6.**
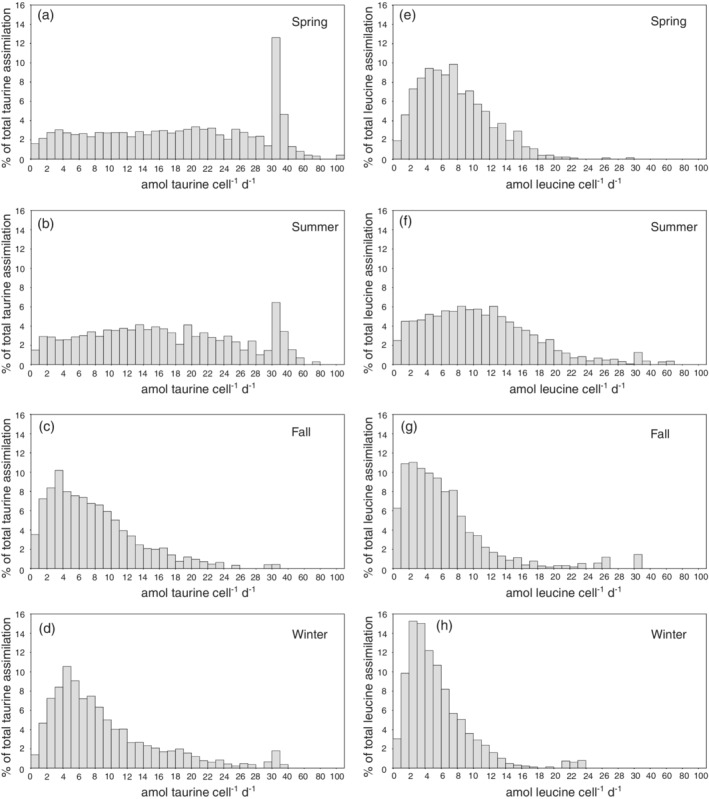
Distribution of cell‐specific taurine (**a**–**d**) and leucine assimilation (**e**–**h**) by the prokaryotic plankton determined by MICRO‐CARD‐FISH in the northern Adriatic Sea expressed as percentages of the total taurine (**a**–**d**) and leucine (**e**–**h**) assimilation in different seasons.

Twenty‐one to sixty five percent of the prokaryotic cells assimilated taurine, with lowest percentages in the summer and highest fractions in winter and spring (Supporting Information Table S10). Between 54% and 72% of the prokaryotic cells took up leucine (Supporting Information Table S10), with maximums in winter and summer. Approximately 70% (Fig. 5a,b) of all the cells assimilating taurine exhibited taurine assimilation rates between 0.1 and 10 amol taurine cell^−1^ d^−1^ during the spring and summer season, accounting for ∼ 25% of the total taurine assimilation (Fig. 6a,b). The remaining 30% of cells assimilated taurine at rates between 10 and 80 amol taurine cell^−1^ d^−1^ (Fig. 5a,b), accounting for ∼ 75% of the total taurine assimilation (Fig. 6a,b). During the fall and winter, 90% of the cells assimilating taurine exhibited taurine assimilation rates between 0.1 and 10 amol taurine cell^−1^ d^−1^ (Fig. 5c,d), accounting for ∼ 70% of total taurine assimilation (Fig. 6c,d).

More than 80% (Fig. 5e–h; spring: 91%; summer: 82%; fall and winter: 92%) of the cells assimilating leucine exhibited assimilation rates between 0.1 and 10 amol leucine cell^−1^ d^−1^, accounting for more than 70% of the total leucine assimilation in spring, fall, and winter (73%, 83% and 89%, respectively), and ∼ 49% in the summer (Fig. 6e–h).

The contribution of SAR11 to the total prokaryotic abundance ranged between 19% and 49%, whereas the *Roseobacter* clade and *Alteromonas* always contributed less than 10% to the prokaryotic abundance. The contribution of Thaumarchaeota varied between 5% and 9%, while Euryarchaeota contributed up to 20% to the total prokaryotic abundance (Supporting Information Table S11).

Bacteria contributed more to the taurine assimilating prokaryotes than they contributed to the total prokaryotic cell abundance in the summer, while in the other seasons, their contribution to the taurine‐assimilating prokaryotic community was similar to their contribution to the bulk prokaryotic cell abundance (Supporting Information Fig. S10a). Members of the SAR11 clade assimilating taurine contributed more to the taurine‐positive cells than to the bulk prokaryotic community in spring, summer, and fall. However, taurine‐positive SAR11 cells contributed a similar fraction to taurine assimilating cells and to the total prokaryotic cells in winter. The *Roseobacter* clade, *Alteromonas*, and Thaumarchaeota contributed equally to the taurine‐positive cells and to the bulk prokaryotic cells throughout all the seasons. Euryarchaeota also contributed similarly to the taurine‐positive cells and to the total prokaryotic cells, except in the summer, when Euryarchaeota contributed more to the taurine‐positive cells than to the total prokaryotic cells (Supporting Information Fig. S10a).

Bacteria and SAR11 also contributed more to leucine assimilating prokaryotes than to the bulk prokaryotic community (Supporting Information Fig. S10b). The other bacterial groups and the archaea contributed almost equally to the leucine assimilating cells and to the total prokaryotic cells throughout the seasons (Supporting Information Fig. S10b).

**Fig. 7.**
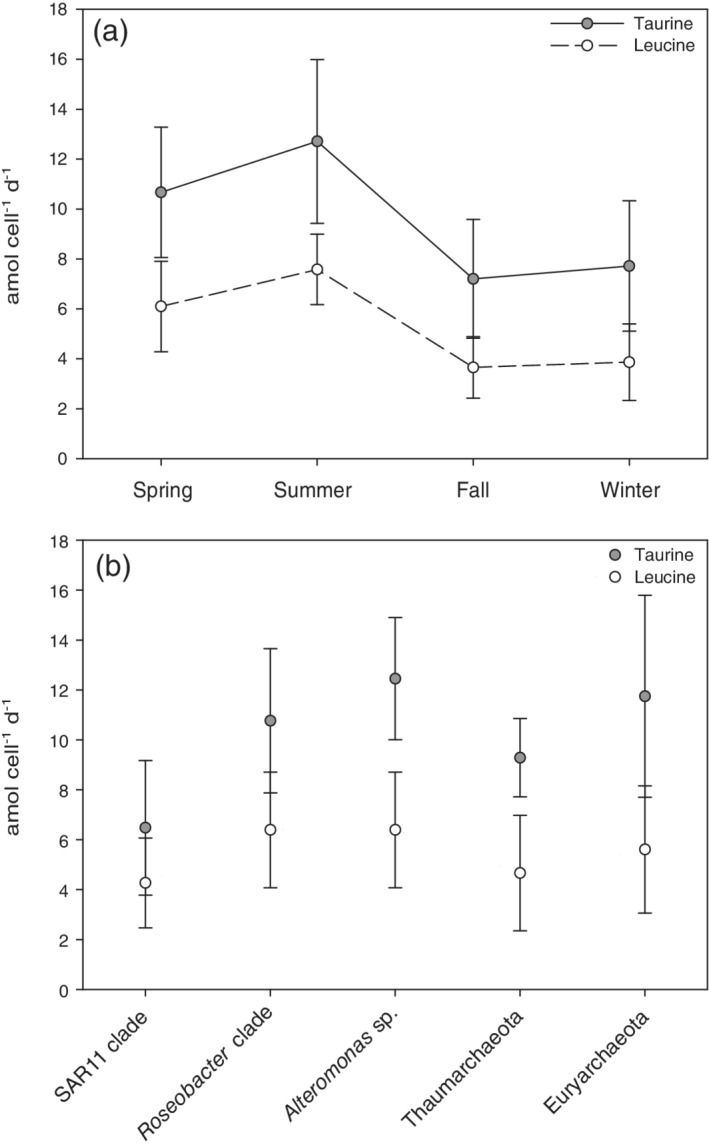
Cell‐specific taurine and leucine assimilation (**a**) over a seasonal cycle and of specific prokaryotic groups (**b**) averaged across all seasons.

The prokaryotic community assimilating taurine (Fig. 7a) showed significantly higher cell‐specific taurine assimilation in the summer (12.7 ± 3.3 amol taurine cell^−1^ d^−1^) and spring (10.7 ± 2.6 amol taurine cell^−1^ d^−1^) than in fall (7.2 ± 2.4 amol taurine cell^−1^ d^−1^) and winter (7.7 ± 2.6 amol taurine cell^−1^ d^−1^, *p* ≤ 0.05). The SAR 11 clade had the lowest single‐cell activity (6.5 ± 2.7 amol taurine cell^−1^ d^−1^), whereas the other prokaryotic groups assimilated taurine at rates between 9.3 ± 1.6 and 11.7 ± 4.0 amol taurine cell^−1^ d^−1^ (Fig. 7b).

In general, cells assimilated leucine (Fig. 7) at lower rates than taurine (Fig. 7). However, cells assimilating leucine exhibited a similar seasonal pattern (Fig. 7a) as for taurine assimilation (Fig. 7a), with a significantly higher assimilation rate during the summer (7.6 ± 1.4 amol leucine cell^−1^ d^−1^) and spring (6.1 ± 1.8 amol leucine cell^−1^ d^−1^) than in the fall (3.7 ± 1.2 amol leucine cell^−1^ d^−1^) and winter (3.9 ± 1.5 amol leucine cell^−1^ d^−1^; *p* ≤ 0.05).

Members of the SAR11 clade exhibited the highest bulk taurine assimilation rates compared to the other groups considering their abundance in the northern Adriatic Sea (Fig. 3b). Euryarchaeota cells exhibited higher bulk taurine assimilation from spring to fall than in winter (Fig. 3b). Members of the *Roseobacter* clade, *Alteromonas* sp., and Thaumarchaeota exhibited higher bulk taurine assimilation rates during the phytoplankton bloom in fall as compared to the other seasons (Fig. 3b).

Highest bulk leucine assimilation rates of SAR11 cells were determined for the summer period (Fig. 3c). The bulk leucine assimilation rates by members of the *Roseobacter* clade (Fig. 3c) were one order of magnitude higher in spring and fall than in the summer and winter. As also found for taurine, *Alteromonas* sp. (Fig. 3c) exhibited the highest leucine assimilation rates in the fall. Archaeal cells (Fig. 3c) showed lower bulk leucine assimilation rates in the winter than during the other seasons.

### Contribution of taurine‐C, ‐N, and ‐S to bulk prokaryotic biomass production and single cell activity of specific prokaryotic groups

**Fig. 8.**
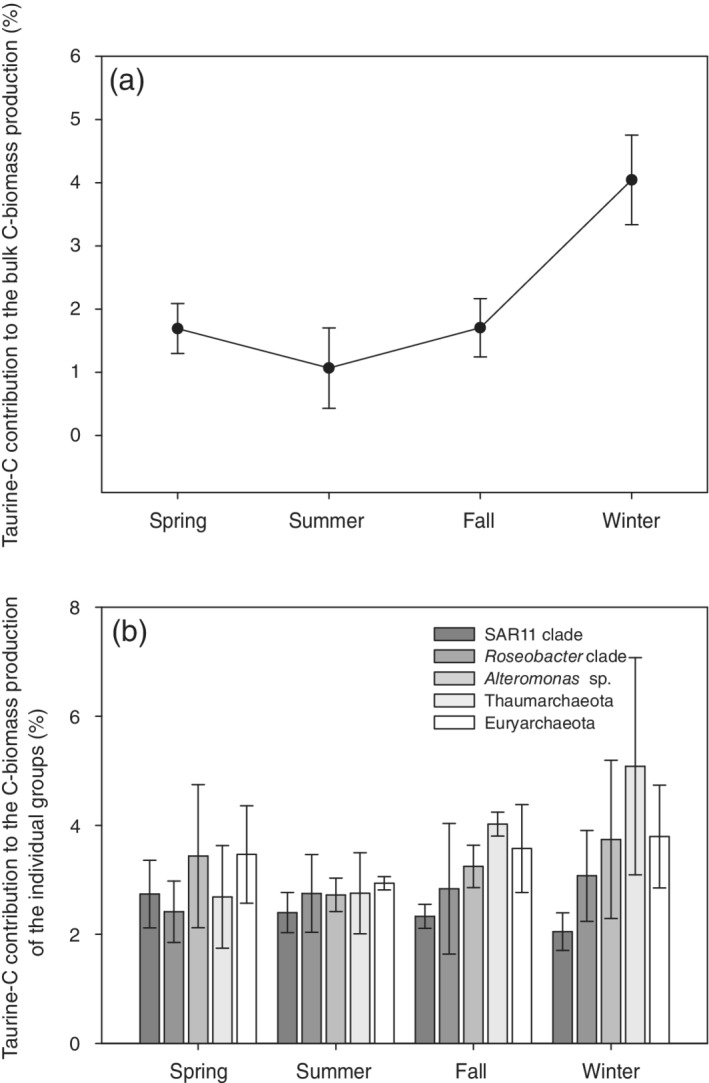
Contribution of taurine‐C to the prokaryotic bulk C‐biomass production measured by bulk leucine incorporation (**a**) and contribution of taurine‐C to the C‐biomass production of specific prokaryotic groups measured by MICRO‐CARD‐FISH (**b**) in the coastal northern Adriatic Sea over a seasonal cycle.

The potential contribution of taurine‐C to the prokaryotic bulk biomass production (Fig. 8a) and of taurine‐N and ‐S to the cell requirements (Supporting Information Table S12) was significantly higher in the winter (4.1% ± 0.7%, 8.1% ± 1.4%, and 52.6% ± 9.2%, respectively) than in the other seasons (*p* ≤ 0.05). In spring and fall, the contribution of taurine‐C, ‐N, and ‐S accounted for approximately 1.7% ± 0.43%, 3.4% ± 0.9%, and 22.1% ± 5.6% for the C, N, and S requirements of the bulk prokaryotic community, respectively. The lowest contribution of taurine‐C, ‐N, and ‐S to the C, N, and S requirements of the prokaryotic community was observed in the summer (Fig. 8a, Supporting Information Table S12).

Taurine‐C contributed between 2% and 5% to the biomass production of the different prokaryotic groups (Fig. 8b). The contribution of taurine‐N to the cell requirements accounted for less than 11%, whereas taurine‐S contribution ranged between 26% and 60% among different prokaryotic groups (Supporting Information Table S13). In general, the contribution of taurine‐C, ‐N, ‐S to the cell requirements was lowest in SAR11 and *Roseobacter* cells. In *Alteromonas* and archaea, the contribution of taurine‐C, ‐N, ‐S to the cell requirements increased toward the fall and winter.

## *Discussion*

### Relationship between crustacean zooplankton release of taurine and DFAA and their utilization by the prokaryotic community

The northern Adriatic Sea is a highly dynamic system influenced by riverine freshwater discharge and the oligotrophic central Adriatic waters (Kraus et al. 2016).

In general, DFAA (Supporting Information Table S8) and taurine concentrations (Fig. 1a) showed strong short‐term (daily) variations as previously reported for the northern Adriatic Sea (Müller‐Niklas et al. 1994) and other coastal areas (Lu et al. 2014). The considerably higher total DFAA (Supporting Information Table S8) and taurine concentrations (Fig. 1a, Supporting Information Table S8) measured in fall than in the other seasons presumably originate from crustacean zooplankton release (Fig. 2a,b and Supporting Information Table S3) and the phytoplankton bloom dominated by different *Pseudonitzschia* species (> 70%) and other diatoms (Steiner et al. 2019) known to produce taurine (Jackson et al. 1992; Durham et al. 2019). Phytoplankton blooms, often dominated by *Pseudonitzschia* species (Marić et al. 2011), are a common feature of the northern Adriatic Sea in the fall–winter period, provoked by vertical mixing and input of inorganic nutrients from the seafloor (Kraus et al. 2016). During the fall–winter period, small‐sized phytoplankton (picoeukaryotes, *Synechococcus*, and/or *Prochlorococcus*) were also more abundant than in the spring–summer period, (Supporting Information Figs. S3, S4) coinciding with the occurrence of small‐sized copepods (Supporting Information Table S1) and higher nitrite concentrations as previously reported (Mozetič et al. 2010; Šilović et al. 2012).

In the Gulf of Alaska, elevated taurine release rates of amphipods have been related to their carnivorous diet (Clifford et al. 2017). Also Saba et al. (2009) showed that *Acartia* sp. released dissolved organic carbon (DOC) at higher rates when feeding on a carnivorous plankton than on a phytoplankton or omnivorous diet. High dissolved organic nitrogen (DON) release was observed when zooplankton were feeding on toxic algae, possibly increasing their membrane permeability (Saba et al. 2011). Hence, although crustacean zooplankton are synthesizing taurine, the elevated taurine and DFAA release rates during the fall–winter period might also be attributed to taurine‐ and DFAA‐enriched diet and/or harmful phytoplankton. Additionally, the quality, quantity, composition, and the size of the available diet can strongly influence the release rates of DOM compounds by copepods (Frangoulis et al. 2004; Valdés et al. 2017) and thus, the organic C, N, and S available to heterotrophic prokaryotes (Valdés et al. 2018*a*,*b*). The diversity of copepod species was higher in the fall–winter period, especially of small‐sized species, than in the spring–summer period (Supporting Information Table S1). Smaller animals tend to exhibit higher biomass‐normalized release rates (Verity 1985; Hall et al. 2007), in agreement with our results (Supporting Information Table S1). In general, release rates have been shown to increase with increasing temperature (Bayne and Scullard 1977). However, the high taurine release rates during the winter, when temperature was low (∼ 10°C), imply that temperature was not the main factor determining taurine release rates. Taurine as cryoprotectant (Loomis et al. 1988) could favor higher taurine concentrations in organisms exposed to low temperature conditions, potentially resulting also in higher taurine release rates. However, further studies are needed to elucidate the physiological roles of taurine in copepods. Notably, taurine release rates per C‐biomass are higher (up to 240 times) in the northern Adriatic than in the open ocean (Clifford et al. 2017), probably also linked to the higher quantity and quality of the food available in the shallow northern Adriatic (Frangoulis et al. 2004).

Taurine assimilation efficiencies were highest during the fall–winter period, when heterotrophic processes presumably predominated (Solidoro et al. 2007). Particularly in the fall, the prokaryotic community responded to the higher taurine availability (Supporting Information Fig. S7). The percentage of taurine‐C respired (0–91%; Fig. 3a) of the total taurine taken up varied considerably on a daily and seasonal basis, similar to DFAA respiration (3–80%) in coastal systems (Suttle et al. 1991). This indicates a high variability within the prokaryotic community in their capacity and requirements to utilize taurine (and DFAAs) as a C‐ and energy source.

The higher nitrate and silicate concentrations (Supporting Information Table S7) during the late spring–summer period point to a high freshwater input and low exchange with the oligotrophic central Adriatic in these two seasons (Giani et al. 2012; Šilović et al. 2012) supporting high cell‐specific taurine and leucine assimilation rates (Fig. 7). This coincided with an increase in cell‐specific extracellular enzymatic activity (Ivančić et al. 2018) and with changes in the prokaryotic community composition (Steiner et al. 2019). Moreover, changes in prokaryotic cell volume and metabolism have been reported to also occur during these seasons in the coastal Adriatic Sea (La Ferla and Leonardi 2005; Paoli et al. 2006). Although the prokaryotic abundance was lower during the spring–summer than during the fall–winter period, total taurine uptake rates (respiration + assimilation) were similar to fall and higher than in the winter (Fig. 3a), likely as a consequence of higher cell‐specific taurine and leucine assimilation rates (Fig. 7) and larger prokaryotic cell volumes (La Ferla and Leonardi 2005).

Bulk release rates by copepods were compared to the bulk prokaryotic taurine and leucine uptake to estimate whether taurine and leucine released by copepods potentially match the taurine or leucine uptake of the prokaryotic community. During the fall–winter period, copepods supplied sufficient taurine (fall: 10.8 ± 6.8 nmol L^−1^ d^−1^; winter: 8.3 ± 6.0 nmol L^−1^ d^−1^; Fig. 2c) to cover the demand of the prokaryotic community (fall: 4.6 ± 1.4 nmol L^−1^ d^−1^, winter: 2.6 ± 1.3 nmol L^−1^ d^−1^; Fig. 3a,b). The taurine supplied by copepods during the spring–summer period (spring: 1.9 ± 2.4 nmol L^−1^ d^−1^, summer: 1.9 ± 0.9 nmol L^−1^ d^−1^, Fig. 2c) contributed only partly to the taurine uptake of the prokaryotic community (spring: 4.6 ± 1.4 nmol L^−1^ d^−1^, summer: 4.2 ± 2.0 nmol L^−1^ d^−1^; Fig. 3a,b). Therefore, other sources (e.g., phytoplankton, other abundant metazoans) most likely contributed to match the prokaryotic taurine uptake in these seasons. Leucine was only released occasionally by the incubated crustacean zooplankton (Supporting Information Tables S3, S4), especially during the spring–summer period. Thus, the bulk leucine release rates (spring: 0.6 nmol L^−1^ d^−1^ summer: 0.7 ± 0.3 nmol L^−1^ d^−1^; Fig. 2c) were not sufficient to cover the leucine demand of the prokaryotic plankton in these seasons (spring: 1.2 ± 0.3 nmol L^−1^ d^−1^, summer: 5.1 ± 6.2 nmol L^−1^ d^−1^; Fig. 3c, Supporting Information Fig. S8). Therefore, phytoplankton and/or microbial cleavage of proteins were likely the major leucine sources. During the fall–winter period, leucine supplied by copepods (fall: 2.4 ± 2.1 nmol L^−1^ d^−1^, winter: 1.6 ± 1.4 nmol L^−1^ d^−1^; Fig. 2c) could potentially meet the leucine requirements of the prokaryotic community (fall: 2.9 ± 0.6 nmol L^−1^ d^−1^, winter: 0.6 ± 0.2 nmol L^−1^ d^−1^; Fig. 3c, Supporting Information Fig. S8).

Similar taurine turnover rates based on bulk prokaryotic uptake (1.1–4.8 d^−1^ on average) and on estimated bulk copepod release rates (1.2–5.3 d^−1^ on average) were obtained despite the fact that copepod abundance data were derived from the literature. However, the highest taurine turnover rates calculated based on bulk prokaryotic uptake were obtained in the spring while those based on the bulk copepod release rates were obtained in the winter (Supporting Information Table S9), and taurine turnover rates on individual dates did not correlate (*p* > 0.05). Copepod abundance is intra‐ and interannually variable, depending on fluctuations in environmental parameters particularly in the northern Adriatic Sea (Kamburska and Fonda‐Umani 2006; Vukanic et al. 2018). Furthermore, other important sources of taurine and leucine, such as phytoplankton and other metazoans, are not included in these calculations. It has been shown that bivalves, sponges, ascidians, and scyphozoan are also important sources of taurine (Allen and Garrett 1971). All these metazoan groups are at least seasonally abundant in the northern Adriatic Sea.

### Taxa‐specific utilization of taurine and leucine

The fraction of total prokaryotic cells assimilating taurine (21–60%) is within the range of cells taking up DMSP and DFAAs (32–61%) (Malmstrom et al. 2004*a*,*b*) pointing to an important role of taurine as a substrate for heterotrophic prokaryotes in the northern Adriatic. The larger and less variable contribution of cells assimilating leucine to the total prokaryotic community (> 55%; Supporting Information Table S10) indicates the well‐known widespread utilization of leucine by heterotrophic bacteria (Nikrad et al. 2012). However, the examined prokaryotic groups exhibited higher cell‐specific taurine than leucine assimilation (Fig. 7). Taurine can serve as a C, N, S and energy source for prokaryotes and its catabolism can lead to other metabolically important organic and inorganic compounds (e.g., thiosulfate, sulfate, ammonium, alanine, or other organosulfonates) (Cook and Denger 2006).

Members of all investigated groups (*Roseobacter* clade, *Alteromonas*, Thaumarchaeota, and Euryarchaeota) exhibited, on average, higher cell‐specific assimilation rates of taurine than the more abundant SAR11 clade, consistent with previous findings on these groups (Alonso and Pernthaler 2006). Several studies also indicated that SAR11 might be relatively less active in coastal waters than in open ocean waters since they are adapted to low nutrient conditions (Malmstrom et al. 2004*a*; Alonso and Pernthaler 2006). In contrast, members of the *Roseobacter* clade, *Alteromonas*, and Euryarchaeota have been frequently reported to be stimulated by elevated DOM and/or particulate organic matter availability supplied by phytoplankton (Sarmento and Gasol 2012; Needham and Fuhrman 2016) and copepods (Valdés et al. 2017, 2018*b*). However, taking the abundance of SAR11 into account, the SAR11 clade contributed most to the bulk taurine and leucine assimilation (Fig. 3b,c).

The quantity and quality of the DOC pool can act as a selective force structuring bacterioplankton communities, whereby different phylo‐ and ecotypes can strongly differ in their substrate preferences and affinities (Gómez‐Consarnau et al. 2012). The high variability in cell‐specific taurine and leucine assimilation (Figs. 5., 6., Supporting Information Fig. S11) points to the presence of either different species or ecotypes within the different taxa studied exhibiting different efficiencies in utilizing these labile compounds and different seasonal patterns. The *Roseobacter* DC5‐80‐3 cluster has been described to consist of at least two ecotypes, one harboring taurine utilization systems and the other lacking them (Sun et al. 2017). Thaumarchaeota MG I and Euryarchaeota MGIIb in the winter and Euryarchaeota MGIIa in the summer exist in various ecotypes in the coastal surface waters of the western Mediterranean Sea, characterized by different activity patterns (Hugoni et al. 2013). Furthermore, several recent studies on the Euryarchaeota MGII group in coastal and open ocean systems identified diverse subclades with different ecological niches and lifestyles including members able to utilize taurine (Orellana et al. 2019; Pereira et al. 2019). *Alteromonas* phylo‐ and ecotypes also drastically differ in their utilization efficiencies of specific C‐compounds (Gómez‐Consarnau et al. 2012; Sarmento and Gasol 2012).

### Contribution of taurine to C‐, N‐, and S‐ biomass production of different prokaryotic taxa

The C supplied by taurine accounted for up to 5% of the bulk prokaryotic biomass production in the northern Adriatic (Fig. 8). This potential contribution of taurine‐C to the heterotrophic prokaryotic biomass production is considerably lower than in the open ocean (∼ 21%) (Clifford et al. 2019), most probably due to a higher availability of diverse autochthonous and allochthonous C‐sources in coastal areas (Alonso and Pernthaler 2006; Gómez‐Consarnau et al. 2012) as compared to the open ocean. Similar results were obtained for DMSP‐C, which supplies 0.5–6% of the total C demand of heterotrophic prokaryotes at a coastal site of the NW Mediterranean Sea (Simó et al. 2009) and up to 15% at open ocean sites (Simó et al. 2002). Taurine‐C accounted for 2–3% of the C‐biomass production in SAR11 and up to 5% in the other groups, similar to the estimated contribution of DMSP‐C (approximately 7% of the C‐demand of SAR11 cells; Tripp et al. 2008). Interestingly, particularly in archaea (Euryarchaeota and Thaumarchaeota), the potential contribution of taurine‐C to the archaeal biomass production is, on average, considerably higher in the fall–winter period than in the late spring–early summer period (Fig. 8b). Thaumarchaeota are assumed to be chemoautotrophic (Könneke et al. 2005); however, members of this taxa are able to take up organic compounds as C‐source such as DFAAs and taurine (Ouverney and Fuhrman 2000; Sauder et al. 2017), suggesting that mixotrophic or even heterotrophic lifestyles also occur in these archaeal groups. Veuger et al. (2013) suggested that the heterotrophic acquisition of C by Thaumarchaeota and other nitrifiers might be a relevant process in DOM‐rich coastal waters. Moreover, taurine might be an important ammonia source for some Thaumarchaeota species and/or ecotypes, as shown for other organic N‐compounds (Damashek et al. 2019) since the utilization of taurine can lead to the release of ammonium that might serve as an energy source. Among other organic C‐substrates, taurine stimulates growth in *Nitrosocosmicus exaquare*, thereby accelerating its ammonium‐oxidizing activity (Sauder et al. 2017). Valdés et al. (2018*b*) showed that ammonia oxidizing archaea are highly active in coastal waters off central Chile in winter in response to DON released by copepods, outcompeting ammonia‐oxidizing bacteria at ammonia concentrations close to the detection limit.

Some prokaryotic groups (e.g., members of the SAR11 clade) lack the ability to utilize sulfate as a S‐source (Tripp et al. 2008) or preferentially utilize organic sulfur compounds such as taurine over sulfate (e.g., Actinobacteria) (Chien et al. 1999). Estimates of the contribution of taurine‐S to the cell S‐requirements (2–71%, Supporting Information Table S12) are also in the range of previous estimates for DMSP‐S (3–100%) for natural prokaryotic communities from a Mediterranean costal site (Simó et al. 2009). However, DMSP‐S can potentially meet more than 300% of the S demand in SAR11 cells (Tripp et al. 2008), while taurine‐S only covers ∼ 30% of the S‐requirements of SAR11 (this study). The estimated DMSP‐S contribution to the total S‐demand of SAR11 is derived from culture studies (Tripp et al. 2008). However, the natural SAR11 populations likely consist of different ecotypes, with potentially different requirements, affinities, and capabilities to take up and utilize taurine (Supporting Information Fig. S11). The percentage of prokaryotic cells assimilating taurine was lowest during the summer (Supporting Information Table S10) coinciding with high DMSP concentrations (Steiner et al. 2019). In contrast, during the fall–winter period, low DMSP (Steiner et al. 2019) and high taurine concentrations (Fig. 1a) coincided with a high contribution of cells assimilating taurine (Supporting Information Table S10). This suggests seasonal shifts in the preferred organic S‐compounds, potentially provoking changes in the prokaryotic community composition (Kiene et al. 1999). The general shift toward small‐sized copepods and phytoplankton (Kamburska and Fonda‐Umani 2006; Mozetič et al. 2010), presumably a result of rising temperature, might favor the predominance of taurine over DMSP in a future ocean since DMSP is mainly produced by larger phytoplankton (Durham et al. 2019).

Overall, in the present study we showed that copepods, a main component of the marine mesozooplankton community, release copious amounts of taurine supporting heterotrophic taurine metabolism of the prokaryotic community. Hence, copepods, among many other marine metazoans, are a main taurine source for the marine heterotrophic prokaryotic community in marine surface waters. The release of taurine by copepods, however, might also play an important role in the dark ocean where phytoplankton are absent. Additionally, copepods can extensively contribute to nutrient cycling as they release other DON compounds such as DFAAs (e.g., serine, aspargine, glycine, alanine). Taurine accounted for a lower fraction of the C‐biomass production (up to 5%) and N cell‐requirements of coastal prokaryotic communities (up to 11%) as compared to the open ocean (C: ∼ 21% in epipelagic water; N: ∼ 38%), likely as a consequence of a more diverse DOC and DON pool in coastal regions. The contribution of taurine to heterotrophic prokaryotic biomass production, however, is similar to that of total DFAAs (Kroer et al. 1994). Additionally, taurine can potentially sustain a similar proportion of the cell's S‐requirements (up to 71%) in the coastal northern Adriatic Sea as DMSP‐S (Simó et al. 2009) in marine prokaryotic communities indicating its potential significance in coastal S‐cycling.

## Conflict of Interest

None declared.

## Supporting information


**Figure S1** Supporting information

**Table S1** Supporting information
