# Supplementary material for: Mesozooplankton taurine production and prokaryotic uptake in the northern Adriatic Sea
Source: Limnol Oceanogr. 2020 Jun 25;65(11):2730–47. doi: 10.1002/lno.11544 (PMC7891661; doi:10.1002/lno.11544)
Supplement: Supplementary file 1 — Figure S1 Supporting information [file LNO-65-2730-s001.pdf]

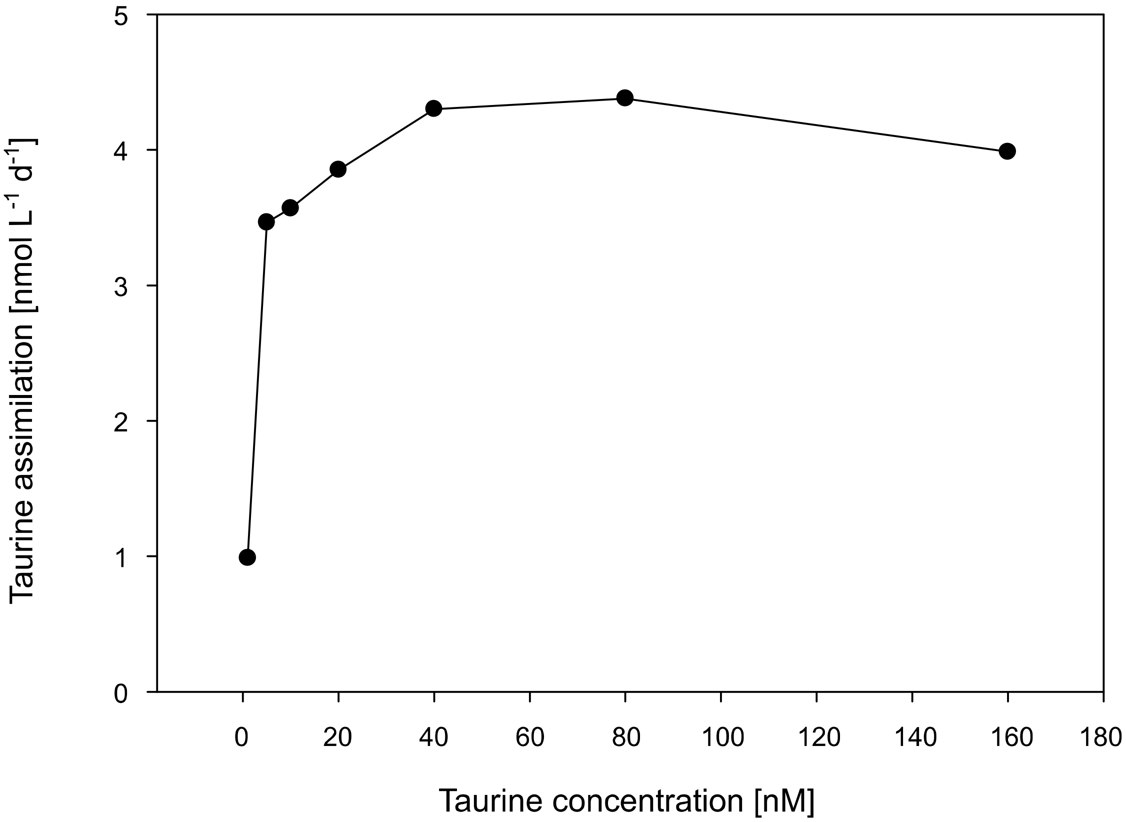

Figure S1. Prokaryotic taurine assimilation rates versus taurine concentrations in the coastal northern Adriatic Sea.

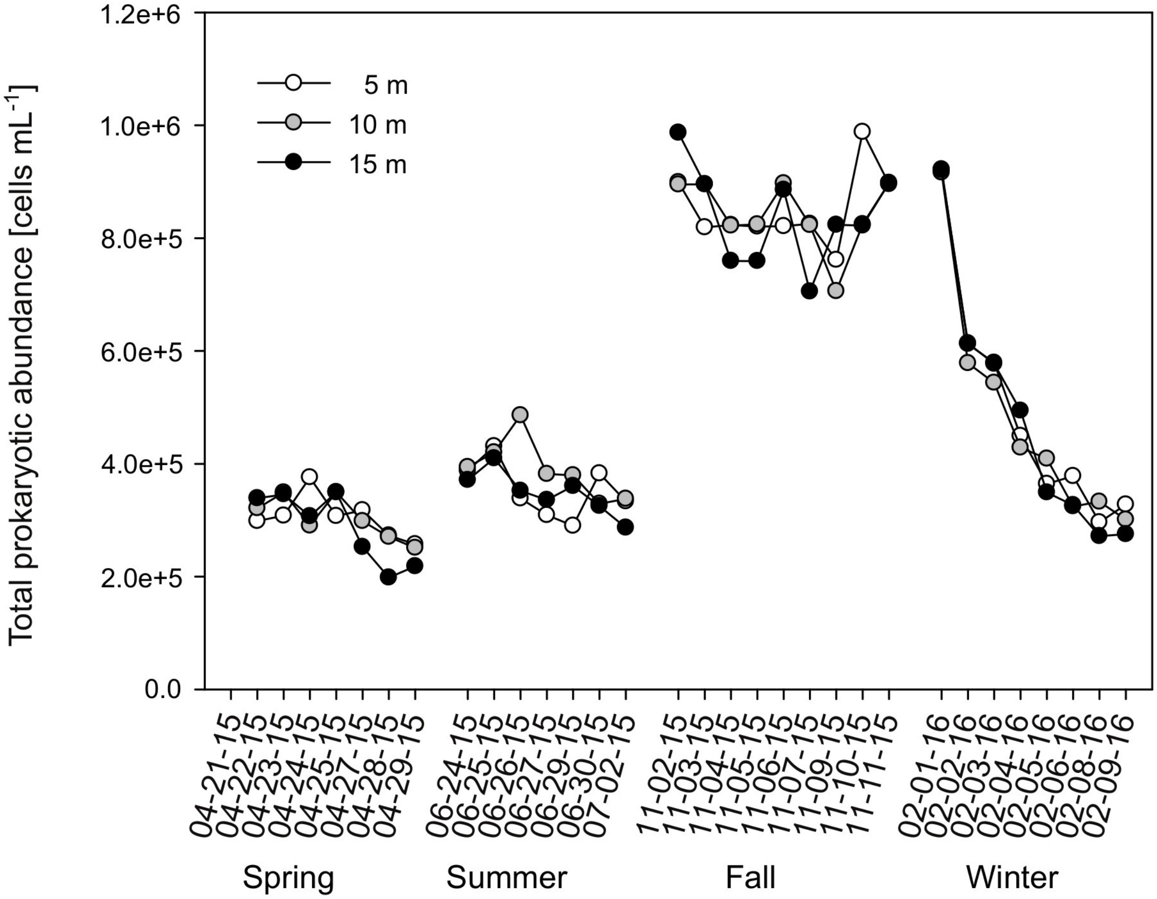

Figure S2. Total prokaryotic abundance in the coastal northern Adriatic Sea.

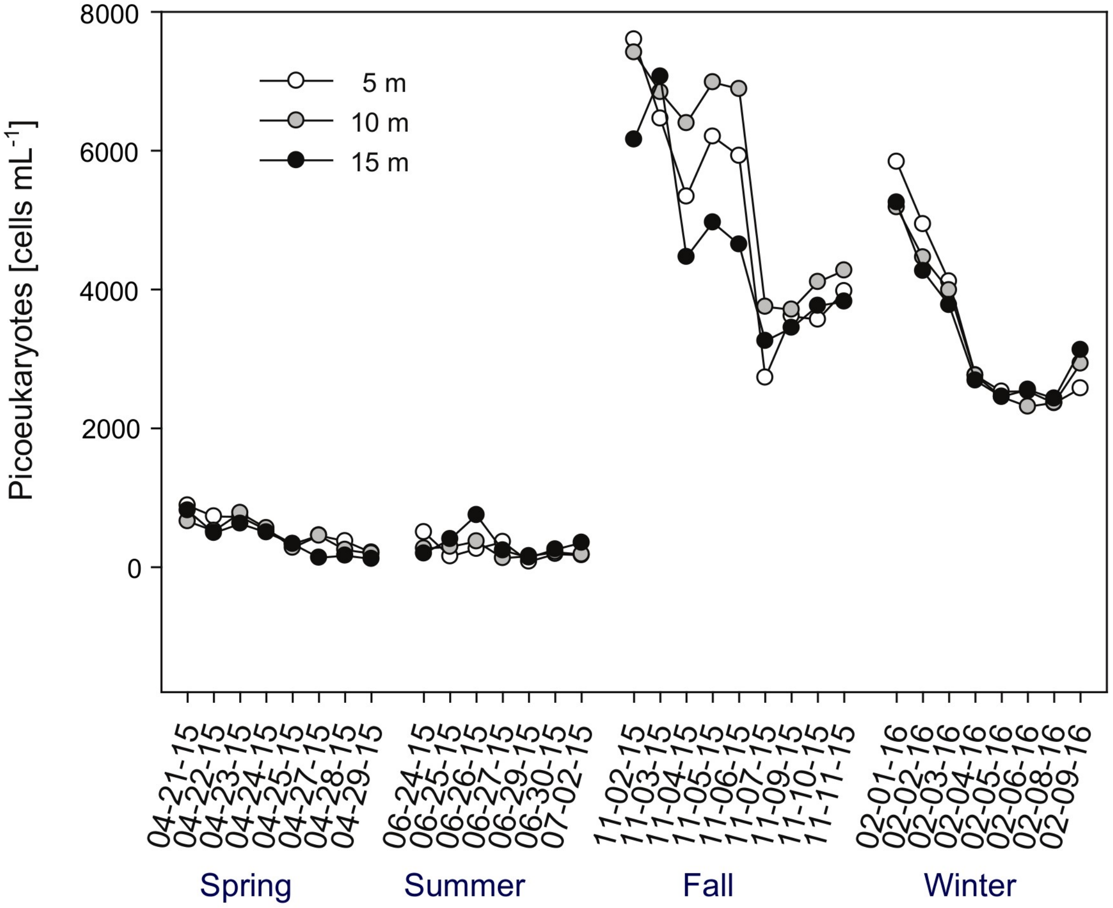

Figure S3. Picoeukaryotic abundance in the coastal northern Adriatic Sea.

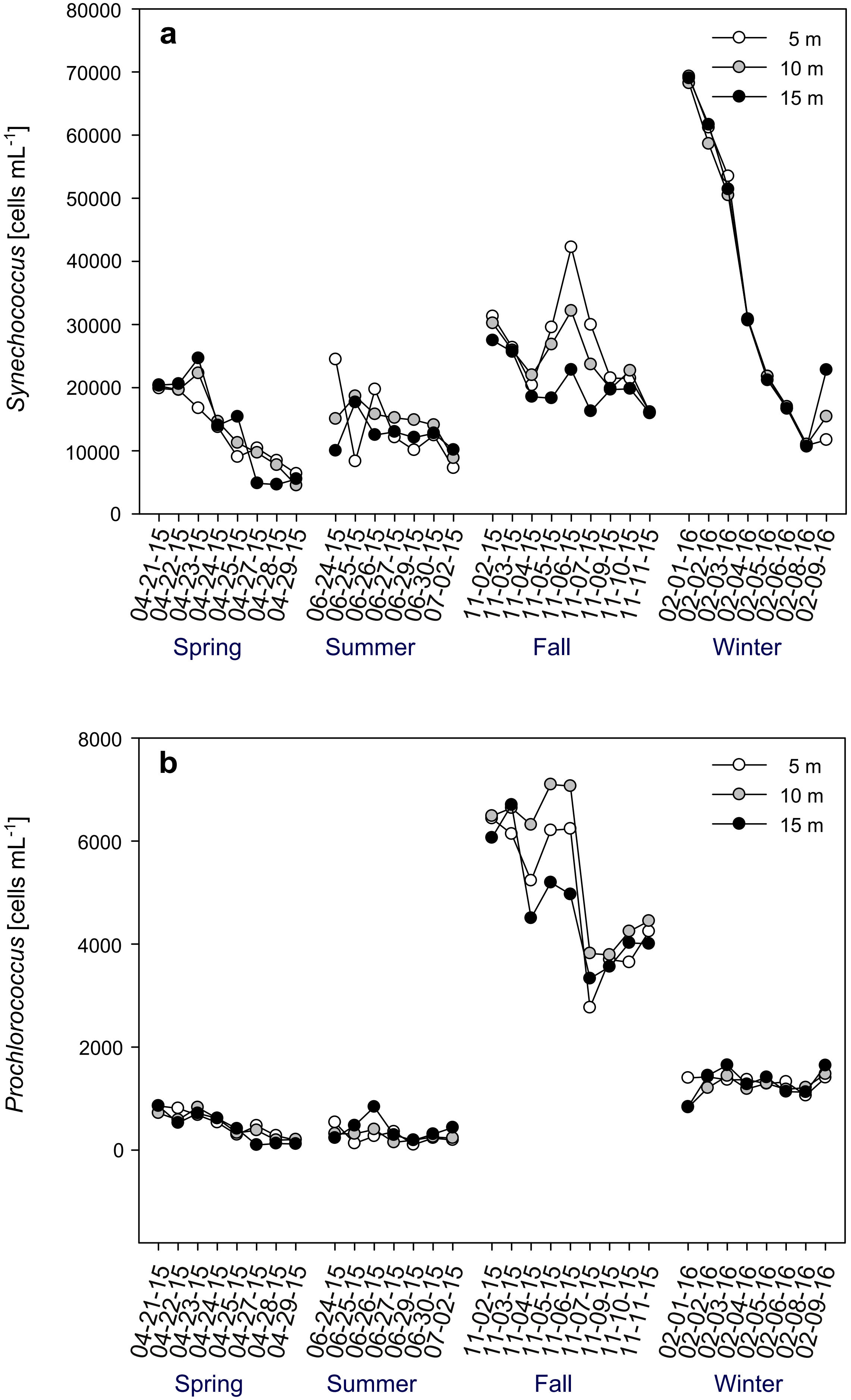

Figure S4. Abundance of *Synechococcus* (a) and *Prochlorococcus* (b) in the coastal northern Adriatic Sea.

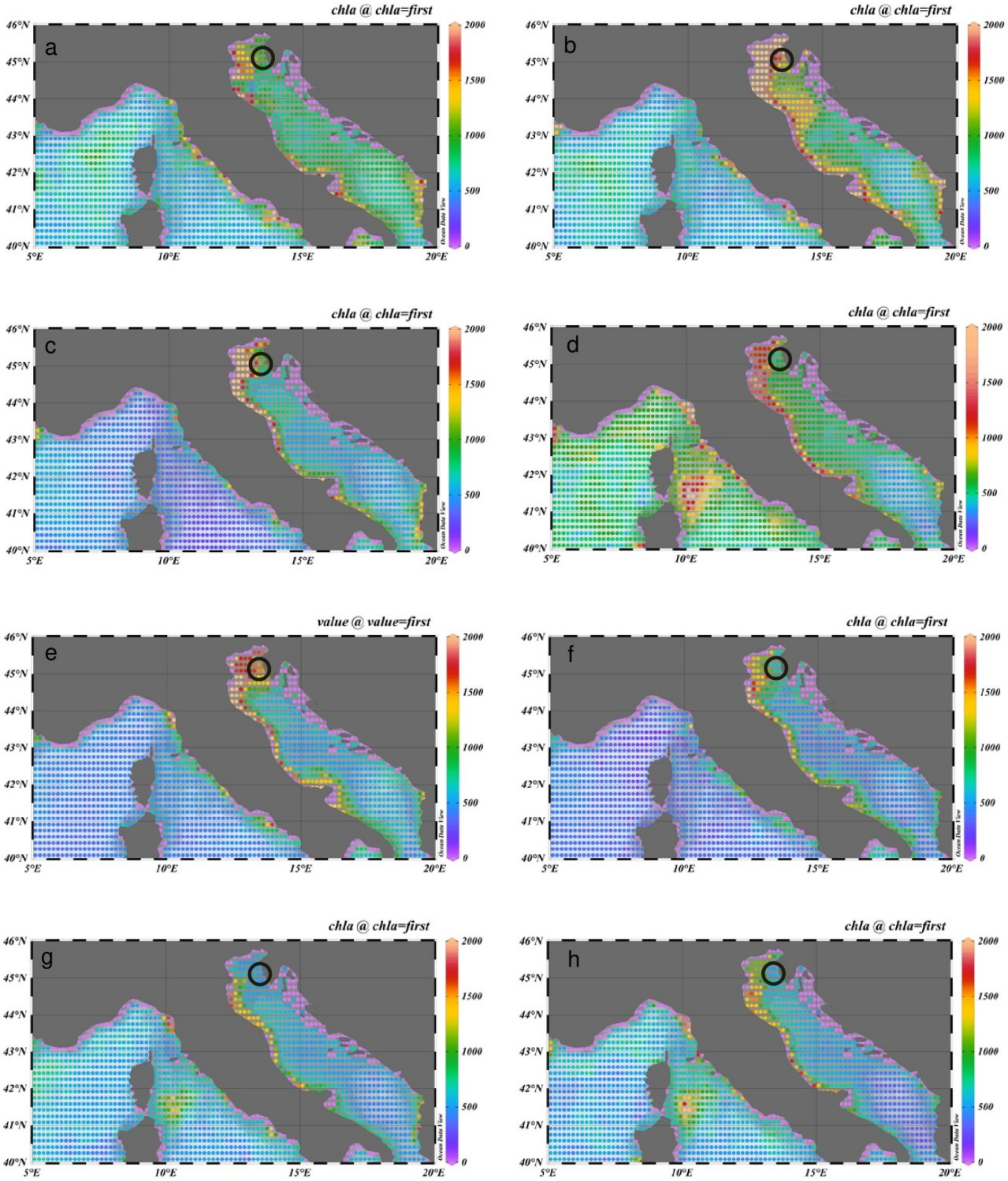

Figure S5. Weekly mean primary production (mg C m^{-2} d^{-1}) at the sampling location, retrieved from the Ocean Productivity website (http://www.science.oregonstate.edu/ocean.productivity/): April (a: 20/04/2015 - 26/04/2015; b: 04/27/2015 - 05/02/2015), June (c: 06/22/2015 - 05/28/2015; d: 05/29/2015 - 06/05/2015), November (e: 11/02/2015 -11/08/2015; f: 11/09/2015 - 11/15/2015), February (g: 02/01/2016 - 02/07/2016; h: 02/08/2016 - 02/14/2016).

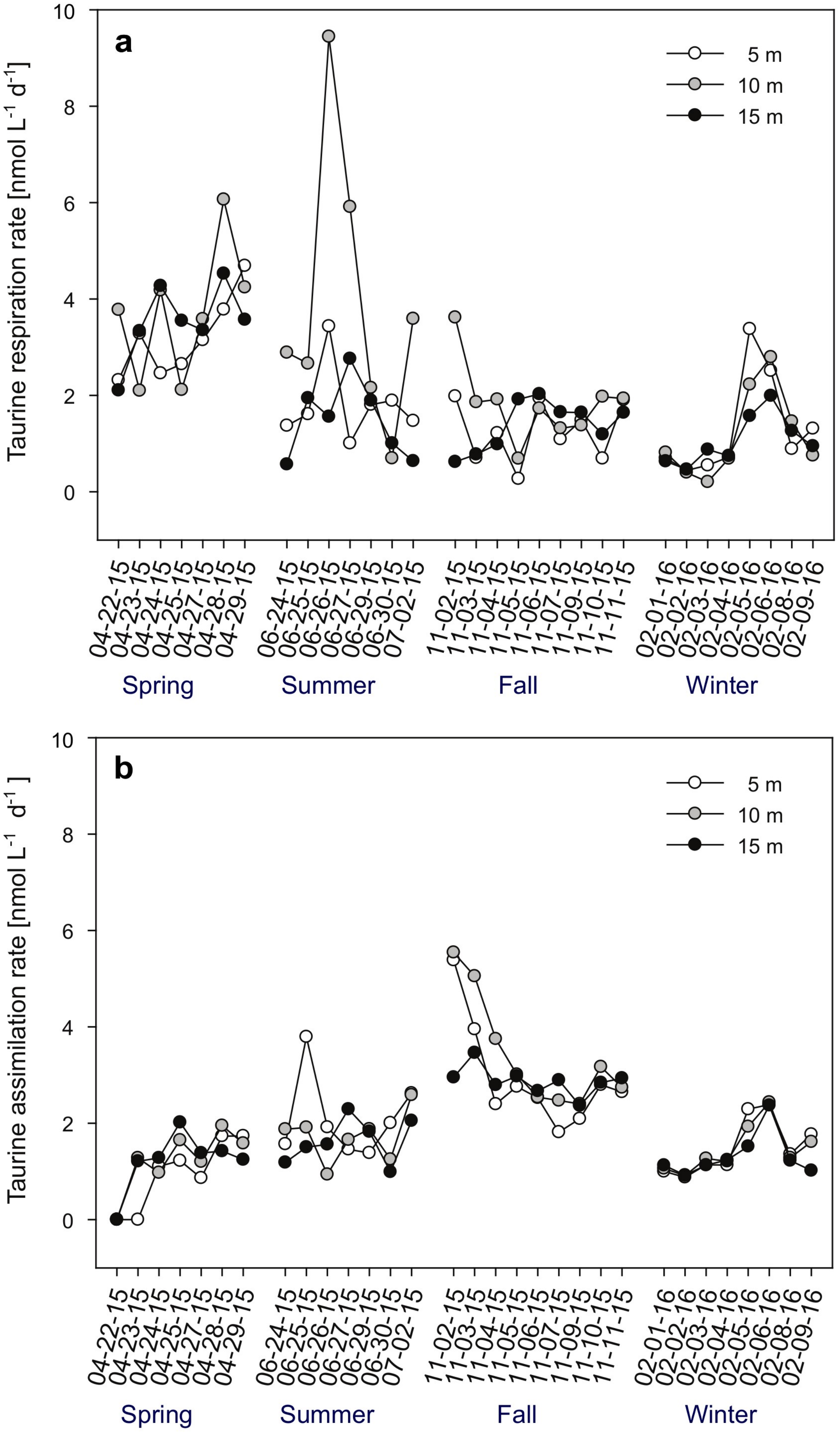

Figure S6. Taurine respiration (a) and assimilation rates (b) in the coastal northern Adriatic Sea.

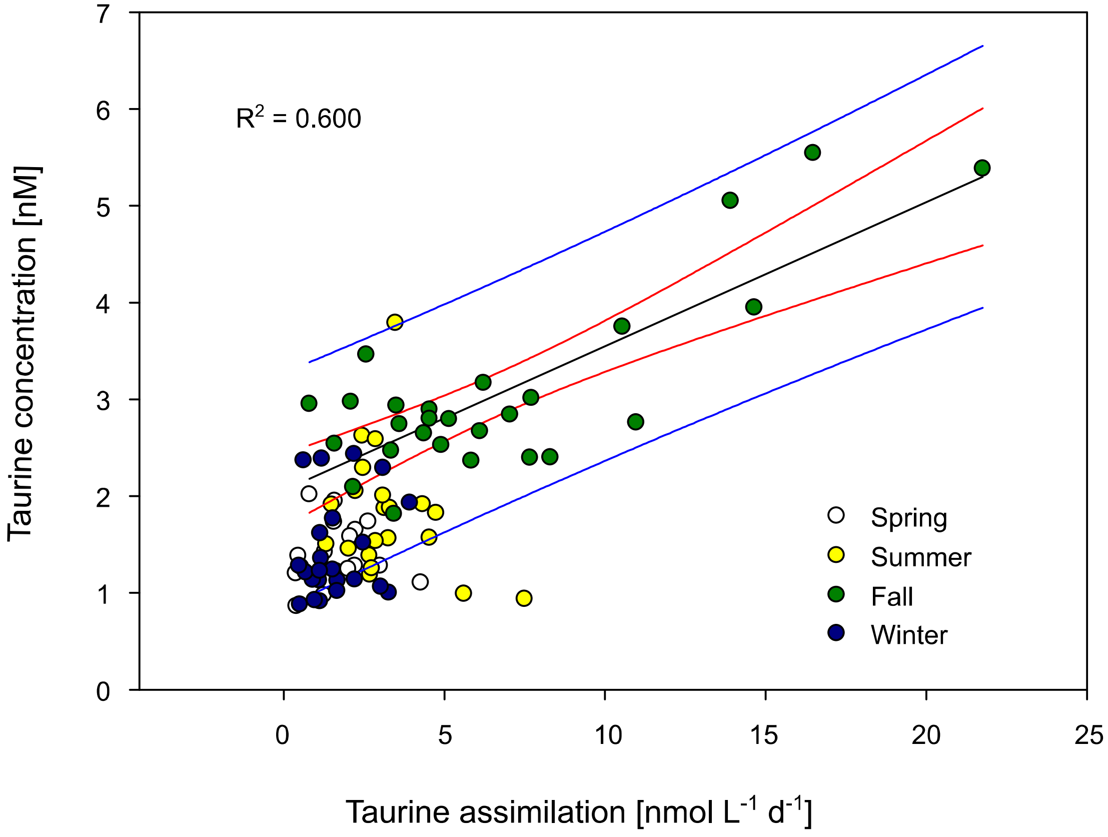

Figure S7. Taurine concentrations versus taurine assimilation rates in the coastal northern Adriatic Sea.

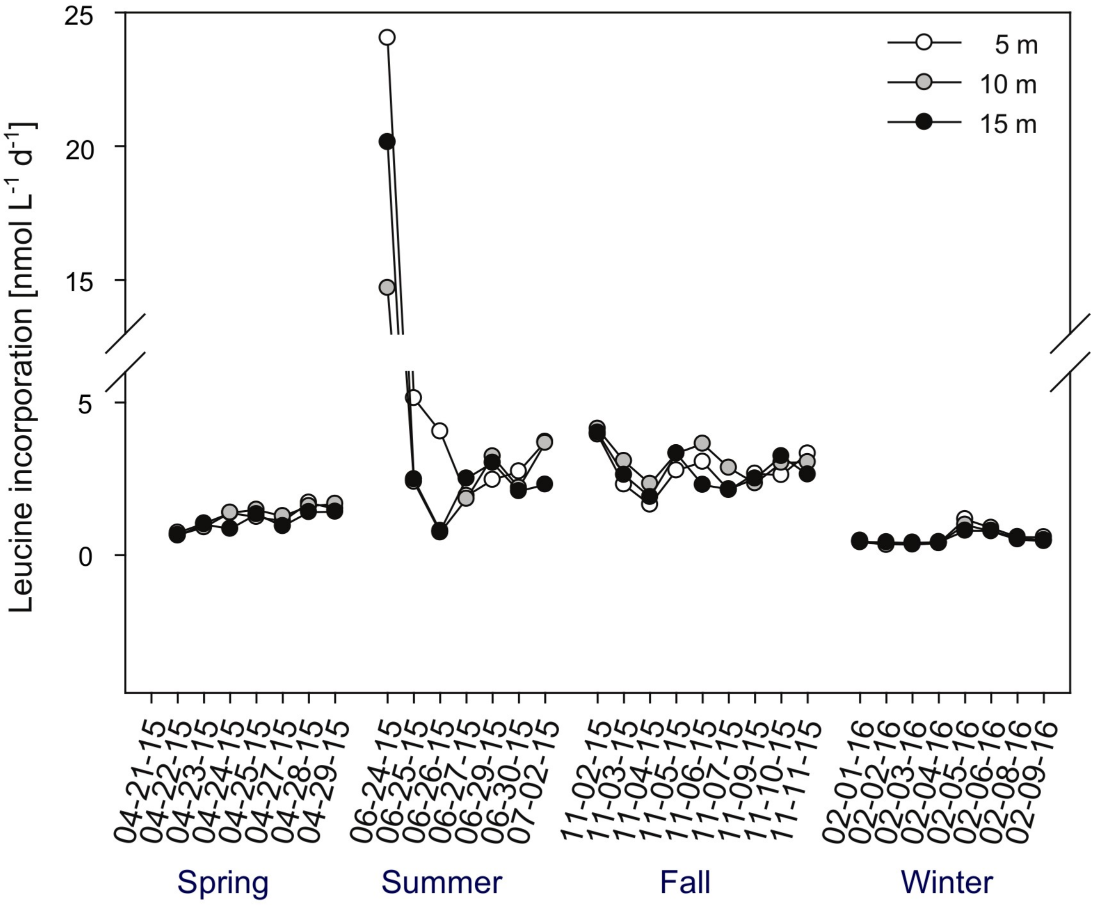

Figure S8. Leucine incorporation rates in the coastal northern Adriatic Sea.

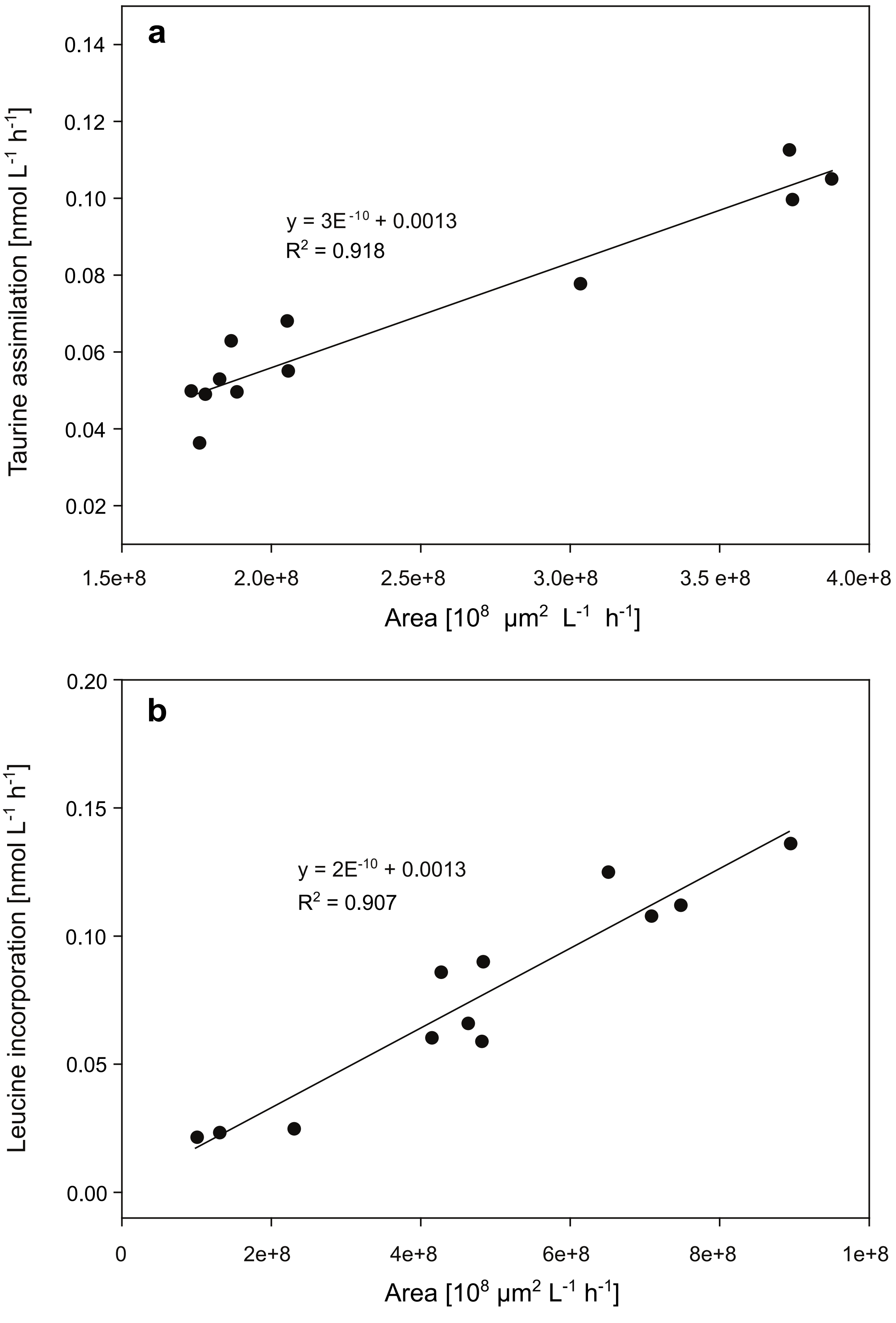

Figure S9. Relationship between total average silver grain area and bulk taurine assimilation (a) and bulk leucine incorporation (b) in the coastal northern Adriatic Sea.

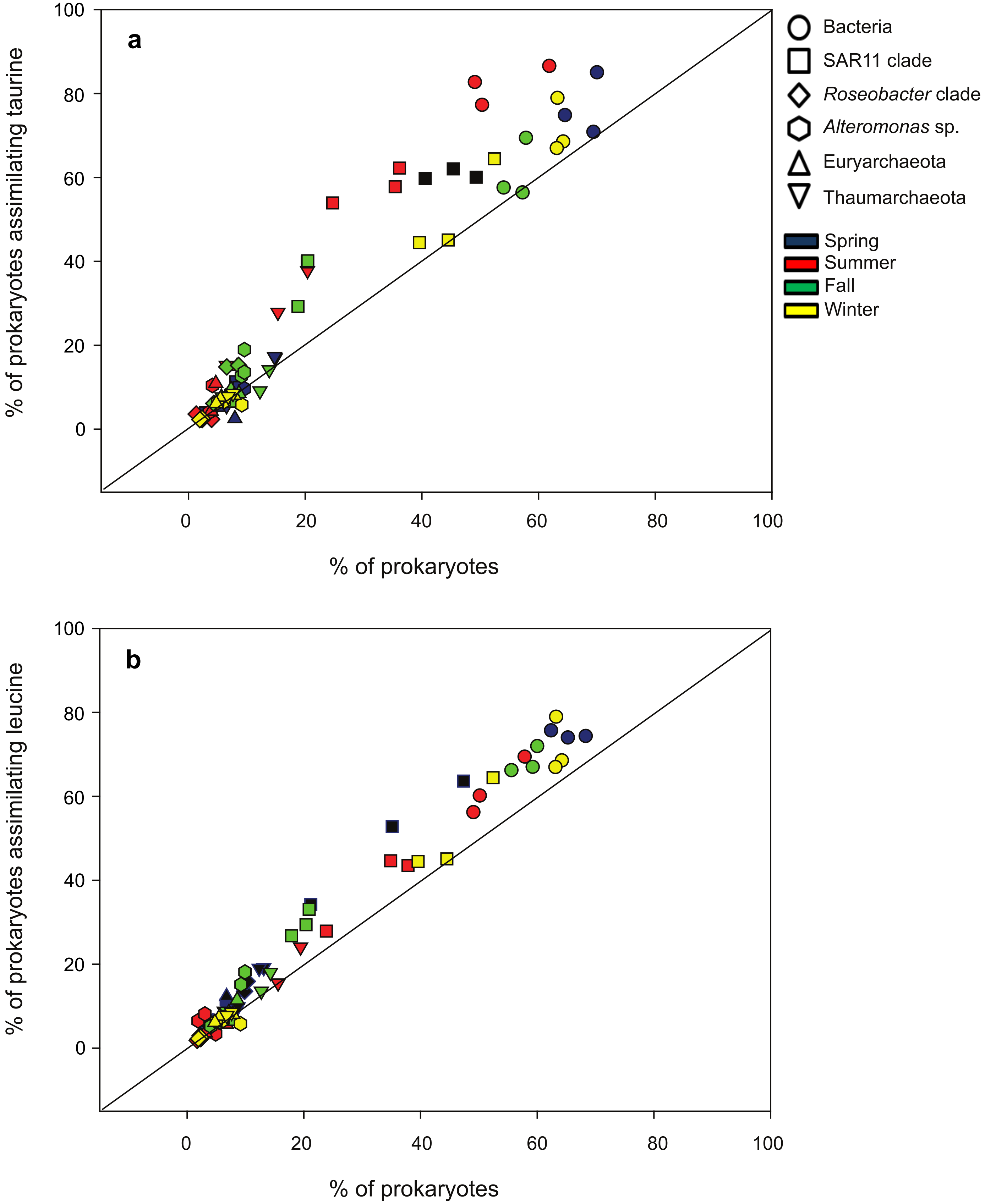

Figure S10. Contribution of prokaryotes assimilating taurine (a) and leucine (b) of the respective prokaryotic taxa to the taurine or leucine assimilating community versus their contribution to the abundance of prokaryotes in the coastal northern Adriatic Sea throughout the year. Colors indicate the sampling month and symbols indicate the taxa.

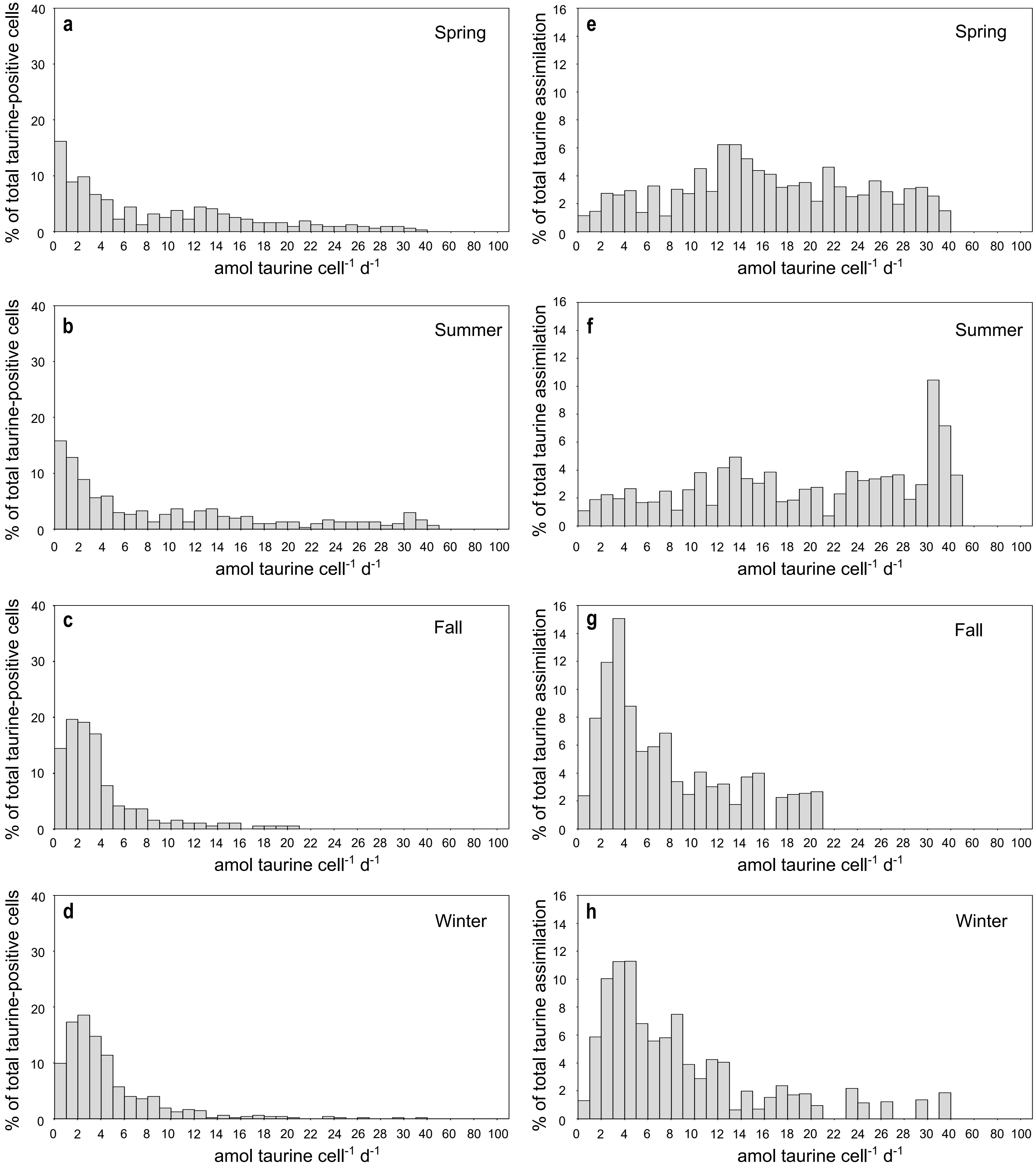

Figure S11. Distribution of cell-specific taurine assimilation by SAR11 cells expressed as percentage of the abundance (a-d) and as percentage of the total taurine assimilation (e-h) over a seasonal cycle.
