# Supplementary material for: Mesozooplankton taurine production and prokaryotic uptake in the northern Adriatic Sea
Source: Limnol Oceanogr. 2020 Jun 25;65(11):2730–47. doi: 10.1002/lno.11544 (PMC7891661; doi:10.1002/lno.11544)
Supplement: Supplementary file 2 — Table S1 Supporting information [file LNO-65-2730-s002.docx]

**Table S1a.** Zooplankton species composition during incubations to determine the taurine and dissolved free amino acid release over a seasonal cycle in the coastal northern Adriatic Sea (spring). Mean length and mean width measurements of the zooplankton are given in mm. Replicates (Repl.) are indicated by the numbers 1 to 3.

| **Date** | **Repl.** | ***Acartia* sp.** | | ***Centropagus* sp.** | | ***Calanus* sp.** | | ***Oithona* sp.** | | ***Oncaea* sp.** | | ***Temora stylifera*** | | **other Calanoida** | | **Harpacticoida** | | ***Penilia avirostris*** | | **unknown** | |
| --- | --- | --- | --- | --- | --- | --- | --- | --- | --- | --- | --- | --- | --- | --- | --- | --- | --- | --- | --- | --- | --- |
|  |  | **length** | **width** | **length** | **width** | **length** | **width** | **length** | **width** | **length** | **width** | **length** | **width** | **length** | **width** | **length** | **width** | **length** | **width** | **length** | **width** |
| **04/21/15** | **1** | 0.8 ± 0.2 | 0.3 ± 0.1 | 0.8 ± 0.1 | 0.3 ± 0.1 | 1.2 ± 0.2 | 0.4 ± 0.1 | - | - | - | - | - | - | 0.7 ± 0.1 | 0.3 ± 0.1 | - | - | - | - | - | - |
|  | **2** | 0.8 ± 0.1 | 0.3 ± 0.1 | 0.8 ± 0.1 | 0.3 ± 0.1 | 1.3 ± 0.5 | 0.4 ± 0.1 | - | - | - | - | - | - | 0.6 ± 0.1 | 0.3 ± 0.1 | - | - | - | - | - | - |
| **04/22/15** | **1** | 0.7 ± 0.1 | 0.2 ± 0.1 | 0.7 ± 0.2 | 0.3 ± 0.1 | - | - | - | - | - | - | - | - | - | - | - | - | - | - | - | - |
|  | **2** | 0.7 ± 0.1 | 0.2 ± 0.0 | 0.8 ± 0.1 | 0.3 ± 0.1 | - | - | - | - | - | - | - | - | - | - | - | - | - | - | - | - |
|  | **3** | 0.7 ± 0.1 | 0.2 ± 0.0 | 0.8 ± 0.2 | 0.3 ± 0.1 | - | - | - | - | - | - | - | - | - | - | - | - | - | - | - | - |
| **04/27/15** | **1** | 0.8 ± 0.2 | 0.3 ± 0.1 | 0.8 ± 0.2 | 0.3 ± 0.1 | - | - | - | - | - | - | - | - | - | - | - | - | - | - | - | - |
|  | **2** | 0.7 ± 0.2 | 0.2 ± 0.1 | 0.9 ± 0.2 | 0.3 ± 0.1 | - | - | - | - | - | - | - | - | - | - | - | - | - | - | - | - |
|  | **3** | 0.8 ± 0.1 | 0.3 ± 0.0 | 0.9 ± 0.1 | 0.3 ± 0.1 | - | - | - | - | - | - | - | - | - | - | - | - | - | - | - | - |
| **04/28/15** | **1** | 0.8 ± 0.2 | 0.3 ± 0.1 | 0.9 ± 0.2 | 0.3 ± 0.1 | - | - | - | - | - | - | - | - | - | - | - | - | - | - | - | - |
|  | **2** | 0.8 ± 0.2 | 0.2 ± 0.1 | 0.8 ± 0.3 | 0.3 ± 0.1 | - | - | - | - | - | - | - | - | - | - | - | - | - | - | - | - |
|  | **3** | 0.8 ± 0.2 | 0.3 ± 0.1 | 0.7 ± 0.1 | 0.3 ± 0.0 | - | - | - | - | - | - | - | - | - | - | - | - | - | - | - | - |

**Table S1b.** Zooplankton species composition during incubations to determine the taurine and dissolved free amino acid release over a seasonal cycle in the coastal northern Adriatic Sea (summer). Mean length and mean width measurements of the zooplankton are given in mm. Replicates (Repl.) are indicated by the numbers 1 to 3.

| **Date** | **Replicate** | ***Acartia* sp.** | | ***Centropagus* sp.** | | ***Calanus* sp.** | | ***Oithona* sp.** | | ***Oncaea* sp.** | | ***Temora stylifera*** | | **other Calanoida** | | **Harpacticoida** | | ***Penilia avirostris*** | | **unknown** | |
| --- | --- | --- | --- | --- | --- | --- | --- | --- | --- | --- | --- | --- | --- | --- | --- | --- | --- | --- | --- | --- | --- |
|  |  | **length** | **width** | **length** | **width** | **length** | **width** | **length** | **width** | **length** | **width** | **length** | **width** | **length** | **width** | **length** | **width** | **length** | **width** | **length** | **width** |
| **06/24/15** | **1** | 0.6 ± 0.2 | 0.3 ± 0.1 | 0.5 ± 0.2 | 0.2 ± 0.1 | - | - | - | - | - | - | - | - | - | - | - | - | 0.6 ± 0.1 | 0.2 ± 0.1 | 1.1 | 0.3 |
|  | **2** | 0.7 ± 0.1 | 0.3 ± 0.3 | 0.6 ± 0.1 | 0.2 ± 0.0 | - | - | - | - | - | - | - | - | - | - | - | - | 0.6 ± 0.1 | 0.3 ± 0.1 | - | - |
|  | **3** | 0.8 ± 0.2 | 0.3 ± 0.2 | 0.8 | 0.3 | - | - | - | - | - | - | - | - | - | - | - | - | 0.6 ± 0.1 | 0.3 ± 0.1 | - | - |
| **06/25/15** | **1** | 0.8 ± 0.1 | 0.3 ± 0.1 | 0.8 ± 0.2 | 0.3 ± 0.1 | - | - | - | - | - | - | - | - | - | - | - | - | 0.8 ± 0.1 | 0.5 ± 0.1 | - | - |
|  | **2** | 0.8 ± 0.2 | 0.3 ± 0.1 | 0.7 ± 0.2 | 0.3 ± 0.1 | - | - | - | - | - | - | - | - | - | - | - | - | 0.8 ± 0.1 | 0.1 ± 0.1 | - | - |
|  | **3** | 0.8 ± 0.1 | 0.3 ± 0.1 | 0.8 ± 0.3 | 0.3 ± 0.1 | - | - | - | - | - | - | - | - | - | - | - | - | 0.3 ± 0.1 | 0.8 ± 0.1 | - | - |
| **06/27/15** | **1** | 0.7 ± 0.2 | 0.3 ± 0.1 | 0.7 ± 0.2 | 0.3 ± 0.1 | - | - | - | - | - | - | - | - | - | - | - | - | 0.7 ± 0.1 | 0.3 ± 0.1 | - | - |
|  | **2** | 0.7 ± 0.2 | 0.2 ± 0.1 | 0.8 ± 0.2 | 0.3 ± 0.1 | - | - | - | - | - | - | - | - | - | - | - | - | 0.7 ± 0.1 | 0.3 ± 0.1 | - | - |
|  | **3** | 0.7 ± 0.1 | 0.3 ± 0.5 | 0.7 ± 0.1 | 0.3 ± 0.1 | - | - | - | - | - | - | - | - | - | - | - | - | 0.6 ± 0.1 | 0.3 ± 0.1 | 0.7 ± 0.1 | 0.3 ± 0.1 |
| **06/28/15** | **1** | 0.7 ± 0.1 | 0.2 ± 0.1 | 0.6 ± 0.1 | 0.3 ± 0.1 | - | - | - | - | - | - | - | - | - | - | - | - | 0.6 ± 0.1 | 0.4 ± 0.1 | - | - |
|  | **2** | 0.6 ± 0.1 | 0.2 ± 0.1 | 0.6 ± 0.2 | 0.3 ± 0.1 | - | - | - | - | - | - | - | - | - | - | - | - | 0.7 ± 0.1 | 0.3 ± 0.1 | - | - |
|  | **3** | 0.7 ± 0.2 | 0.3 ± 0.1 | 0.7 ± 0.2 | 0.3 ± 0.1 | - | - | - | - | - | - | - | - | - | - | - | - | 0.7 ± 0.1 | 0.3 ± 0.1 | - | - |

| **Date** | **Repl.** | ***Acartia* sp.** | | ***Centropagus* sp.** | | ***Calanus* sp.** | | ***Oithona* sp.** | | ***Oncaea* sp.** | | ***Temora stylifera*** | | **other Calanoida** | | **Harpacticoida** | | ***Penilia avirostris*** | | **unknown** | |
| --- | --- | --- | --- | --- | --- | --- | --- | --- | --- | --- | --- | --- | --- | --- | --- | --- | --- | --- | --- | --- | --- |
|  |  | **length** | **width** | **length** | **width** | **length** | **width** | **length** | **width** | **length** | **width** | **length** | **width** | **length** | **width** | **length** | **width** | **length** | **width** | **length** | **width** |
| **11/03/15** | **1** | - | - | - | - | 1.0 | 0.4 | 0.7 | 0.3 | 0.4 ± 0.0 | 0.2 ± 0.0 | - | - | - | - | 0.3 ± 0.0 | 0.2 ± 0.0 | - | - | - | - |
|  | **2** | 0.8 ± 0.1 | 0.3 ± 0.0 | - | - | 0.7 ± 0.3 | 0.2 ± 0.1 | 0.5 ± 0.1 | 0.2 ± 0.1 | 0.4 ± 0.1 | 0.2 ± 0.0 | - | - | - | - |  |  | - | - | - | - |
|  | **3** | - | - | - | - | 0.7 ± 0.1 | 0.3 ± 0.1 | 0.41 | 0.2 | 0.4 ± 0.1 | 0.2 ± 0.1 | - | - | - | - | 0.4 ± 0.1 | 0.2 ± 0.1 | - | - | - | - |
| **11/04/15** | **1** | 0.6 ± 0.1 | 0.2 ± 0.1 | 0.6 | 0.4 | 0.8 ± 0.2 | 0.3 ± 0.1 | 0.6 | 0.4 | 0.4 ± 0.1 | 0.2 ± 0.1 | 0.9 | 0.6 | 1.0 ± 0.4 | 0.4 ± 0.1 | 0.5 ± 0.2 | 0.2 ± 0.0 | - | - | - | - |
|  | **2** | 0.9 ± 0.1 | 0.3 ± 0.1 | 0.8 ± 0.4 | 0.3 ± 0.1 | 0.9 ± 0.3 | 0.3 ± 0.1 | 0.5 ± 0.0 | 0.2 ± 0.0 | 0.4 ± 0.1 | 0.2 ± 0.1 | - | - | 0.6 ± 0.0 | 0.3 ± 0.0 | 0.6 ± 0.2 | 0.2 ± 0.1 | - | - | - | - |
|  | **3** | 0.8 ± 0.1 | 0.2 ± 0.0 | - | - | 0.8 ± 0.3 | 0.3 ± 0.1 | 0.6 ± 0.1 | 0.2 ± 0.0 | 0.4 ± 0.1 | 0.2 ± 0.1 | 1.0 | 0.6 | - | - | 0.5 ± 0.1 | 0.2 ± 0.0 | - | - | - | - |
| **11/05/15** | **1** | 0.8 ± 0.1 | 0.3 ± 0.1 | 0.8 ± 0.2 | 0.3 ± 0.1 | - | - | 0.4 ± 0.1 | 0.2 ± 0.0 | 0.4 ± 0.1 | 0.2 ± 0.1 | 0.9 | 0.4 | 0.7 ± 0.2 | 0.3 ± 0.1 | 0.5 ± 0.1 | 0.2 ± 0.0 | - | - | - | - |
|  | **2** | 0.8 ± 0.1 | 0.3 ± 0.1 | 0.8 ± 0.1 | 0.3 ± 0.1 | - | - | 0.4 ± 0.1 | 0.2 ± 0.0 | 0.4 ± 0.1 | 0.2 ± 0.1 | - | - | 0.5 ± 00 | 0.2 ± 0.0 | 0.5 ± 0.2 | 0.2 ± 0.1 | - | - | - | - |
|  | **3** | 0.8 ± 0.2 | 0.2 ± 0.1 | 0.7 | 0.4 | - | - | 0.4 ± 0.1 | 0.2 ± 0.1 | 0.4 ± 0.1 | 0.2 ± 0.1 | - | - | 0.7 ± 0.1 | 0.3 ± 0.1 | 0.4 ± 0.0 | 0.2 ± 0.0 | - | - | - | - |
| **11/06/15** | **1** | 0.8 ± 0.2 | 0.3 ± 0.1 | 0.8 ± 0.2 | 0.3 ± 0.1 | 0.5 | 0.3 | 0.6 ± 0.4 | 0.3 ± 0.2 | 0.4 ± 0.1 | 0.2 ± 0.1 | - | - | 0.7 ± 0.1 | 0.2 ± 0.0 |  |  | - | - | - | - |
|  | **2** | 0.8 | 0.3 | - | - | 0.6 | 0.2 | 0.5 ± 0.2 | 0.2 ± 0.1 | 0.4 ± 0.1 | 0.2 ± 0.1 | 0.3 | - | 0.7 ± 0.2 | 0.3 ± 0.1 | 0.4 ± 0.0 | 0.2 ± 0.0 | - | - | - | - |
|  | **3** | 0.8 ± 0.1 | 0.3 ± 0.0 | - | - | - | - | 0.5 ± 0.2 | 0.2 ± 0.1 | 0.4 ± 0.2 | 0.2 ± 0.1 | - | - | 0.7 ± 0.2 | 0.3 ± 0.0 |  |  | - | - | - | - |
| **11/07/15** | **1** | 0.7 ± 0.2 | 0.3 ± 0.1 | 0.8 ± 0.2 | 0.4 ± 0.1 | 0.8 ± 0.7 | - | 0.5 ± 0.1 | 0.2 ± 0.2 | 0.4 ± 0.2 | 0.2 ± 0.2 | - | - | 0.7 ± 0.3 | 0.3 ± 0.3 | 0.5 ± 0.2 | 0.2 ± 0.1 | - | - | - | - |
|  | **2** | 0.9 ± 0.4 | 0.3 ± 0.1 | 0.6 | 0.2 | 0.9 ± 0.7 | 0.4 ± 0.2 | 0.7 ± 0.7 | 0.2 ± 0.3 | 0.4 ± 0.2 | 0.2 ± 0.2 | - | - | 0.7 ± 0.3 | 0.2 ± 0.1 | 0.3 ± 0.0 | 0.1 ± 0.0 | - | - | - | - |
|  | **3** | 0.9 ± 0.4 | 0.3 ± 0.1 | 0.8 ± 0.3 | 0.4 ± 0.1 | 0.8 ± 0.1 | 0.3 ± 0.1 | 0.52 ± 0 | 0.2 ± 0.1 | 0.4 ± 0.2 | 0.2 ± 0.2 | - | - | 0.7 ± 0.3 | 0.2 ± 0.1 | 0.6 ± 0.3 | 0.3 ± 0.0 | - | - | - | - |
| **11/10/15** | **1** | 0.9 ± 0.5 | 0.3 ± 0.1 | 1.0 ± 0.2 | 0.4 ± 0.1 | 0.9 ± 0.5 | 0.4 ± 0.1 | 0.7 ± 0.2 | 0.2 ± 0.1 | 0.4 ± 0.2 | 0.2 ± 0.2 | 0.9 ± 0.3 | 0.4 ± 0.1 | 0.7 ± 0.3 | 0.4 ± 0.2 | 0.4 | 0.2 | - | - | - | - |
|  | **2** | 0.9 ± 0.5 | 0.3 ± 0.1 | 0.9 ± 0.1 | 0.3 ± 0.0 | 1.3 | 0.4 | 0.7 ± 0.1 | 0.3 ± 0.0 | 0.4 ± 0.2 | 0.2 ± 0.2 | 0.9 ± 0.2 | 0.5 ± 0.1 | 0.7 ± 0.3 | 0.4 ± 0.2 | 0.6 | 0.3 | - | - | - | - |
|  | **3** | 0.9 ± 0.5 | 0.3 ± 0.1 | 0.9 ± 0.3 | 0.4 ± 0.1 | - | - | 0.7 ± 0.1 | 0.2 ± 0.0 | 0.4 ± 0.21 | 0.2 ± 0.2 | 0.4 | 0.2 | 0.7 ± 0.3 | 0.4 ± 0.2 | 0.7 | 0.3 | - | - | - | - |

**Table S1c.** Zooplankton species composition during incubations to determine the taurine and dissolved free amino acid release over a seasonal cycle in the coastal northern Adriatic Sea (fall). Mean length and mean width measurements of the zooplankton are given in mm. Replicates (Repl.) are indicated by the numbers 1 to 3.

| **Date** | **Repl.** | ***Acartia* sp.** | | ***Centropagus* sp.** | | ***Calanus* sp.** | | ***Oithona* sp.** | | ***Oncaea* sp.** | | ***Temora stylifera*** | | **other Calanoida** | | **Harpacticoida** | | ***Penilia avirostris*** | | **unknown** | |
| --- | --- | --- | --- | --- | --- | --- | --- | --- | --- | --- | --- | --- | --- | --- | --- | --- | --- | --- | --- | --- | --- |
|  |  | **length** | **width** | **length** | **width** | **length** | **width** | **length** | **width** | **length** | **width** | **length** | **width** | **length** | **width** | **length** | **width** | **length** | **width** | **length** | **width** |
| **02/02/16** | **1** | 0.6 ± 0.2 | 0.2 ± 0.1 | 0.7 ± 0.3 | 0.3 ± 0.1 | - | - | 0.4 ± 0.1 | 0.2 ± 0.1 | 0.4 ± 0.1 | 0.2 ± 0.0 | 0.3 ± 0.0 | 0.2 ± 0.0 | 0.5 ± 0.2 | 0.2 ± 0.1 | 0.3 ± 0.1 | 0.1 ± 0.0 | - | - | 0.3 | 0.1 |
|  | **2** | 0.8 ± 0.3 | 0.3 ± 0.1 | 0.7 ± 0.3 | 0.2 ± 0.1 | 0.7 ± 0.2 | 0.3 ± 0.1 | 0.4 ± 0.1 | 0.2 ± 0.0 | 0.4 ± 0.1 | 0.2 ± 0.0 | 0.6 | 0.3 | 0.4 ± 0.1 | 0.2 ± 0.1 | 0.4 ± 0.1 | 0.1 ± 0.0 | - | - | 0.3 ± 0.1 | 0.1 ± 0.0 |
|  | **3** | 0.8 ± 0.4 | 0.3 ± 0.1 | 0.9 | 0.3 | 0.9 ± 0.4 | 0.3 ± 0.1 | 0.4 ± 0.1 | 0.2 ± 0.0 | 0.3 ± 0.0 | 0.2 ± 0.0 | 0.5 ± 0.4 | 0.2 ± 0.1 | 0.5 ± 0.2 | 0.2 ± 0.1 | 0.3 ± 0.1 | 0.1 ± 0.0 | - | - | 0.3 | 0.2 |
| **02/03/16** | **1** | 0.6 ± 0.2 | 0.2 ± 0.1 | 1.0 ± 0.3 | 0.4 ± 0.1 | 0.8 ± 0.4 | 0.3 ± 0.1 | 0.4 ± 0.1 | 0.2 ± 0.0 | 0.3 ± 0.1 | 0.2 ± 0.1 | 0.3 | 0.1 | 0.5 ± 0.1 | 0.2 ± 0.1 | 0.3 ± 0.1 | 0.2 ± 0.1 | - | - | **-** | **-** |
|  | **2** | 0.7 ± 0.2 | 0.3 ± 0.1 | - | - | 0.8 ± 0.3 | 0.3 ± 0.1 | 0.4 ± 0.1 | 0.1 ± 0.0 | 0.4 ± 0.1 | 0.2 ± 0.1 | 0.4 ± 0.1 | 0.2 ± 0.0 | 0.5 ± 0.2 | 0.2 ± 0.1 | 0.4 ± 0.2 | 0.1 ± 0.1 | - | - | **-** | **-** |
|  | **3** | 0.9 ± 0.2 | 0.3 ± 0.1 | 0.7 | 0.2 | 0.7 ± 0.0 | 0.3 ± 0.0 | 0.4 ± 0.1 | 0.2 ± 0.0 | 0.4 ± 0.1 | 0.2 ± 0.0 | - | - | 0.5 ± 0.2 | 0.2 ± 0.1 | 0.3 ± 0.1 | 0.1 ± 0.1 | - | - | 0.3 | 0.1 |
| **02/05/16** | **1** | 0.8 ± 0.2 | 0.3 ± 0.1 | - | - | - | - | 0.5 | 0.2 | 0.5 ± 0.1 | 0.4 ± 0.0 | - | - | 0.6 ± 0.2 | 0.2 ± 0.1 | - | - | - | - | 0.3 | 2.0 |
|  | **2** | 0.8 ± 0.2 | 0.3 ± 0.1 | 0.5 | 0.3 | 0.8 ± 0.2 | 0.3 ± 0.1 | 0.5 ± 0.1 | 0.2 ± 0.0 | 0.5 | 0.2 | - | - | 0.6 ± 0.2 | 0.2 ± 0.1 | 0.5 ± 0.1 | 0.2 ± 0.1 | - | - | - | - |
|  | **3** | 0.9 ± 0.1 | 0.3 ± 0.1 | - | - | - | - | 0.5 ± 0.1 | 0.2 ± 0.1 | - | - | - | - | 0.6 ± 0.2 | 0.2 ± 0.1 | - | - | - | - | - | - |
| **02/09/16** | **1** | 0.9 ± 0.2 | 0.3 ± 0.1 | 0.8 ± 0.2 | 0.3 ± 0.1 | 0.9 ± 0.6 | 0.3 ± 0.2 | 0.4 ± 0.1 | 0.2 ± 0.0 | 0.4 ± 0.1 | 0.2 ± 0.0 | 0.6 ± 0.2 | 0.4 ± 0.1 | 0.7 ± 0.2 | 0.3 ± 0.1 | 0.4 ± 0.1 | 0.2 ± 0.0 | - | - | 0.6 ± 0.2 | 0.2 ± 0.1 |
|  | **2** | 0.9 ± 0.1 | 0.3 ± 0.1 | 1.2 | 0.4 | 0.9 ± 0.2 | 0.3 ± 0.0 | 0.7 ± 0.0 | 0.3 ± 0.0 | 0.4 ± 0.0 | 0.2 ± 0.0 | 0.7 ± 0.0 | 0.4 ± 0.1 | 0.7 ± 0.2 | 0.3 ± 0.1 | 0.3 | 0.2 | - | - | 0.7 ± 0.1 | 0.3 ± 0.1 |
|  | **3** | 0.9 ± 0.1 | 0.3 ± 0.1 | 0.8 ± 0.2 | 0.4 ± 0.1 | 0.9 ± 0.2 | 0.4 ± 0.1 | 0.5 ± 0.1 | 0.2 ± 0.0 | 0.4 | 0.3 | - | - | 0.7 ± 0.2 | 0.3 ± 0.1 | 0.8 | 0.3 | - | - | - | - |

**Table S1d.** Zooplankton species composition during incubations to determine the taurine and dissolved free amino acid release over a seasonal cycle in the coastal northern Adriatic Sea (winter). Mean length and mean width measurements of the zooplankton are given in mm. Replicates (Repl.) are indicated by the numbers 1 to 3.

**Table S2a.** Number (N) of individuals used during the zooplankton incubations to measure taurine and dissolved free amino acids release rates during the spring 2015.

| **Date** | **Repl.** | ***Acartia* sp.** | ***Centropagus* sp.** | ***Calanus* sp.** | ***Oithona* sp.** | ***Oncaea* sp.** | ***Temora stylifera*** | **other Calanoida** | **Harpacticoida** | ***Penilia avirostris*** | **unknown** | **Total N** |
| --- | --- | --- | --- | --- | --- | --- | --- | --- | --- | --- | --- | --- |
| **04/21/15** | **1** | 12 | 14 | 3 | - | - | - | 15 | - | - | - | 44 |
|  | **2** | 11 | 28 | 3 | - | - | - | 15 | - | - | - | 57 |
| **04/22/15** | **1** | 55 | 15 | - | - | - | - | - | - | - | - | 70 |
|  | **2** | 49 | 7 | - | - | - | - | - | - | - | - | 56 |
|  | **3** | 35 | 28 | - | - | - | - | - | - | - | - | 63 |
| **04/27/15** | **1** | 86 | 12 | - | - | - | - | - | - | - | - | 98 |
|  | **2** | 64 | 15 | - | - | - | - | - | - | - | - | 79 |
|  | **3** | 56 | 5 | - | - | - | - | - | - | - | - | 61 |
| **04/28/15** | **1** | 72 | 7 | - | - | - | - | - | - | - | - | 79 |
|  | **2** | 59 | 13 | - | - | - | - | - | - | - | - | 72 |
|  | **3** | 71 | 4 | - | - | - | - | - | - | - | - | 75 |

| **Date** | **Repl.** | ***Acartia* sp.** | ***Centropagus* sp.** | ***Calanus* sp.** | ***Oithona* sp.** | ***Oncaea* sp.** | ***Temora stylifera*** | **other Calanoida** | **Harpacticoida** | ***Penilia avirostris*** | **unknown** | **Total N** |
| --- | --- | --- | --- | --- | --- | --- | --- | --- | --- | --- | --- | --- |
| **06/24/15** | **1** | 71 | 3 | - | - | - | - | - | - | 47 | 1 | 122 |
|  | **2** | 73 | 4 | - | - | - | - | - | - | 42 | - | 119 |
|  | **3** | 61 | 1 | - | - | - | - | - | - | 54 | - | 116 |
| **06/25/15** | **1** | 63 | 12 | - | - | - | - | - | - | 17 | - | 92 |
|  | **2** | 61 | 13 | - | - | - | - | - | - | 9 | - | 83 |
|  | **3** | 56 | 20 | - | - | - | - | - | - | 17 | - | 93 |
| **06/27/15** | **1** | 57 | 9 | - | - | - | - | - | - | 31 | - | 97 |
|  | **2** | 46 | 3 | - | - | - | - | - | - | 54 | - | 103 |
|  | **3** | 30 | 28 | - | - | - | - | - | - | 29 | 12 | 99 |
| **06/28/15** | **1** | 42 | 16 | - | - | - | - | - | - | 45 | - | 103 |
|  | **2** | 33 | 5 | - | - | - | - | - | - | 65 | - | 103 |
|  | **3** | 62 | 6 | - | - | - | - | - | - | 39 | - | 107 |

**Table S2b.** Number (N) of individuals used during the zooplankton incubations to measure taurine and dissolved free amino acids release rates during the summer.

| **Date** | **Repl.** | ***Acartia* sp.** | ***Centropagus* sp.** | ***Calanus* sp.** | ***Oithona* sp.** | ***Oncaea* sp.** | ***Temora stylifera*** | **other Calanoida** | **Harpacticoida** | ***Penilia avirostris*** | **unknown** | **Total N** |
| --- | --- | --- | --- | --- | --- | --- | --- | --- | --- | --- | --- | --- |
| **11/03/15** | **1** | - | - | 1 | 1 | 64 | - | - | 3 | - | - | 69 |
|  | **2** | 3 | - | 4 | 20 | 54 | - | - | - | - | - | 81 |
|  | **3** | - | - | 3 | 1 | 58 | - | - | 20 | - | - | 82 |
| **11/04/15** | **1** | 3 | 1 | 13 | 1 | 28 | 1 | 4 | 4 | - | - | 55 |
|  | **2** | 20 | - | 6 | 2 | 14 |  | 2 | 3 | - | - | 47 |
|  | **3** | 5 | - | 21 | 6 | 13 | 1 | - | 8 | - | - | 54 |
| **11/05/15** | **1** | 6 | 5 | - | 9 | 58 | 1 | 9 | 10 | - | - | 98 |
|  | **2** | 4 | 2 | - | 8 | 66 | - | 4 | 14 | - | - | 98 |
|  | **3** | 9 | 1 | - | 13 | 52 | - | 8 | 10 | - | - | 93 |
| **11/06/15** | **1** | 10 | 5 | 1 | 5 | 53 | - | 2 |  | - | - | 76 |
|  | **2** | 1 | - | 1 | 3 | 63 | 1 | 3 | 4 | - | - | 76 |
|  | **3** | 3 | - | - | 4 | 55 | - | 7 |  | - | - | 69 |
| **11/07/15** | **1** | 13 | 5 | 15 | 19 | 36 | - | 15 | 3 | - | - | 106 |
|  | **2** | 14 | 1 | 17 | 12 | 50 | - | 13 | 4 | - | - | 111 |
|  | **3** | 6 | 3 | 5 | 6 | 57 | - | 24 | 2 | - | - | 103 |
| **11/10/15** | **1** | 37 | 7 | 3 | 5 | 1 | 2 | 4 | 1 | - | - | 60 |
|  | **2** | 39 | 2 | 1 | 11 | - | 2 | 11 | 1 | - | - | 67 |
|  | **3** | 27 | 14 | - | 8 | - | 1 | 11 | 1 | - | - | 62 |

**Table S2c.** Number (N) of individuals used during the zooplankton incubations to measure taurine and dissolved free amino acids release rates during the fall.

| **Date** | **Repl.** | ***Acartia* sp.** | ***Centropagus* sp.** | ***Calanus* sp.** | ***Oithona* sp.** | ***Oncaea* sp.** | ***Temora stylifera*** | **other Calanoida** | **Harpacticoida** | ***Penilia avirostris*** | **unknown** | **Total N** |
| --- | --- | --- | --- | --- | --- | --- | --- | --- | --- | --- | --- | --- |
| **02/02/16** | **1** | 37 | 12 | - | 36 | 15 | 2 | 15 | 8 | - | 1 | 126 |
|  | **2** | 8 | 3 | 3 | 31 | 9 | 1 | 46 | 9 | - | 4 | 114 |
|  | **3** | 4 | 1 | 3 | 18 | 7 | 5 | 63 | 5 | - | 1 | 107 |
| **02/03/16** | **1** | 11 | 6 | 2 | 9 | 19 | 1 | 39 | 6 | - | - | 93 |
|  | **2** | 7 | - | 4 | 9 | 15 | 2 | 33 | 6 | - | - | 76 |
|  | **3** | 66 | 1 | 2 | 12 | 6 | - | 24 | 2 | - | 1 | 114 |
| **02/05/16** | **1** | 58 | - | - | 1 | 2 | - | 14 | - | - | 1 | 76 |
|  | **2** | 16 | 1 | 4 | 12 | 1 | - | 29 | 3 | - | - | 66 |
|  | **3** | 86 | - | - | 4 | - | - | 35 | - | - | - | 125 |
| **02/09/16** | **1** | 17 | 7 | 2 | 8 | 7 | 2 | 47 | 2 | - | 9 | 101 |
|  | **2** | 18 | 1 | 8 | 2 | 2 | 3 | 70 | 1 | - | 4 | 109 |
|  | **3** | 28 | 8 | 7 | 4 | 1 | - | 64 | 1 | - |  | 113 |

**Table S2d.** Number (N) of individuals used during the zooplankton incubations to measure taurine and dissolved free amino acids release rates during the winter.

| **Date** | **Depth** | **Time** | **T** | **R.** | **Unit** | **Asp** | **Glu** | **Asn** | **Ser** | **Gln** | **His** | **Gly** | **Arg** | **Thr** | **Ala** | **Tau** | **Gaba** | **Tyr** | **Met/Val** | **Try** | **Phe** | **Ile** | **Leu** | **Lys** | **DFAA** | **Tau/DFAA** | **Leu/DFAA %** |
| --- | --- | --- | --- | --- | --- | --- | --- | --- | --- | --- | --- | --- | --- | --- | --- | --- | --- | --- | --- | --- | --- | --- | --- | --- | --- | --- | --- |
| **04/21/15** | **22** | **10:00** | **14.8** | **1** | **nmol indivdual ^-1^d^-1^ x 10^2^** | 185.4 | 191.6 | - | 159.7 | - | - | 278.7 | 55.6 | - | 70.0 | 143.8 | - | - | - | - | - | - | - | - | - | - | - |
|  |  |  |  |  | **µmol g C^-1^ d^-1^** | 530.6 | 548.4 | - | 457.0 | - | - | 797.7 | 159.0 | - | 200.4 | 411.5 | - | - | - | - | - | - | - | - | - | - | - |
|  |  |  |  | **2** | **nmol indivdual ^-1^d^-1^ x 10^2^** | - | - | - | - | - | - | 159.6 | 31.5 | 96.7 | 34.4 | 56.3 | - | - | - | - | - | - | - | - | 194.0 | 0.29 | - |
|  |  |  |  |  | **µmol g C^-1^ d^-1^** | - | - | - | - | - | - | 489.3 | 96.6 | 296.5 | 105.5 | 172.6 | - | - | - | - | - | - | - | - | 594.9 | 0.29 | - |
| **04/22/15** | **22** | **10:00** | **14.7** | **1** | **nmol indivdual ^-1^d^-1^ x 10^2^** | - | - | - | - | - | - | 92.2 | - | 19.5 | - | 21.8 | - | - | - | - | - | - | - | - | 111.7 | 0.20 | - |
|  |  |  |  |  | **µmol g C^-1^ d^-1^** | - | - | - | - | - | - | 465.4 | - | 98.6 | - | 110.3 | - | - | - | - | - | - | - | - | 564.0 | 0.20 | - |
|  |  |  |  | **2** | **nmol indivdual ^-1^d^-1^ x 10^2^** | - | - | - | - | 0.2 | - | 86.4 | - | 7.5 | - | 6.5 | - | - | - | - | - | - | - | - | 94.1 | 0.07 | - |
|  |  |  |  |  | **µmol g C^-1^ d^-1^** | - | - | - | - | 1.0 | - | 466.2 | - | 40.4 | - | 35.3 | - | - | - | - | - | - | - | - | 507.7 | 0.07 | - |
|  |  |  |  | **3** | **nmol indivdual ^-1^d^-1^ x 10^2^** | - | - | - | - | - | - | 15.6 | - | - | - | 5.2 | - | - | - | - | - | - | - | - | 15.6 | 0.33 | - |
|  |  |  |  |  | **µmol g C^-1^ d^-1^** | - | - | - | - | - | - | 55.1 | - | - | - | 18.4 | - | - | - | - | - | - | - | - | 55.1 | 0.33 | - |
| **04/27/15** | **21m** | **10:00** | **15.3** | **1** | **nmol indivdual ^-1^d^-1^ x 10^2^** | - | 15.2 | - | 157.1 | - | - | 154.5 | 72.5 | 49.7 | 6.5 | 71.7 | - | - | - | - | - | - | - | 12.5 | 225.9 | 0.32 | - |
|  |  |  |  |  | **µmol g C^-1^ d^-1^** | - | 53.7 | - | 555.5 | - | - | 546.3 | 256.5 | 175.8 | 23.0 | 253.4 | - | - | - | - | - | - | - | 44.3 | 798.7 | 0.32 | - |
|  |  |  |  | **2** | **nmol indivdual ^-1^d^-1^ x 10^2^** | - | - | - | - | - | - | 448.3 | - | 121.8 | 36.9 | 164.1 | - | - | - | - | - | - | - | - | 607.1 | 0.27 | - |
|  |  |  |  |  | **µmol g C^-1^ d^-1^** | - | - | - | - | - | - | 1844.3 | - | 501.0 | 151.8 | 675.2 | - | - | - | - | - | - | - | - | 2497.2 | 0.27 | - |
|  |  |  |  | **3** | **nmol indivdual ^-1^d^-1^ x 10^2^** | - | - | - | 122.9 | - | - | 222.6 | 396.1 | 50.4 | 61.0 | 50.3 | - | - | - | - | - | - | 12.1 | - | 852.9 | - | 1.42 |
|  |  |  |  |  | **µmol g C^-1^ d^-1^** | - | - | - | 522.2 | - | - | 946.0 | 1683.0 | 214.2 | 259.0 | 213.9 | - | - | - | - | - | - | 51.6 | - | 3624.5 | - | 1.42 |
| **04/28/15** | **24** | **10:00** | **15.2** | **1** | **nmol indivdual ^-1^d^-1^ x 10^2^** | - | - | - | - | - | - | 115.2 | 22.7 | 69.8 | 24.8 | 40.6 | - | - | - | - | - | - | - | - | 140.0 | 0.29 | - |
|  |  |  |  |  | **µmol g C^-1^ d^-1^** | - | - | - | - | - | - | 457.4 | 90.3 | 277.1 | 98.6 | 161.3 | - | - | - | - | - | - | - | - | 556.0 | 0.29 | - |
|  |  |  |  | **2** | **nmol indivdual ^-1^d^-1^ x 10^2^** | - | - | - | - | - | - | 24.0 | - | 5.4 | - | 3.4 | - | - | - | - | - | - | - | - | 29.4 | 0.11 | - |
|  |  |  |  |  | **µmol g C^-1^ d^-1^** | - | - | - | - | - | - | 95.2 | - | 21.4 | - | 13.4 | - | - | - | - | - | - | - | - | 116.6 | 0.11 | - |
|  |  |  |  | **3** | **nmol indivdual ^-1^d^-1^ x 10^2^** | - | - | - | - | - | - | 41.7 | - | 11.7 | - | 6.2 | - | - | - | - | - | - | - | - | 53.4 | 0.12 | - |
|  |  |  |  |  | **µmol g C^-1^ d^-1^** | - | - | - | - | - | - | 152.9 | - | 42.8 | - | 22.9 | - | - | - | - | - | - | - | - | 195.6 | 0.12 | - |

**Table S3a.** Dissolved free amino acid release rates (nmol individual^-1^ d^-1^ x 10^2^ and µmol g C^-1^ d^-1^) by zooplankton communities during the spring (2015) in the coastal northern Adriatic Sea. The sum of DFAA (nmol individual^-1^ d^-1^ x 10^2^ and µmol g C^-1^ d^-1^), the ratios taurine/DFAA and the contribution of leucine to the total DFAA (%) are indicated. The green colored DFAA release rates did not increase significantly (*p* > 0.05, *R*^2^ < 0.6), as shown in Table S4. Replicates (R.) are indicated by numbers 1 to 3. The Tow depth (m), the tow time and temperature (T) are also indicated. Abbreviation for the amino acids are given in Table S3c.

| **Date** | **Depth** | **Time** | **T** | **R** | **Unit** | **Asp** | **Glu** | **Asn** | **Ser** | **Gln** | **His** | **Gly** | **Arg** | **Thr** | **Ala** | **Tau** | **Gaba** | **Tyr** | **Met/Val** | **Try** | **Phe** | **Ile** | **Leu** | **Lys** | **DFAA** | **Tau/DFAA** | **Leu/DFAA %** |
| --- | --- | --- | --- | --- | --- | --- | --- | --- | --- | --- | --- | --- | --- | --- | --- | --- | --- | --- | --- | --- | --- | --- | --- | --- | --- | --- | --- |
| **06/24/15** | **=** | **10:45** | **24.0** | **1** | **nmol indivdual ^-1^d^-1^ x 10^2^** | - | - | - | 21.5 | - | - | 28.1 | - | - | 17.6 | 63.6 | - | - | - | - | - | - | - | - | 67.2 | 0.95 | - |
|  |  |  |  |  | **µmol g C^-1^ d^-1^** | - | - | - | 46.5 | - | - | 60.9 | - | - | 1.6 | 137.8 | - | - | - | - | - | - | - | - | 109.0 | 1.26 | - |
|  |  |  |  | **2** | **nmol indivdual ^-1^d^-1^ x 10^2^** | 34.4 | 41.2 | - | 16.8 | - | - | 189.2 | 16.9 | 64.4 | 50.1 | 33.6 | - | - | 34.9 | - | 27.6 | - | 41.4 | - | 270.4 | 0.12 | - |
|  |  |  |  |  | **µmol g C^-1^ d^-1^** | 81.3 | 97.3 | - | 39.7 | - | - | 447.2 | 39.9 | 152.2 | 118.4 | 79.4 | - | - | 82.6 | - | 65.3 | - | 97.9 | - | 639.3 | 0.12 | - |
|  |  |  |  | **3** | **nmol indivdual ^-1^d^-1^ x 10^2^** | - | 49.6 | - | 8.4 | 3.2 | 25.9 | 342.6 | 28.8 | 114.8 | 54.4 | 58.7 | - | 19.9 | - | - | - | - | 20.9 | - | 648.7 | 0.09 | 3.22 |
|  |  |  |  |  | **µmol g C^-1^ d^-1^** | - | 189.9 | - | 32.1 | 12.2 | 99.2 | 1310.2 | 110.1 | 439.1 | 208.1 | 224.6 | - | 3.2 | - | - | - | - | 79.9 | - | 2480.8 | 0.09 | 3.22 |
| **06/25/15** | **20** | **10:30** | **24.0** | **1** | **nmol indivdual ^-1^d^-1^ x 10^2^** | - | - | - | - | - | 27.9 | 205.0 | - | 63.7 | - | 34.2 | - | 12.5 | 209.8 | 23.8 | - | - | 17.6 | 7.5 | 360.9 | 0.10 | 5.17 |
|  |  |  |  |  | **µmol g C^-1^ d^-1^** | - | - | - | - | - | 74.5 | 546.2 | - | 169.7 | - | 91.2 | - | 33.3 | 559.0 | 63.4 | - | - | 47.0 | 19.9 | 961.8 | 0.10 | 5.17 |
|  |  |  |  | **2** | **nmol indivdual ^-1^d^-1^ x 10^2^** | - | - | - | - | - | - | 351.2 | - | 89.1 | 0.0 | 56.3 | - | - | - | - | - | - | - | - | 440.2 | 0.13 | - |
|  |  |  |  |  | **µmol g C^-1^ d^-1^** | - | - | - | - | - | - | 989.3 | - | 250.9 | 221.0 | 158.6 | - | - | - | - | - | - | - | - | 1240.2 | 0.13 | - |
|  |  |  |  | **3** | **nmol indivdual ^-1^d^-1^ x 10^2^** | 152.9 | 289.9 | 61.1 | 169.0 | 11.8 | 114.6 | 198.3 | 94.3 | 185.0 | 138.6 | 124.2 | - | - | - | - | 34.6 | 34.8 | 48.9 | - | 383.3 | 0.32 | - |
|  |  |  |  |  | **µmol g C^-1^ d^-1^** | 350.5 | 664.8 | 140.1 | 387.5 | 27.1 | 262.8 | 454.8 | 216.3 | 424.2 | 317.8 | 284.9 | - | - | - | - | 79.3 | 79.8 | 112.2 | - | 879.0 | 0.32 | - |
| **06/27/15** | **18** | **10:30** | **24.0** | **1** | **nmol indivdual ^-1^d^-1^ x 10^2^** | - | - | - | - | - | - | 310.3 | - | - | 62.5 | 105.9 | - | - | - | - | - | - | - | - | 372.9 | - | - |
|  |  |  |  |  | **µmol g C^-1^ d^-1^** | - | - | - | - | - | - | 716.8 | - | - | 144.5 | 244.7 | - | - | - | - | - | - | - | - | 861.3 | - | - |
|  |  |  |  | **2** | **nmol indivdual ^-1^d^-1^ x 10^2^** | 13.7 | 63.2 | - | 7.6 | 3.5 | 16.1 | 247.1 | 11.2 | 91.6 | 59.2 | 84.2 | - | 27.6 | 28.4 | 15.8 | 25.6 | 23.2 | 33.9 | 25.9 | 593.3 | 0.14 | 5.72 |
|  |  |  |  |  | **µmol g C^-1^ d^-1^** | 55.5 | 256.7 | - | 30.9 | 14.0 | 65.2 | 1003.2 | 45.6 | 372.2 | 240.2 | 342.1 | - | 112.0 | 115.5 | 64.1 | 104.1 | 94.1 | 137.7 | 105.1 | 2409.3 | 0.14 | 5.72 |
|  |  |  |  | **3** | **nmol indivdual ^-1^d^-1^ x 10^2^** | 37.7 | 167.4 | - | 48.3 | - | 378.0 | 99.5 | 153.0 | 170.1 | 471.6 | 183.7 | - | 3.6 | 223.1 | 42.0 | 115.0 | 217.5 | 300.1 | 59.5 | 3.6 | - | - |
|  |  |  |  |  | **µmol g C^-1^ d^-1^** | 75.5 | 334.9 | - | 96.6 | - | 756.0 | 198.9 | 306.0 | 340.1 | 943.3 | 367.4 | - | 7.2 | 446.2 | 84.0 | 230.0 | 435.1 | 600.2 | 119.1 | 7.2 | - | - |
| **06/28/15** | **26** | **10:00** | **24.0** | **1** | **nmol indivdual ^-1^d^-1^ x 10^2^** | 130.3 | 91.3 | 22.3 | 222.8 | 2.1 | 51.8 | 128.2 | 99.2 | 53.5 | 142.2 | 106.8 | - | - | - | - | - | - | - | - | 53.9 | - | - |
|  |  |  |  |  | **µmol g C^-1^ d^-1^** | 297.9 | 208.8 | 51.0 | 509.2 | 4.9 | 118.4 | 293.0 | 226.8 | 122.2 | 325.0 | 244.2 | - | - | - | - | - | - | - | - | 123.3 | - | - |
|  |  |  |  | **2** | **nmol indivdual ^-1^d^-1^ x 10^2^** | - | - | 22.5 | - | 4.1 | - | 74.2 | 115.9 | - | 258.9 | 176.4 | - | - | - | - | - | - | - | - | - | - | - |
|  |  |  |  |  | **µmol g C^-1^ d^-1^** | - | - | 84.6 | - | 15.4 | - | 279.5 | 436.9 | - | 975.8 | 664.8 | - | - | - | - | - | - | - | - | - | - | - |
|  |  |  |  | **3** | **nmol indivdual ^-1^d^-1^ x 10^2^** | - | - | - | - | - | - | - | - | - | - | - | - | - | - | - | - | - | - | - | - | - | - |
|  |  |  |  |  | **µmol g C^-1^ d^-1^** | - | - | - | - | - | - | - | - | - | - | - | - | - | - | - | - | - | - | - | - | - | - |

**Table S3b.** Dissolved free amino acid release rates (nmol individual^-1^ d^-1^ x 10^2^ and µmol g C^-1^ d^-1^) by zooplankton communities during the summer (2015) in the coastal northern Adriatic Sea. The sum of DFAA (nmol individual^-1^ d^-1^ x 10^2^ and µmol g C^-1^ d^-1^), the ratios taurine/DFAA and the contribution of leucine to the total DFAA (%) are indicated. The green colored DFAA release rates did not increase significantly (*p* > 0.05, *R*^2^ < 0.6), as shown in Table S4. Replicates (R.) are indicated by numbers 1 to 3. The Tow depth (m), the tow time and temperature (T) are also indicated. Abbreviation for the amino acids are given in Table S3c.

| **Date** | **Depth** | **Time** | **T** | **R.** | **Unit** | **Asp** | **Glu** | **Asn** | **Ser** | **Gln** | **His** | **Gly** | **Arg** | **Thr** | **Ala** | **Tau** | **Gaba** | **Tyr** | **Met/Val** | **Try** | **Phe** | **Ile** | **Leu** | **Lys** | **DFAA** | **Tau/DFAA** | **Leu/DFAA %** |
| --- | --- | --- | --- | --- | --- | --- | --- | --- | --- | --- | --- | --- | --- | --- | --- | --- | --- | --- | --- | --- | --- | --- | --- | --- | --- | --- | --- |
| **11/03/15** | **25** | **11:00** | **20.0** | **1** | **nmol indivdual ^-1^d^-1^ x 10^2^** | 7.3 | 157.5 | 37.0 | 485.1 | 13.4 | 38.7 | 408.9 | 142.7 | 55.3 | 134.2 | 80.7 | - | 38.8 | 96.8 | 10.1 | 23.8 | 45.6 | 164.2 | 175.9 | 2035.3 | 0.04 | 8.07 |
|  |  |  |  |  | **µmol g C^-1^ d^-1^** | 102.5 | 2205.5 | 518.3 | 6793.8 | 188.0 | 541.9 | 5726.5 | 1998.8 | 773.8 | 1879.6 | 1130.2 | - | 543.7 | 1355.3 | 141.6 | 333.7 | 638.2 | 2299.1 | 2463.0 | 28503.1 | 0.04 | 8.07 |
|  |  |  |  | **2** | **nmol indivdual ^-1^d^-1^ x 10^2^** | - | 84.3 | 879.8 | 772.3 | 15.9 | 77.7 | 406.1 | 146.5 | 5.6 | - | 10.9 | - | - | - | - | - | - | 12.2 | - | 976.3 | 0.01 | 1.25 |
|  |  |  |  |  | **µmol g C^-1^ d^-1^** | - | 925.3 | 9652.4 | 8472.6 | 174.6 | 852.8 | 4455.6 | 1607.3 | 61.4 | - | 119.5 | - | - | - | - | - | - | 133.4 | - | 10711.1 | 0.01 | 1.25 |
|  |  |  |  | **3** | **nmol indivdual ^-1^d^-1^ x 10^2^** | - | - | - | - | - | - | 75.6 | - | 20.6 | - | 23.0 | - | - | - | - | - | 2.9 | 11.0 | - | 110.1 | 0.21 | 9.97 |
|  |  |  |  |  | **µmol g C^-1^ d^-1^** | - | - | - | - | - | - | 600.5 | - | 163.6 | - | 182.5 | - | - | - | - | - | 22.7 | 87.1 | - | 873.9 | 0.21 | 9.97 |
| **11/04/15** | **26** | **9:45** | **20.0** | **1** | **nmol indivdual ^-1^d^-1^ x 10^2^** | - | 332.6 | 131.5 | 1386.3 | 25.2 | 43.5 | 1471.1 | 49.5 | 315.1 | 127.9 | 472.0 | - | 50.6 | 49.0 | 29.7 | 53.0 | 32.4 | 28.9 | - | 2275.9 | 0.21 | 1.27 |
|  |  |  |  |  | **µmol g C^-1^ d^-1^** | - | 1202.1 | 475.4 | 5010.1 | 91.2 | 157.3 | 5316.5 | 178.9 | 1138.6 | 462.1 | 1705.7 | - | 182.7 | 177.2 | 107.4 | 191.4 | 117.2 | 104.5 | - | 8225.0 | 0.21 | 1.27 |
|  |  |  |  | **2** | **nmol indivdual ^-1^d^-1^ x 10^2^** | - | 142.8 | 602.6 | 86.9 | 307.1 | 78.4 | 422.1 | 40.5 | 138.0 | 130.0 | 411.5 | 1.8 | 30.7 | 61.3 | - | - | 27.5 | 36.1 | - | 1945.9 | 0.21 | 1.85 |
|  |  |  |  |  | **µmol g C^-1^ d^-1^** | - | 690.1 | 2912.0 | 420.2 | 1483.9 | 378.7 | 2040.0 | 195.7 | 667.0 | 628.2 | 1988.8 | 8.8 | 148.6 | 296.4 | - | - | 132.7 | 174.4 | - | 9404.2 | 0.21 | 1.85 |
|  |  |  |  | **3** | **nmol indivdual ^-1^d^-1^ x 10^2^** | - | 170.2 | 73.8 | 204.8 | 35.4 | 76.7 | 827.4 | 118.6 | 218.0 | 130.5 | 237.5 | 2.3 | 69.8 | 77.1 | 14.2 | 35.6 | 35.9 | 79.8 | - | 2169.8 | 0.11 | 3.68 |
|  |  |  |  |  | **µmol g C^-1^ d^-1^** | - | 911.8 | 395.7 | 1097.2 | 189.5 | 410.7 | 4433.0 | 635.2 | 1168.2 | 699.0 | 1272.7 | 12.3 | 373.7 | 412.8 | 75.8 | 190.8 | 192.4 | 427.4 | - | 11625.6 | 0.11 | 3.68 |
| **11/05/15** | **20** | **9:30** | **20.1** | **1** | **nmol indivdual ^-1^d^-1^ x 10^2^** | 27.8 | 117.5 | 116.9 | 265.9 | 30.0 | - | 658.7 | 137.8 | 167.2 | 157.7 | 186.9 | 2.1 | 54.6 | 80.1 | 18.9 | - | - | 117.1 | 201.1 | 2026.5 | 0.09 | 5.78 |
|  |  |  |  |  | **µmol g C^-1^ d^-1^** | 263.9 | 1114.2 | 1108.8 | 2522.6 | 284.7 | - | 6248.4 | 1307.3 | 1585.7 | 1495.7 | 1773.0 | 19.9 | 517.8 | 760.3 | 179.2 | - | - | 1111.0 | 1907.6 | 19223.6 | 0.09 | 5.78 |
|  |  |  |  | **2** | **nmol indivdual ^-1^d^-1^ x 10^2^** | 11.2 | 47.2 | 58.7 | 99.8 | 43.7 | 61.2 | 1385.9 | 648.2 | 440.4 | 236.6 | 591.7 | 4.0 | 61.0 | 58.4 | - | 35.5 | - | 59.8 | 115.7 | 2703.8 | - | 2.21 |
|  |  |  |  |  | **µmol g C^-1^ d^-1^** | 146.1 | 614.0 | 764.5 | 1298.6 | 569.1 | 796.3 | 18038.7 | 8437.0 | 5732.6 | 3079.1 | 7701.9 | 52.1 | 794.2 | 759.7 | - | 462.0 | - | 777.8 | 1506.3 | 35193.1 | - | 2.21 |
|  |  |  |  | **3** | **nmol indivdual ^-1^d^-1^ x 10^2^** | 30.9 | 1.4 | 160.6 | 285.8 | 34.3 | - | 7.7 | 111.9 | 2.9 | 1.2 | 2.5 | 2.9 | 0.2 | 0.3 | - | 0.1 | 0.7 | 98.9 | 0.7 | 15.3 | 0.17 | - |
|  |  |  |  |  | **µmol g C^-1^ d^-1^** | 328.4 | 61.4 | 1708.3 | 3040.6 | 364.8 | - | 341.5 | 1190.0 | 130.4 | 54.8 | 112.2 | 30.5 | 7.7 | 14.3 | - | 5.6 | 32.7 | 1052.0 | 30.3 | 678.6 | 0.17 | - |

**Table S3c.** Dissolved free amino acid release rates (nmol individual^-1^ d^-1^ x 10^2^ and µmol g C^-1^ d^-1^) by zooplankton communities during the fall (2015) in the coastal northern Adriatic Sea. The sum of DFAA (nmol individual^-1^ d^-1^ x 10^2^ and µmol g C^-1^ d^-1^), the ratios taurine/DFAA and the contribution of leucine to the total DFAA (%) are indicated. The green colored DFAA release rates did not increase significantly (*p* > 0.05, *R*^2^ < 0.6), as shown in Table S4. Replicates (R.) are indicated by numbers 1 to 3. The Tow depth (m), the tow time and temperature (T) are also indicated.

Abbreviations: St, station; R, replicates; Asp, Aspartic acid; Glu, Glutamic acid; Asn, Asparagine; Ser, Serine; Gln, Glutamine; His, Histine; Gly, Glycine; Arg, Arginine; Thr, Threonine; Ala, Alanine; Tau, Taurine; GABA, gamma aminobutyric acid; Tyr, Tyrosine; Met, Methionine; Val, Valine; Trp, Tryptophan; Phe, Phenylalanine; Ile, Isoleucine; Leu, Leucine; Lys, Lysine.

**Table S3d.** Dissolved free amino acid release rates (nmol individual^-1^ d^-1^ x 10^2^ and µmol g C^-1^ d^-1^) by zooplankton communities during the fall (2015) in the coastal northern Adriatic Sea. The sum of DFAA (nmol individual^-1^ d^-1^ x 10^2^ and µmol g C^-1^ d^-1^), the ratios taurine/DFAA and the contribution of leucine to the total DFAA (%) are indicated. The green colored DFAA release rates did not increase significantly (*p* > 0.05, *R*^2^ < 0.6), as shown in Table S4. Replicates (R.) are indicated by numbers 1 to 3. The Tow depth (m), the tow time and temperature (T) are also indicated. Abbreviation for the amino acids are given in Table S3c.

| **Date** | **Depth** | **Time** | **T** | **R.** | **Unit** | **Asp** | **Glu** | **Asn** | **Ser** | **Gln** | **His** | **Gly** | **Arg** | **Thr** | **Ala** | **Tau** | **Gaba** | **Tyr** | **Met/Val** | **Try** | **Phe** | **Ile** | **Leu** | **Lys** | **DFAA** | **Tau/DFAA** | **Leu/DFAA %** |
| --- | --- | --- | --- | --- | --- | --- | --- | --- | --- | --- | --- | --- | --- | --- | --- | --- | --- | --- | --- | --- | --- | --- | --- | --- | --- | --- | --- |
| **11/06/15** | **30** | **9:30** | **20.4** | **1** | **nmol indivdual ^-1^d^-1^ x 10^2^** | - | - | - | - | 3.7 | 20.6 | 140.9 | 35.4 | 31.0 | 0.1 | 136.7 | 3.5 | - | 17.3 | - | - | - | 21.2 | 60.3 | 582.5 | 0.23 | - |
|  |  |  |  |  | **µmol g C^-1^ d^-1^** | - | - | - | - | 26.2 | 144.9 | 992.8 | 249.3 | 218.1 | 1140.7 | 962.9 | 24.6 | - | 121.8 | - | - | - | 149.4 | 424.7 | 4103.6 | 0.23 | - |
|  |  |  |  | **2** | **nmol indivdual ^-1^d^-1^ x 10^2^** | - | 23.9 | - | - | 68.9 | 50.5 | 471.6 | - | 142.1 | - | 213.6 | - | 13.4 | 8.8 | - | - | 12.0 | 14.7 | 47.4 | 853.2 | 0.25 | 1.73 |
|  |  |  |  |  | **µmol g C^-1^ d^-1^** | - | 320.3 | - | - | 925.2 | 677.9 | 6332.5 | - | 1907.8 | - | 2868.2 | - | 180.4 | 118.1 | - | - | 160.8 | 197.8 | 636.2 | 11457.0 | 0.25 | 1.73 |
|  |  |  |  | **3** | **nmol indivdual ^-1^d^-1^ x 10^2^** | - | 76.7 | 58.9 | 384.4 | - | 68.9 | 1171.3 | - | 388.6 | 306.5 | 261.7 | - | 50.5 | - | 70.2 | 17.9 | 43.1 | 53.0 | 20.6 | 1945.3 | 0.13 | 2.72 |
|  |  |  |  |  | **µmol g C^-1^ d^-1^** | - | 821.8 | 630.9 | 4119.8 | - | 738.1 | 12552.4 | - | 4164.5 | 3285.1 | 2804.9 | - | 541.7 | - | 752.0 | 191.4 | 461.7 | 567.8 | 220.7 | 20846.3 | 0.13 | 2.72 |
| **11/07/15** | **25** | **9:30** | **20.2** | **1** | **nmol indivdual ^-1^d^-1^ x 10^2^** | - | 28.7 | 14.3 | 29.5 | 5.1 | 40.1 | 248.5 | 28.3 | 87.5 | 118.8 | 157.8 | 1.9 | 19.0 | 20.9 | 3.9 | 10.6 | 13.0 | 22.6 | 59.5 | 752.0 | 0.21 | 3.01 |
|  |  |  |  |  | **µmol g C^-1^ d^-1^** | - | 243.3 | 121.0 | 250.5 | 43.2 | 339.9 | 2108.4 | 240.3 | 742.2 | 1008.1 | 1338.3 | 15.7 | 160.9 | 177.3 | 32.8 | 89.6 | 109.9 | 191.7 | 504.4 | 6379.2 | 0.21 | 3.01 |
|  |  |  |  | **2** | **nmol indivdual ^-1^d^-1^ x 10^2^** | - | 36.3 | 20.2 | 85.0 | 12.7 | 38.3 | 451.4 | 75.5 | 94.0 | 90.4 | 143.4 | - | 18.5 | 23.6 | 2.5 | 15.9 | 14.4 | 20.8 | 62.3 | 986.2 | 0.15 | 2.10 |
|  |  |  |  |  | **µmol g C^-1^ d^-1^** | - | 213.3 | 118.7 | 499.6 | 74.9 | 225.4 | 2653.6 | 444.1 | 552.8 | 531.2 | 843.2 | - | 108.6 | 139.0 | 14.5 | 93.4 | 84.4 | 122.0 | 366.3 | 5797.6 | 0.15 | 2.10 |
|  |  |  |  | **3** | **nmol indivdual ^-1^d^-1^ x 10^2^** | - | 67.8 | 30.3 | 82.9 | 37.8 | 133.7 | 1039.6 | 35.5 | 368.4 | 260.5 | 249.7 | 3.4 | 61.2 | 37.3 | 6.2 | 48.5 | 26.9 | 64.4 | 191.6 | 2496.2 | 0.10 | 2.58 |
|  |  |  |  |  | **µmol g C^-1^ d^-1^** | - | 421.6 | 188.7 | 515.7 | 235.1 | 831.4 | 6463.9 | 220.7 | 2290.3 | 1619.5 | 1552.5 | 21.3 | 380.8 | 231.7 | 38.7 | 301.5 | 167.1 | 400.5 | 1191.3 | 15519.9 | 0.10 | 2.58 |
| **11/10/15** | **26** | **9:30** | **20.2** | **1** | **nmol indivdual ^-1^d^-1^ x 10^2^** | 28.0 | 41.0 | 20.6 | 46.7 | 31.6 | 34.8 | 581.9 | 28.8 | 166.4 | 59.7 | 206.3 | - | 25.5 | 28.5 | 5.8 | 10.1 | 24.9 | 28.2 | 86.6 | 1249.0 | 0.17 | 2.26 |
|  |  |  |  |  | **µmol g C^-1^ d^-1^** | 82.0 | 119.9 | 60.2 | 136.7 | 92.4 | 101.8 | 1703.3 | 84.4 | 487.0 | 174.6 | 603.8 | - | 74.5 | 83.5 | 16.8 | 29.7 | 73.0 | 82.6 | 253.3 | 3655.8 | 0.17 | 2.26 |
|  |  |  |  | **2** | **nmol indivdual ^-1^d^-1^ x 10^2^** | 35.1 | 70.0 | 26.4 | 32.9 | 20.7 | 84.0 | 126.0 | 34.1 | 65.1 | 65.1 | 203.0 | 2.5 | 19.5 | 5.7 | 13.8 | 17.5 | 17.1 | 23.8 | 88.1 | 747.4 | 0.27 | 3.18 |
|  |  |  |  |  | **µmol g C^-1^ d^-1^** | 4.4 | 8.7 | 3.3 | 4.1 | 2.6 | 10.5 | 15.7 | 4.2 | 8.1 | 8.1 | 25.3 | 0.3 | 2.4 | 0.7 | 1.7 | 2.2 | 2.1 | 3.0 | 11.0 | 93.1 | 0.27 | 3.18 |
|  |  |  |  | **3** | **nmol indivdual ^-1^d^-1^ x 10^2^** | 70.1 | 86.1 | 57.9 | 60.6 | 54.9 | 169.7 | 1804.9 | 51.6 | 563.9 | 171.6 | 514.3 | 41.3 | 60.2 | 53.5 | 8.5 | 32.1 | 72.6 | 50.0 | 204.6 | 3614.0 | 0.14 | 1.38 |
|  |  |  |  |  | **µmol g C^-1^ d^-1^** | 213.5 | 261.9 | 176.2 | 184.4 | 167.1 | 516.7 | 5494.2 | 157.0 | 1716.5 | 522.4 | 1565.6 | 125.8 | 183.2 | 162.8 | 25.8 | 97.8 | 220.9 | 152.1 | 622.9 | 11001.1 | 0.14 | 1.38 |

| **Date** | **Depth** | **Time** | **T** | **R.** | **Unit** | **Asp** | **Glu** | **Asn** | **Ser** | **Gln** | **His** | **Gly** | **Arg** | **Thr** | **Ala** | **Tau** | **Gaba** | **Tyr** | **Met/Val** | **Try** | **Phe** | **Ile** | **Leu** | **Lys** | **DFAA** | **Tau/DFAA** | **Leu/DFAA %** |
| --- | --- | --- | --- | --- | --- | --- | --- | --- | --- | --- | --- | --- | --- | --- | --- | --- | --- | --- | --- | --- | --- | --- | --- | --- | --- | --- | --- |
| **02/02/16** | **20** | **9:30** | **12.0** | **1** | **nmol indivdual ^-1^d^-1^ x 10^2^** | 16.1 | 48.9 | 14.0 | - | 3.5 | - | 650.8 | 15.2 | 53.9 | 49.5 | 133.5 | 1.4 | 5.3 | 2.2 | - | - | 12.2 | 13.8 | 42.7 | 929.5 | 0.14 | 1.49 |
|  |  |  |  |  | **µmol g C^-1^ d^-1^** | 140.8 | 428.1 | 122.2 | - | 30.8 | - | 5701.0 | 133.5 | 472.3 | 433.9 | 1169.7 | 12.0 | 46.7 | 19.0 | - | - | 107.0 | 121.1 | 374.2 | 8142.7 | 0.14 | 1.49 |
|  |  |  |  | **2** | **nmol indivdual ^-1^d^-1^ x 10^2^** | 29.0 | 40.2 | 38.5 | - | - | 104.8 | 704.1 | 33.3 | 131.5 | 57.3 | 171.8 | 1.1 | - | - | - | - | - | 16.9 | 105.1 | 1157.0 | 0.15 | 1.46 |
|  |  |  |  |  | **µmol g C^-1^ d^-1^** | 378.9 | 525.9 | 503.1 | - | - | 1371.1 | 9207.3 | 435.7 | 1720.0 | 749.0 | 2246.8 | 14.7 | - | - | - | - | - | 220.7 | 1374.8 | 15130.1 | 0.15 | 1.46 |
|  |  |  |  | **3** | **nmol indivdual ^-1^d^-1^ x 10^2^** | - | - | 30.5 | - | - | - | 286.4 | - | - | 15.5 | 191.2 | - | - | - | - | - | - | 17.6 | - | 319.5 | 0.60 | 5.51 |
|  |  |  |  |  | **µmol g C^-1^ d^-1^** | - | - | 263.9 | - | - | - | 2476.0 | - | - | 133.8 | 1653.1 | - | - | - | - | - | - | 152.3 | - | 2762.1 | 0.60 | 5.51 |
| **02/03/16** | **24** | **9:30** | **12.1** | **1** | **nmol indivdual ^-1^d^-1^ x 10^2^** | - | - | 10.3 | - | 25.1 | - | 433.3 | 5.6 | 79.1 | 19.7 | 4.8 | - | - | - | - | - | - | 17.5 | 23.3 | 614.0 | - | 2.85 |
|  |  |  |  |  | **µmol g C^-1^ d^-1^** | - | - | 82.5 | - | 200.6 | - | 3461.1 | 44.9 | 631.9 | 157.1 | 38.3 | - | - | - | - | - | - | 139.8 | 186.4 | 4904.3 | - | 2.85 |
|  |  |  |  | **2** | **nmol indivdual ^-1^d^-1^ x 10^2^** | - | - | - | - | 2.1 | 82.4 | 186.1 | 10.1 | 32.6 | 165.7 | 41.4 | - | - | - | - | - | - | - | - | 384.3 | 0.11 | - |
|  |  |  |  |  | **µmol g C^-1^ d^-1^** | - | - | - | - | 21.2 | 840.1 | 1898.3 | 103.2 | 332.2 | 1690.2 | 422.2 | - | - | - | - | - | - | - | - | 3920.7 | 0.11 | - |
|  |  |  |  | **3** | **nmol indivdual ^-1^d^-1^ x 10^2^** | - | - | 10.5 | - | 1.5 | - | 353.5 | 64.5 | - | 16.0 | 121.2 | - | - | - | - | - | - | - | - | 446.1 | 0.27 | - |
|  |  |  |  |  | **µmol g C^-1^ d^-1^** | - | - | 42.3 | - | 6.2 | - | 1421.3 | 259.5 | - | 64.5 | 487.2 | - | - | - | - | - | - | - | - | 1793.7 | 0.27 | - |
| **02/05/16** | **25** | **9:30** | **12.0** | **1** | **nmol indivdual ^-1^d^-1^ x 10^2^** | 414.7 | 308.5 | 215.0 | 228.0 | 264.2 | 79.6 | 5395.1 | 187.4 | 1461.1 | 728.2 | 481.2 | - | 206.8 | 351.7 | - | 311.9 | 294.4 | 93.3 | 461.4 | 10355.2 | 0.05 | 0.90 |
|  |  |  |  |  | **µmol g C^-1^ d^-1^** | 1483.2 | 1103.4 | 768.9 | 815.4 | 944.7 | 284.7 | 19295.2 | 670.4 | 5225.5 | 2604.2 | 1721.0 | - | 739.4 | 1257.8 | - | 1115.4 | 1053.0 | 333.6 | 1650.2 | 37034.3 | 0.05 | 0.90 |
|  |  |  |  | **2** | **nmol indivdual ^-1^d^-1^ x 10^2^** | 45.1 | - | 45.1 | 41.7 | 59.3 | - | 848.0 | 27.0 | 152.6 | 133.2 | 208.6 | 15.3 | 25.4 | 758.8 | - | - | - | 24.5 | 97.0 | 1514.1 | 0.14 | 1.61 |
|  |  |  |  |  | **µmol g C^-1^ d^-1^** | 212.7 | - | 212.7 | 196.8 | 279.6 | - | 3999.2 | 127.3 | 719.4 | 628.0 | 983.9 | 72.3 | 119.7 | 3578.4 | - | - | - | 115.3 | 457.2 | 7140.2 | 0.14 | 1.61 |
|  |  |  |  | **3** | **nmol indivdual ^-1^d^-1^ x 10^2^** | - | - | - | - | - | - | 68.4 | - | - | - | 10.8 | - | - | - | - | - | - | - | - | 68.4 | 0.16 | - |
|  |  |  |  |  | **µmol g C^-1^ d^-1^** | - | - | - | - | - | - | 129.5 | - | - | - | 20.4 | - | - | - | - | - | - | - | - | 129.5 | 0.16 | - |
| **02/09/16** | **28** | **9:30** | **12.1** | **1** | **nmol indivdual ^-1^d^-1^ x 10^2^** | 46.6 | 43.1 | 41.8 | 162.7 | 25.1 | 40.1 | 751.3 | 63.1 | 121.6 | 160.5 | 179.4 | - | - | - | 70.6 | 11.2 | - | 38.5 | 77.5 | 1653.8 | 0.11 | 2.33 |
|  |  |  |  |  | **µmol g C^-1^ d^-1^** | 199.2 | 184.2 | 178.9 | 695.3 | 107.2 | 171.4 | 3211.2 | 269.9 | 519.9 | 686.0 | 766.6 | - | - | - | 301.6 | 48.1 | - | 164.5 | 331.1 | 7068.4 | 0.11 | 2.33 |
|  |  |  |  | **2** | **nmol indivdual ^-1^d^-1^ x 10^2^** | 16.0 | 13.8 | 40.1 | 30.0 | 13.7 | 23.1 | 540.8 | 181.2 | 127.9 | 43.5 | 138.4 | - | 8.3 | - | 34.5 | 7.2 | - | - | 45.8 | 1102.8 | 0.13 | - |
|  |  |  |  |  | **µmol g C^-1^ d^-1^** | 58.9 | 51.0 | 147.9 | 110.9 | 50.6 | 85.3 | 1997.4 | 669.2 | 472.3 | 160.6 | 511.2 | - | 30.6 | - | 127.5 | 26.6 | - | - | 169.1 | 4072.7 | 0.13 | - |
|  |  |  |  | **3** | **nmol indivdual ^-1^d^-1^ x 10^2^** | - | 5.0 | 22.8 | - | 87.6 | 23.0 | 350.0 | 20.9 | 166.5 | 39.6 | 151.3 | - | 12.6 | - | - | - | - | - | 26.2 | 754.1 | 0.20 | - |
|  |  |  |  |  | **µmol g C^-1^ d^-1^** | - | 16.6 | 75.8 | - | 291.3 | 76.5 | 1163.7 | 69.4 | 553.5 | 131.5 | 503.0 | - | 41.9 | - | - | - | - | - | 87.0 | 2507.2 | 0.20 | - |

**Table S3e.** Dissolved free amino acid release rates (nmol individual^-1^ d^-1^ x 10^2^ and µmol g C^-1^ d^-1^) by zooplankton communities during the winter (2016) in the coastal northern Adriatic Sea. The sum of DFAA (nmol individual^-1^ d^-1^ x 10^2^ and µmol g C^-1^ d^-1^), the ratios taurine/DFAA and the contribution of leucine to the total DFAA (%) are indicated. The green colored DFAA release rates did not increase significantly (*p* > 0.05, *R*^2^ < 0.6), as shown in Table S4. Replicates (R.) are indicated by numbers 1 to 3. The Tow depth (m), the tow time and temperature (T) are also indicated. Abbreviation for the amino acids are given in Table S3c.

| **Date** | **R.** |  | **Asp** | **Glu** | **Asn** | **Ser** | **Gln** | **His** | **Gly** | **Arg** | **Thr** | **Ala** | **Tau** | **Gaba** | **Tyr** | **Met/Val** | **Try** | **Phe** | **Ile** | **Leu** | **Lys** |
| --- | --- | --- | --- | --- | --- | --- | --- | --- | --- | --- | --- | --- | --- | --- | --- | --- | --- | --- | --- | --- | --- |
| **04/21/15** | **1** | ***R^2^*** | 0.966 | 0.860 | - | 0.547 | - | - | 0.582 | 0.589 | - | 0.583 | 0.761 | - | - | - | - | - | - | - | - |
|  |  | **h** | 1.5 | 1.0 | - | 1.5 | - | - | 1.5 | 1.5 | - | 1.5 | 1.5 | - | - | - | - | - | - | - | - |
|  |  | ***p*** | 0.02 | 0.24 | - | 0.30 | - | - | 0.24 | 0.23 | - | 0.24 | 0.13 | - | - | - | - | - | - | - | - |
|  | **2** | ***R^2^*** | - | - | - | - | - | - | 0.914 | 0.788 | 0.808 | 0.699 | 0.868 | - | - | - | - | - | - | - | - |
|  |  | **h** | - | - | - | - | - | - | 5.0 | 1.5 | 1.5 | 1.5 | 8.0 | - | - | - | - | - | - | - | - |
|  |  | ***p*** | - | - | - | - | - | - | 0.01 | 0.11 | 0.10 | 0.02 | 0.00 | - | - | - | - | - | - | - | - |
| **04/22/15** | **1** | ***R^2^*** | - | - | - | - | - | - | 0.632 | - | 0.830 | - | 0.811 | - | - | - | - | - | - | - | - |
|  |  | **h** | - | - | - | - | - | - | 8.0 | - | 8.0 | - | 8.0 | - | - | - | - | - | - | - | - |
|  |  | ***p*** | - | - | - | - | - | - | 0.02 | - | 0.00 | - | 0.01 | - | - | - | - | - | - | - | - |
|  | **2** | ***R^2^*** | - | - | - | - | 0.726 | - | 0.884 | - | 0.875 | - | 0.849 | - | - | - | - | - | - | - | - |
|  |  | **h** | - | - | - | - | 8.0 | - | 8.0 | - | 8.0 | - | 8.0 | - | - | - | - | - | - | - | - |
|  |  | ***p*** | - | - | - | - | 0.01 | - | 0.02 | - | 0.00 | - | 0.00 | - | - | - | - | - | - | - | - |
|  | **3** | ***R^2^*** | - | - | - | - | - | - | 0.975 | - | - | - | 0.732 | - | - | - | - | - | - | - | - |
|  |  | **h** | - | - | - | - | - | - | 8.0 | - | - | - | 8.0 | - | - | - | - | - | - | - | - |
|  |  | ***p*** | - | - | - | - | - | - | 0.00 | - | - | - | 0.03 | - | - | - | - | - | - | - | - |
| **04/27/15** | **1** | ***R^2^*** | - | 0.780 | - | 0.753 | - | - | 0.718 | 0.823 | 0.841 | 0.803 | 0.856 | - | - | - | - | - | - | - | 0.782 |
|  |  | **h** | - | 5.0 | - | 1.5 | - | - | 5.0 | 1.5 | 5.0 | 5.0 | 5.0 | - | - | - | - | - | - | - | 2.0 |
|  |  | ***p*** | - | 0.01 | - | 0.13 | - | - | 0.02 | 0.09 | 0.00 | 0.01 | 0.00 | - | - | - | - | - | - | - | 0.08 |
|  | **2** | ***R^2^*** | - |  | - |  | - | - | 0.938 | - | 0.312 | 0.690 | 0.895 | - | - | - | - | - | - | - | - |
|  |  | **h** | - |  | - |  | - | - | 3.0 | - | 3.0 | 3.0 | 3.0 | - | - | - | - | - | - | - | - |
|  |  | ***p*** | - |  | - |  | - | - | 0.01 | - | 0.00 | 0.04 | 0.00 | - | - | - | - | - | - | - | - |
|  | **3** | ***R^2^*** | - |  | - | 0.794 | - | - | 0.862 | 0.829 | 0.741 | 0.857 | 0.748 | - | - | - | - | - | - | 0.83 | - |
|  |  | **h** | - |  | - | 3.0 | - | - | 3.0 | 3.0 | 3.0 | 3.0 | 2.0 | - | - | - | - | - | - | 3.0 | - |
|  |  | ***p*** | - |  | - | 0.02 | - | - | 0.01 | 0.01 | 0.03 | 0.01 | 0.06 | - | - | - | - | - | - | 0.01 | - |
| **04/28/15** | **1** | ***R^2^*** | - |  | - | - | - | - | 0.914 | 0.788 | 0.808 | 0.699 | 0.868 | - | - | - | - | - | - | - | - |
|  |  | **h** | - | - | - | - | - | - | 5.0 | 1.5 | 1.5 | 1.5 | 8.0 | - | - | - | - | - | - | - | - |
|  |  | ***p*** | - | - | - | - | - | - | 0.01 | 0.11 | 0.10 | 0.02 | 0.00 | - | - | - | - | - | - | - | - |
|  | **2** | ***R^2^*** | - | - | - | - | - | - | 0.888 | - | 0.842 | - | 0.912 | - | - | - | - | - | - | - | - |
|  |  | **h** | - | - | - | - | - | - | 8.0 | - | 8.0 | - | 8.0 | - | - | - | - | - | - | - | - |
|  |  | ***p*** | - | - | - | - | - | - | 0.00 | - | 0.01 | - | 0.04 | - | - | - | - | - | - | - | - |
|  | **3** | ***R^2^*** | - | - | - | - | - | - | 0.843 | - | 0.825 | - | 0.822 | - | - | - | - | - | - | - | - |
|  |  | **h** | - | - | - | - | - | - | 5.0 | - | 5.0 | - | 5.0 | - | - | - | - | - | - | - | - |
|  |  | ***p*** | - | - | - | - | - | - | 0.01 | - | 0.03 | - | 0.03 | - | - | - | - | - | - | - | - |

**Table S4a.** *p*-values and coefficients of determination (*R^2^*) of the release of taurine and primary amino acids by crustacean zooplankton in the coastal northern Adriatic Sea (spring). Release rates were calculated by linear regression analyses until the time point when the amino acid species or taurine did not further increase in concentration. R. indicates the number of replicates. Abbreviation for the amino acids are given in Table S3c.

| **Date** | **R.** |  | **Asp** | **Glu** | **Asn** | **Ser** | **Gln** | **His** | **Gly** | **Arg** | **Thr** | **Ala** | **Tau** | **Gaba** | **Tyr** | **Met/Val** | **Try** | **Phe** | **Ile** | **Leu** | **Lys** |
| --- | --- | --- | --- | --- | --- | --- | --- | --- | --- | --- | --- | --- | --- | --- | --- | --- | --- | --- | --- | --- | --- |
| **06/24/15** | **1** | ***R^2^*** | - | - | - | 0.961 | - | - | 0.712 | - | - | 0.874 | 0.957 | - | - | - | - | - | - | - | - |
|  |  | **h** | - | - | - | 1.0 | - | - | 8.0 | - | - | 1.0 | 8.0 | - | - | - | - | - | - | - | - |
|  |  | ***p*** | - | - | - | 0.13 | - | - | 0.03 | - | - | 0.23 | 0.00 | - | - | - | - | - | - | - | - |
|  | **2** | ***R^2^*** | 0.865 | 0.885 | - | 0.772 | - | - | 0.987 | 0.895 | 0.934 | 0.730 | 0.919 | - | - | 0.876 | - | 0.843 | - | 0.880 | - |
|  |  | **h** | 2.0 | 2.0 | - | 3.0 | - | - | 3.0 | 3.0 | 3.0 | 2.0 | 3.0 | - | - | 2.0 | - | 2.0 | - | 2.0 | - |
|  |  | ***p*** | 0.07 | 0.06 | - | 0.12 | - | - | 0.01 | 0.00 | 0.00 | 0.07 | 0.01 | - | - | 0.06 | - | 0.08 | - | 0.06 | - |
|  | **3** | ***R^2^*** | - | 0.844 | - | 0.711 | 0.645 | 0.645 | 0.909 | 0.844 | 0.959 | 0.908 | 0.954 | - | 0.848 | - | - | - | - | 0.89 | - |
|  |  | **h** | - | 3.0 | - | 8.0 | 2.0 | 2.0 | 3.0 | 3.0 | 3.0 | 3.0 | 3.0 | - | 3.0 | - | - | - | - | 3.0 | - |
|  |  | ***p*** | - | 0.03 | - | 0.01 | 0.10 | 0.10 | 0.00 | 0.01 | 0.01 | 0.00 | 0.01 | - | 0.06 | - | - | - | - | 0.02 | - |
| **06/25/15** | **1** | ***R^2^*** | - | - | - | - | - | 0.916 | 0.985 | - | 0.966 | - | 0.929 | - | 0.951 | 0.805 | 0.942 | - | - | 0.958 | 0.963 |
|  |  | **h** | - | - | - | - | - | 3.0 | 3.0 | - | 3.0 | - | 3.0 | - | 3.0 | 1.0 | 1.0 | - | - | 3.0 | 1.0 |
|  |  | ***p*** | - | - | - | - | - | 0.01 | 0.01 | - | 0.02 | - | 0.01 | - | 0.00 | 0.29 | 0.15 | - | - | 0.02 | 0.12 |
|  | **2** | ***R^2^*** | - | - | - | - | - | - | 0.824 | - | 0.872 | 0.760 | 0.860 | - | - | - | - | - | - | - | - |
|  |  | **h** | - | - | - | - | - | - | 2.0 | - | 3.0 | 3.0 | 2.0 | - | - | - | - | - | - | - | - |
|  |  | ***p*** | - | - | - | - | - | - | 0.03 | - | 0.01 | 0.05 | 0.02 | - | - | - | - | - | - | - | - |
|  | **3** | ***R^2^*** | 0.824 | 0.823 | 0.978 | 0.745 | 0.967 | 0.961 | 0.735 | 0.745 | 1.000 | 0.804 | 0.998 | - | - | - | - | 0.867 | 0.987 | 0.912 | - |
|  |  | **h** | 1.0 | 1.0 | 1.0 | 1.0 | 1.0 | 1.0 | 3.0 | 1.0 | 1.0 | 1.0 | 1.0 | - | - | - | - | 1.0 | 1.0 | 1.0 | - |
|  |  | ***p*** | 0.28 | 0.28 | 0.10 | 0.34 | 0.12 | 0.13 | 0.03 | 0.34 | 0.01 | 0.29 | 0.03 | - | - | - | - | 0.24 | 0.07 | 0.19 | - |
| **06/27/15** | **1** | ***R^2^*** | - | - | - | - | - | - | 0.935 | - | - | 0.837 | 0.879 | - | - | - | - | - | - | - | - |
|  |  | **h** | - | - | - | - | - | - | 3.0 | - | - | 2.0 | 2.0 | - | - | - | - | - | - | - | - |
|  |  | ***p*** | - | - | - | - | - | - | 0.00 | - | - | 0.08 | 0.06 | - | - | - | - | - | - | - | - |
|  | **2** | ***R^2^*** | 0.959 | 0.667 | - | 0.741 | 0.742 | 0.922 | 0.888 | 0.913 | 0.945 | 0.901 | 0.891 | - | 0.885 | 0.867 | 0.921 | 0.903 | 0.984 | 0.851 | 0.895 |
|  |  | **h** | 5.0 | 3.0 | - | 3.0 | 2.0 | 2.0 | 3.0 | 3.0 | 3.0 | 3.0 | 3.0 | - | 3.0 | 3.0 | 2.0 | 3.0 | 3.0 | 3.0 | 3.0 |
|  |  | ***p*** | 0.00 | 0.09 | - | 0.06 | 0.06 | 0.01 | 0.00 | 0.00 | 0.00 | 0.00 | 0.00 | - | 0.01 | 0.01 | 0.01 | 0.00 | 0.00 | 0.01 | 0.00 |
|  | **3** | ***R^2^*** | 0.920 | 0.828 | - | 0.973 | - | 0.778 | 0.929 | 0.894 | 0.821 | 0.826 | 0.936 | - | 0.737 | 0.911 | 0.897 | 0.974 | 0.972 | 0.974 | 0.956 |
|  |  | **h** | 1.0 | 1.0 | - | 1.0 | - | 1.0 | 1.0 | 1.0 | 1.0 | 1.0 | 1.0 | - | 1.0 | 1.0 | 1.0 | 1.0 | 1.0 | 1.0 | 1.0 |
|  |  | ***p*** | 0.18 | 0.27 | - | 0.11 | - | 0.31 | 0.17 | 0.21 | 0.28 | 0.27 | 0.16 | - | 0.03 | 0.19 | 0.21 | 0.10 | 0.11 | 0.10 | 0.13 |
| **06/28/15** | **1** | ***R^2^*** | 0.699 | 0.624 | 0.676 | 0.648 | 0.901 | 0.850 | 0.656 | 0.671 | 0.935 | 0.841 | 0.730 | - | - | - | - | - | - | - | - |
|  |  | **h** | 1.5 | 1.5 | 1.5 | 1.5 | 1.5 | 1.5 | 1.5 | 1.5 | 1.0 | 1.0 | 1.5 | - | - | - | - | - | - | - | - |
|  |  | ***p*** | 0.16 | 0.21 | 0.18 | 0.20 | 0.05 | 0.08 | 0.19 | 0.16 | 0.16 | 0.26 | 0.15 | - | - | - | - | - | - | - | - |
|  | **2** | ***R^2^*** | - | - | 0.953 | - | 0.908 | - | 0.820 | 0.845 | - | 0.808 | 0.846 | - | - | - | - | - | - | - | - |
|  |  | **h** | - | - | 1.0 | - | 1.0 | - | 1.0 | 1.0 | - | 1.0 | 1.0 | - | - | - | - | - | - | - | - |
|  |  | ***p*** | - | - | 0.14 | - | 0.20 | - | 0.42 | 0.26 | - | 0.10 | 0.26 | - | - | - | - | - | - | - | - |
|  | **3** | ***R^2^*** | - | - | - | - | - | - | - | - | - | - | - | - | - | - | - | - | - | - | - |
|  |  | **h** | - | - | - | - | - | - | - | - | - | - | - | - | - | - | - | - | - | - | - |
|  |  | ***p*** | - | - | - | - | - | - | - | - | - | - | - | - | - | - | - | - | - | - | - |

**Table S4b.** *p*-values and coefficients of determinations (*R^2^*) of the release of taurine and primary amino acids by crustacean zooplankton in the coastal northern Adriatic Sea (summer). Release rates were calculated by linear regression analyses until the time point when the amino acid species or taurine did not further increase in concentration. R. indicates the number of replicates. Abbreviation for the amino acids are given in Table S3c.

| **Date** | **R.** |  | **Asp** | **Glu** | **Asn** | **Ser** | **Gln** | **His** | **Gly** | **Arg** | **Thr** | **Ala** | **Tau** | **Gaba** | **Tyr** | **Met/Val** | **Try** | **Phe** | **Ile** | **Leu** | **Lys** |
| --- | --- | --- | --- | --- | --- | --- | --- | --- | --- | --- | --- | --- | --- | --- | --- | --- | --- | --- | --- | --- | --- |
| **11/03/15** | **1** | ***R^2^*** | 0.851 | 0.995 | 0.988 | 0.974 | 0.902 | 0.973 | 0.960 | 0.981 | 0.903 | 0.947 | 0.918 | - | 0.971 | 0.984 | 0.956 | 0.697 | 0.804 | 0.973 | 0.910 |
|  |  | **h** | 8.0 | 5.0 | 5.0 | 5.0 | 5.0 | 5.0 | 5.0 | 5.0 | 5.0 | 5.0 | 5.0 | - | 5.0 | 5.0 | 8.0 | 5.0 | 3.0 | 5.0 | 5.0 |
|  |  | ***p*** | 0.01 | 0.00 | 0.00 | 0.00 | 0.01 | 0.00 | 0.00 | 0.00 | 0.01 | 0.00 | 0.01 | - | 0.00 | 0.00 | 0.00 | 0.04 | 0.04 | 0.00 | 0.01 |
|  | **2** | ***R^2^*** | - | 0.904 | 0.815 | 0.830 | 0.883 | 0.834 | 0.836 | 0.844 | 0.908 | - | 0.814 | - | - | - | - | - | - | 0.802 | - |
|  |  | **h** | - | 2.0 | - | 1.0 | 1.0 | 1.0 | 1.0 | 1.0 | 1.0 | - | 8.0 | - | - | - | - | - | - | 8.0 | - |
|  |  | ***p*** | - | 0.04 | - | 0.27 | 0.22 | 0.27 | 0.27 | 0.26 | 0.06 | - | 0.01 | - | - | - | - | - | - | 0.02 | - |
|  | **3** | ***R^2^*** | - | - | - | - | - | - | 0.756 | - | 0.821 | - | 0.665 | - | - | - | - | - | 0.888 | 0.859 | - |
|  |  | **h** | - | - | - | - | - | - | 8.0 | - | 8.0 | - | 8.0 | - | - | - | - | - | 8.0 | 8.0 | - |
|  |  | ***p*** | - | - | - | - | - | - | 0.01 | - | 0.00 | - | 0.03 | - | - | - | - | - | 0.00 | 0.00 | - |
| **11/04/15** | **1** | ***R^2^*** | - | 0.981 | 0.872 | 0.814 | 0.945 | 0.839 | 0.863 | 0.937 | 0.952 | 0.942 | 0.956 | - | 0.851 | 0.918 | 0.759 | 0.779 | 0.904 | 0.844 | - |
|  |  | **h** | - | 1.0 | 1.5 | 1.0 | 8.0 | 5.0 | 3.0 | 8.0 | 3.0 | 3.0 | 3.0 | - | 3.0 | 8.0 | 8.0 | 5.0 | 8.0 | 8.0 | - |
|  |  | ***p*** | - | 0.09 | 0.05 | 0.28 | 0.00 | 0.00 | 0.01 | 0.00 | 0.00 | 0.01 | 0.00 | - | 0.03 | 0.00 | 0.00 | 0.02 | 0.00 | 0.00 | - |
|  | **2** | ***R^2^*** | - | 0.689 | 0.796 | 0.821 | 0.872 | 0.708 | 0.932 | 0.928 | 0.921 | 0.934 | 0.886 | 0.956 | 0.948 | 0.916 | - | - | 0.758 | 0.903 | - |
|  |  | **h** | - | 3.0 | 5.0 | 8.0 | 5.0 | 3.0 | 8.0 | 8.0 | 5.0 | 5.0 | 5.0 | 5.0 | 5.0 | 8.0 | - | - | 5.0 | 5.0 | - |
|  |  | ***p*** | - | 0.08 | 0.04 | 0.01 | 0.02 | 0.16 | 0.00 | 0.00 | 0.01 | 0.01 | 0.02 | 0.00 | 0.01 | 0.00 | - | - | 0.05 | 0.01 | - |
|  | **3** | ***R^2^*** | - | 0.903 | 0.950 | 0.808 | 0.914 | 0.693 | 0.929 | 0.840 | 0.953 | 0.877 | 0.881 | 0.807 | 0.987 | 0.878 | 0.713 | 0.979 | 0.918 | 0.915 | - |
|  |  | **h** | - | 3.0 | 3.0 | 3.0 | 3.0 | 3.0 | 3.0 | 3.0 | 3.0 | 3.0 | 3.0 | 5.0 | 3.0 | 3.0 | 3.0 | 3.0 | 2.0 | 3.0 | - |
|  |  | ***p*** | - | 0.01 | 0.01 | 0.01 | 0.01 | 0.01 | 0.00 | 0.01 | 0.00 | 0.01 | 0.01 | 0.01 | 0.00 | 0.01 | 0.03 | 0.00 | 0.01 | 0.00 | - |
| **11/05/15** | **1** | ***R^2^*** | 0.876 | 0.971 | 0.889 | 0.966 | 0.926 | - | 0.970 | 0.886 | 0.832 | 0.913 | 0.936 | 0.813 | 0.893 | 0.692 | 0.722 | - | - | 0.906 | 0.857 |
|  |  | **h** | 2.0 | 2.0 | 3.0 | 3.0 | 3.0 | - | 3.0 | 2.0 | 2.0 | 3.0 | 3.0 | 3.0 | 3.0 | 2.0 | 2.0 | - | - | 2.0 | 2.0 |
|  |  | ***p*** | 0.06 | 0.00 | 0.00 | 0.00 | 0.00 | - | 0.00 | 0.02 | 0.01 | 0.01 | 0.00 | 0.01 | 0.01 | 0.08 | 0.15 | - | - | 0.01 | 0.02 |
|  | **2** | ***R^2^*** | 0.869 | 0.798 | 0.953 | 0.765 | 0.954 | 0.749 | 0.886 | 0.656 | 0.976 | 0.917 | 0.909 | 0.754 | 0.990 | 0.943 | - | 0.974 | - | 0.973 | 0.935 |
|  |  | **h** | 2.0 | 5.0 | 3.0 | 5.0 | 2.0 | 3.0 | 2.0 | 1.5 | 2.0 | 1.5 | 1.5 | 1.5 | 2.0 | 2.0 | - | 2.0 | - | 2.0 | 3.0 |
|  |  | ***p*** | 0.07 | 0.01 | 0.00 | 0.01 | 0.00 | 0.03 | 0.02 | 0.19 | 0.00 | 0.04 | 0.05 | 0.13 | 0.00 | 0.01 | - | 0.00 | - | 0.00 | 0.00 |
|  | **3** | ***R^2^*** | 0.872 | 0.962 | 0.783 | 0.778 | 0.766 | - | 0.935 | 0.738 | 0.975 | 0.910 | 0.914 | 0.737 | 0.806 | 0.662 | - | 0.664 | 0.786 | 0.705 | 0.692 |
|  |  | **h** | 1.5 | 1.5 | 1.5 | 2.0 | 1.5 | - | 3.0 | 2.0 | 1.5 | 5.0 | 3.0 | 3.0 | 8.0 | 5.0 | - | 8.0 | 3.0 | 1.5 | 5.0 |
|  |  | ***p*** | 0.07 | 0.02 | 0.12 | 0.05 | 0.65 | - | 0.00 | 0.06 | 0.02 | 0.00 | 0.00 | 0.15 | 0.00 | 0.03 | - | 0.01 | 0.02 | 0.16 | 0.04 |

**Table S4c.** *p*-values and coefficients of determinations (*R^2^*) of the release of taurine and primary amino acids by crustacean zooplankton in the coastal northern Adriatic Sea (fall). Release rates were calculated by linear regression analyses until the time point when the amino acid species or taurine did not further increase in concentration. R. indicates the number of replicates. Abbreviation for the amino acids are given in Table S3c.

| **Date** | **R.** |  | **Asp** | **Glu** | **Asn** | **Ser** | **Gln** | **His** | **Gly** | **Arg** | **Thr** | **Ala** | **Tau** | **Gaba** | **Tyr** | **Met/Val** | **Try** | **Phe** | **Ile** | **Leu** | **Lys** |
| --- | --- | --- | --- | --- | --- | --- | --- | --- | --- | --- | --- | --- | --- | --- | --- | --- | --- | --- | --- | --- | --- |
| **11/06/15** | **1** | ***R^2^*** | - | - | - | - | 0.786 | 0.941 | 0.945 | 0.723 | 0.913 | 0.737 | 0.709 | 0.853 | - | 0.825 | - | - | - | 0.656 | 0.941 |
|  |  | **h** | - | - | - | - | 8.0 | 8.0 | 5.0 | 5.0 | 5.0 | 2.0 | 5.0 | 2.0 | - | 8.0 | - | - | - | 2.0 | 2.0 |
|  |  | ***p*** | - | - | - | - | 0.02 | 0.00 | 0.00 | 0.03 | 0.00 | 0.14 | 0.04 | 0.08 | - | 0.00 | - | - | - | 0.19 | 0.03 |
|  | **2** | ***R^2^*** | - | 0.822 | - | - | 0.814 | 0.788 | 0.897 | - | 0.938 | - | 0.929 | - | 0.850 | 0.751 | - | - | 0.945 | 0.740 | 0.853 |
|  |  | **h** | - | 8.0 | - | - | 1.5 | 8.0 | 8.0 | - | 8.0 | - | 8.0 | - | 8.0 | 8.0 | - | - | 8.0 | 8.0 | 8.0 |
|  |  | ***p*** | - | 0.00 | - | - | 0.10 | 0.01 | 0.00 | - | 0.00 | - | 0.00 | - | 0.00 | 0.02 | - | - | 0.00 | 0.02 | 0.00 |
|  | **3** | ***R^2^*** | - | 0.968 | 0.706 | 0.893 | - | 0.853 | 0.996 | - | 0.894 | 0.942 | 0.878 | - | 0.944 | - | 0.934 | 0.874 | 0.703 | 0.978 | 0.862 |
|  |  | **h** | - | 3.0 | 3.0 | 5.0 | - | 3.0 | 1.5 | - | 1.5 | 1.0 | 3.0 | - | 1.5 | - | 1.0 | 5.0 | 3.0 | 1.5 | 8.0 |
|  |  | ***p*** | - | 0.00 | 0.04 | 0.00 | - | 0.01 | 0.00 | - | 0.05 | 0.16 | 0.01 | - | 0.03 | - | 0.17 | 0.00 | 0.04 | 0.01 | 0.00 |
| **11/07/15** | **1** | ***R^2^*** | - | 0.908 | 0.920 | 0.914 | 0.877 | 0.934 | 0.955 | 0.854 | 0.981 | 0.993 | 0.986 | 0.858 | 0.683 | 0.714 | 0.944 | 0.967 | 0.952 | 0.870 | 0.814 |
|  |  | **h** | - | 5.0 | 5.0 | 5.0 | 5.0 | 3.0 | 5.0 | 5.0 | 5.0 | 5.0 | 5.0 | 3.0 | 5.0 | 5.0 | 5.0 | 3.0 | 5.0 | 5.0 | 5.0 |
|  |  | ***p*** | - | 0.00 | 0.00 | 0.00 | 0.00 | 0.00 | 0.00 | 0.00 | 0.00 | 0.00 | 0.00 | 0.01 | 0.00 | 0.02 | 0.00 | 0.00 | 0.00 | 0.00 | 0.01 |
|  | **2** | ***R^2^*** | - | 0.893 | 0.919 | 0.884 | 0.873 | 0.880 | 0.987 | 0.992 | 0.968 | 0.838 | 0.977 | - | 0.961 | 0.954 | 0.961 | 0.983 | 0.998 | 0.967 | 0.975 |
|  |  | **h** | - | 5.0 | 5.0 | 2.0 | 5.0 | 5.0 | 8.0 | 1.5 | 8.0 | 5.0 | 8.0 | - | 8.0 | 8.0 | 8.0 | 8.0 | 5.0 | 8.0 | 5.0 |
|  |  | ***p*** | - | 0.00 | 0.00 | 0.06 | 0.01 | 0.01 | 0.00 | 0.06 | 0.00 | 0.01 | 0.00 | - | 0.00 | 0.00 | 0.00 | 0.00 | 0.00 | 0.00 | 0.00 |
|  | **3** | ***R^2^*** | - | 0.897 | 0.786 | 0.888 | 0.777 | 0.916 | 0.927 | 0.903 | 0.827 | 0.846 | 0.839 | 0.883 | 0.900 | 0.876 | 0.894 | 0.863 | 0.819 | 0.796 | 0.856 |
|  |  | **h** | - | 8.0 | 8.0 | 8.0 | 8.0 | 8.0 | 8.0 | 8.0 | 8.0 | 8.0 | 8.0 | 8.0 | 8.0 | 8.0 | 8.0 | 8.0 | 8.0 | 8.0 | 8.0 |
|  |  | ***p*** | - | 0.00 | 0.00 | 0.00 | 0.00 | 0.00 | 0.00 | 0.00 | 0.00 | 0.00 | 0.00 | 0.00 | 0.00 | 0.00 | 0.00 | 0.00 | 0.00 | 0.00 | 0.00 |
| **11/10/15** | **1** | ***R^2^*** | 0.834 | 0.737 | 0.644 | 0.920 | 0.823 | 0.869 | 0.936 | 0.897 | 0.941 | 0.853 | 0.959 | - | 0.989 | 0.967 | 0.574 | 0.708 | 0.674 | 0.847 | 0.913 |
|  |  | **h** | 8.0 | 8.0 | 8.0 | 8.0 | 5.0 | 8.0 | 8.0 | 8.0 | 8.0 | 8.0 | 8.0 | - | 8.0 | 8.0 | 8.0 | 8.0 | 8.0 | 8.0 | 8.0 |
|  |  | ***p*** | 0.00 | 0.01 | 0.03 | 0.00 | 0.03 | 0.00 | 0.00 | 0.00 | 0.00 | 0.00 | 0.00 | - | 0.00 | 0.00 | 0.05 | 0.01 | 0.00 | 0.00 | 0.00 |
|  | **2** | ***R^2^*** | 0.894 | 0.879 | 0.706 | 0.925 | 0.889 | 0.881 | 0.946 | 0.916 | 0.815 | 0.751 | 0.907 | 0.893 | 0.854 | 0.861 | 0.955 | 0.773 | 0.894 | 0.916 | 0.931 |
|  |  | **h** | 8.0 | 8.0 | 8.0 | 8.0 | 8.0 | 8.0 | 8.0 | 8.0 | 8.0 | 8.0 | 8.0 | 8.0 | 8.0 | 8.0 | 8.0 | 8.0 | 8.0 | 8.0 | 8.0 |
|  |  | ***p*** | 0.00 | 0.00 | 0.01 | 0.00 | 0.00 | 0.00 | 0.00 | 0.00 | 0.00 | 0.01 | 0.00 | 0.00 | 0.00 | 0.01 | 0.00 | 0.00 | 0.00 | 0.00 | 0.00 |
|  | **3** | ***R^2^*** | 0.995 | 0.983 | 0.784 | 0.822 | 0.897 | 0.646 | 0.910 | 0.813 | 0.927 | 0.865 | 0.979 | 0.787 | 0.648 | 0.683 | 0.772 | 0.931 | 0.846 | 0.935 | 0.966 |
|  |  | **h** | 5.0 | 5.0 | 5.0 | 8.0 | 5.0 | 5.0 | 5.0 | 5.0 | 5.0 | 5.0 | 5.0 | 5.0 | 5.0 | 5.0 | 8.0 | 8.0 | 5.0 | 8.0 | 5.0 |
|  |  | ***p*** | 0.00 | 0.00 | 0.01 | 0.00 | 0.00 | 0.03 | 0.00 | 0.00 | 0.00 | 0.00 | 0.00 | 0.01 | 0.03 | 0.02 | 0.00 | 0.00 | 0.00 | 0.00 | 0.00 |

**Table S4d.** *p*-values and coefficients of determinations (*R^2^*) of the release of taurine and primary amino acids by crustacean zooplankton in the coastal northern Adriatic Sea (fall). Release rates were calculated by linear regression analyses until the time point when the amino acid species or taurine did not further increase in concentration. R. indicates the number of replicates. Abbreviation for the amino acids are given in Table S3c.

| **Date** | **R.** |  | **Asp** | **Glu** | **Asn** | **Ser** | **Gln** | **His** | **Gly** | **Arg** | **Thr** | **Ala** | **Tau** | **Gaba** | **Tyr** | **Met/Val** | **Try** | **Phe** | **Ile** | **Leu** | **Lys** |
| --- | --- | --- | --- | --- | --- | --- | --- | --- | --- | --- | --- | --- | --- | --- | --- | --- | --- | --- | --- | --- | --- |
| **02/02/16** | **1** | ***R^2^*** | 0.706 | 0.939 | 0.820 | - | 0.947 | - | 0.987 | 0.788 | 0.951 | 0.992 | 0.979 | 0.874 | 0.818 | 0.810 | - | - | 0.707 | 0.659 | 0.672 |
|  |  | **h** | 8.0 | 3.0 | 3.0 | - | 3.0 | - | 3.0 | 8.0 | 5.0 | 3.0 | 3.0 | 3.0 | 8.0 | 8.0 | - | - | 8.0 | 8.0 | 8.0 |
|  |  | ***p*** | 0.01 | 0.00 | 0.00 | - | 0.00 | - | 0.00 | 0.00 | 0.00 | 0.00 | 0.00 |  | 0.00 | 0.00 | - | - | 0.01 | 0.01 | 0.01 |
|  | **2** | ***R^2^*** | 0.893 | 0.737 | 0.661 | - | - | 0.943 | 0.985 | 0.918 | 0.983 | 0.984 | 0.986 | 0.791 | - | - | - | - | - | 0.915 | 0.856 |
|  |  | **h** | 5.0 | 5.0 | 3.0 | - | - | 1.0 | 5.0 | 5.0 | 5.0 | 5.0 | 5.0 | 5.0 | - | - | - | - | - | 5.0 | 5.0 |
|  |  | ***p*** | 0.00 | 0.01 | 0.05 | - | - | 0.15 | 0.00 | 0.00 | 0.00 | 0.00 | 0.00 | 0.02 | - | - | - | - | - | 0.00 | 0.01 |
|  | **3** | ***R^2^*** | - | - | 0.702 | - | - | - | 0.969 | - | - | 0.763 | 0.900 | - | - | - | - | - | - | 0.967 | - |
|  |  | **h** | - | - | 2.0 | - | - | - | 2.0 | - | - | 5.0 | 2.0 | - | - | - | - | - | - | 2.0 | - |
|  |  | ***p*** | - | - | 0.16 | - | - | - | 0.02 | - | - | 0.02 | 0.01 | - | - | - | - | - | - | 0.12 | - |
| **02/03/16** | **1** | ***R^2^*** | - | - | 0.869 | - | 0.870 | - | 0.968 | 0.723 | 0.930 | 0.992 | 0.660 | - | - | - | - | - | - | 0.597 | 0.889 |
|  |  | **h** | - | - | 8.0 | - | 8.0 | - | 8.0 | 8.0 | 8.0 | 8.0 | 8.0 | - | - | - | - | - | - | 8.0 | 8.0 |
|  |  | ***p*** | - | - | 0.00 | - | 0.00 | - | 0.00 | 0.02 | 0.00 | 0.00 | 0.07 | - | - | - | - | - | - | 0.04 | 0.00 |
|  | **2** | ***R^2^*** | - | - | - | - | 0.680 | 0.904 | 0.852 | 0.777 | 0.888 | 0.802 | 0.869 | - | - | - | - | - | - | - | - |
|  |  | **h** | - | - | - | - | 1.5 | 1.5 | 5.0 | 3.0 | 3.0 | 2.0 | 5.0 | - | - | - | - | - | - | - | - |
|  |  | ***p*** | - | - | - | - | 0.18 | 0.05 | 0.00 | 0.05 | 0.02 | 0.10 | 0.00 | - | - | - | - | - | - | - | - |
|  | **3** | ***R^2^*** | - | - | 0.869 | - | 0.870 | - | 0.968 | 0.930 | - | 0.992 | 0.983 | - | - | - | - | - | - | - | - |
|  |  | **h** | - | - | 8.0 | - | 8.0 | - | 8.0 | 8.0 | - | 8.0 | 8.0 | - | - | - | - | - | - | - | - |
|  |  | ***p*** | - | - | 0.00 | - | 0.00 | - | 0.00 | 0.00 | - | 0.02 | 0.00 | - | - | - | - | - | - | - | - |
| **02/05/16** | **1** | ***R^2^*** | 0.976 | 0.914 | 0.927 | 0.858 | 0.972 | 0.835 | 0.944 | 0.738 | 0.973 | 0.954 | 0.879 | - | 0.935 | 0.771 | - | 0.962 | 0.721 | 0.956 | 0.985 |
|  |  | **h** | 2.0 | 2.0 | 5.0 | 8.0 | 3.0 | 8.0 | 3.0 | 3.0 | 3.0 | 3.0 | 8.0 | - | 1.5 | 1.5 | - | 2.0 | 1.5 | 8.0 | 8.0 |
|  |  | ***p*** | 0.00 | 0.01 | 0.00 | 0.00 | 0.00 | 0.00 | 0.00 | 0.03 | 0.00 | 0.00 | 0.00 | - | 0.03 | 0.12 | - | 0.02 | 0.15 | 0.00 | 0.00 |
|  | **2** | ***R^2^*** | 0.818 | - | 0.818 | 0.619 | 0.912 | - | 0.847 | 0.881 | 0.968 | 0.855 | 0.794 | 0.864 | 0.828 | 0.988 | - | - | - | 0.888 | 0.894 |
|  |  | **h** | 8.0 | - | 8.0 | 5.0 | 8.0 | - | 5.0 | 5.0 | 8.0 | 8.0 | 5.0 | 2.0 | 8.0 | 1.5 | - | - | - | 8.0 | 8.0 |
|  |  | ***p*** | 0.00 | - | 0.00 | 0.00 | 0.00 | - | 0.01 | 0.00 | 0.00 | 0.00 | 0.01 | 0.04 | 0.00 | 0.18 | - | - | - | 0.00 | 0.00 |
|  | **3** | ***R^2^*** | - | - | - | - | - | - | 0.655 | - | - | - | 0.960 | - | - | - | - | - | - | - | - |
|  |  | **h** | - | - | - | - | - | - | 8.0 | - | - | - | 5.0 | - | - | - | - | - | - | - | - |
|  |  | ***p*** | - | - | - | - | - | - | 0.01 | - | - | - | 0.01 | - | - | - | - | - | - | - | - |
| **02/09/16** | **1** | ***R^2^*** | 0.707 | 0.892 | 0.977 | 0.703 | 0.937 | 0.747 | 0.962 | 0.802 | 0.945 | 0.939 | 0.946 | - | - | - | 0.901 | 0.939 | - | 0.990 | 0.755 |
|  |  | **h** | 5.0 | 3.0 | 2.0 | 5.0 | 8.0 | 5.0 | 5.0 | 5.0 | 5.0 | 5.0 | 3.0 | - | - | - | 5.0 | 3.0 | - | 5.0 | 5.0 |
|  |  | ***p*** | 0.02 | 0.00 | 0.00 | 0.02 | 0.00 | 0.01 | 0.00 | 0.01 | 0.00 | 0.00 | 0.00 | - | - | - | 0.00 | 0.00 | - | 0.00 | 0.01 |
|  | **2** | ***R^2^*** | 0.789 | 0.793 | 0.631 | 0.654 | 0.897 | 0.571 | 0.932 | 0.868 | 0.959 | 0.950 | 0.965 | - | 0.863 | - | 0.886 | 0.924 | - | 0.965 | 0.850 |
|  |  | **h** | 8.0 | 8.0 | 8.0 | 8.0 | 8.0 | 8.0 | 8.0 | 8.0 | 8.0 | 8.0 | 8.0 | - | 8.0 | - | 8.0 | 8.0 | - | 8.0 | 8.0 |
|  |  | ***p*** | 0.00 | 0.00 | 0.02 | 0.02 | 0.00 | 0.05 | 0.00 | 0.00 | 0.00 | 0.00 | 0.00 | - | 0.00 | - | 0.00 | 0.00 | - | 0.00 | 0.00 |
|  | **3** | ***R^2^*** | - | 0.843 | 0.757 | - | 0.758 | 0.737 | 0.695 | 0.717 | 0.817 | 0.835 | 0.916 | - | 0.879 | - | - | - | - | - | 0.927 |
|  |  | **h** | - | 8.0 | 8.0 | - | 8.0 | 8.0 | 8.0 | 8.0 | 8.0 | 8.0 | 8.0 | - | 8.0 | - | - | - | - | - | 5.0 |
|  |  | ***p*** | - | 0.01 | 0.00 | - | 0.00 | 0.01 | 0.02 | 0.01 | 0.00 | 0.00 | 0.00 | - | 0.00 | - | - | - | - | - | 0.00 |

**Table S4e.** *p*-values and coefficients of determinations (*R^2^*) of the release of taurine and primary amino acids by crustacean zooplankton in the coastal northern Adriatic Sea (winter). Release rates were calculated by linear regression analyses until the time point when the amino acid species or taurine did not further increase in concentration. R. indicates the number of replicates. Abbreviation for the amino acids are given in Table S3c.

**Table S5.** Average copepod abundances in different seasons in the northern Adriatic Sea (Kamburska and Fonda-Umani 2006).

| **season** | **individual/L** |
| --- | --- |
| spring | 4.6 |
| summer | 3.0 |
| fall | 4.9 |
| winter | 5.0 |

**Table S6.** Probes and hybridization conditions used for MICRO-CARD-FISH analyses. Hyb. Temp: Hybridization temperature.

| **Probe name** | **Sequence (5’ – 3’)** | **Target group** | **Formamide [%]** | **Hyb. Temp. [°C]** | **Reference** |
| --- | --- | --- | --- | --- | --- |
| NON338 | ACTCCTACGGGAGGCAGC | negative control probe | 55 | 35 | Amann et al. (1995) |
| EUB 338 I | GCTGCCTCCCGTAGGAGT | most Bacteria | 55 | 35 | Amann et al. (1990) |
| EUB 338 II | GCAGCCACCCGTAGGTGT | Planctomycetales | 55 | 35 | Daims et al. (1999) |
| EUB 338 III | GCTGCCACCCGTAGGTGT | Verrucomicrobiales, Chloroflexi | 55 | 35 | Daims et al. (1999) |
| SAR11-152R | ATTAGCACAAGTTTCCYCGTGT | SAR 11 clade | 45 | 35 | Morris et al. (2002) |
| SAR11-441R | TACAGTCATTTTCTTCCCCGAC | SAR 11 clade | 45 | 35 | Morris et al. (2002) |
| SAR11-542R | TCCGAACTACGCTAGGTC | SAR 11 clade | 45 | 35 | Morris et al. (2002) |
| SAR11-732R | GTCAGTAATGATCCAGAAAGYTG | SAR 11 clade | 45 | 35 | Morris et al. (2002) |
| CREN 537 | TGACCATTGAGGTGCTG | Thaumarchaeota | 20 | 35 | Teira et al. (2004) |
| CREN 554 | TTAGGCCCAATAATCMTCCT | Thaumarchaeota | 20 | 35 | Woebken (2007) |
| EURY 806 | CACAGCGTTTACACCTAG | Euryarchaeota | 20 | 35 | Teira et al. (2004) |
| Ros 537 | CAACGCTAACCCCCTCC | Roseobacter | 55 | 35 | Eilers et al. (2001) |
| Ros 1029 | CTGTCACTTGGTCTCTTG | Roseobacter | 55 | 35 | Eilers et al. (2001) |
| Alt413 | TTT GCA TCC CAC TCC CAT | Alteromonas | 40 | 46 | Eilers et al. (2000) |

Amann, R. I., B. J. Binder, R. J. Olson, S. W. Chisholm, R. Devereux, and D. A. Stahl. 1990. Combination of 16S rRNA-targeted oligonucleotide probes with flow cytometry for analyzing mixed microbial populations. Appl. Environ. Microbiol. **56**: 1919–25.

Amann, R. I., W. Ludwig, and K. H. Schleifer. 1995. Phylogenetic identification and in situ detection of individual microbial cells without cultivation. Microbiol. Rev. **59**: 143–69.

Daims, H., A. Brühl, R. Amann, K.-H. Schleifer, and M. Wagner. 1999. The domain-specific probe EUB338 is insufficient for the detection of all bacteria: development and evaluation of a more comprehensive probe set. Syst. Appl. Microbiol. **22**: 434–444. doi:10.1016/S0723-2020(99)80053-8

Eilers, H., J. Pernthaler, F. O. Glöckner, and R. Amann. 2000. Culturability and in situ abundance of pelagic bacteria from the North Sea. Appl. Environ. Microbiol. **66**: 3044–3051. doi:10.1128/AEM.66.7.3044-3051.2000

Eilers, H., J. Pernthaler, J. Peplies, F. O. Glockner, G. Gerdts, and R. Amann. 2001. Isolation of novel pelagic bacteria from the German Bight and their seasonal contributions to surface picoplankton. Appl. Environ. Microbiol. **67**: 5134–5142. doi:10.1128/AEM.67.11.5134-5142.2001

Morris, R. M., M. S. Rappé, S. A. Connon, K. L. Vergin, W. A. Siebold, C. A. Carlson, and S. J. Giovannoni. 2002. SAR11 clade dominates ocean surface bacterioplankton communities. Nature **420**: 806–810. doi:10.1038/nature01240

Teira, E., T. Reinthaler, A. Pernthaler, J. Pernthaler, and G. J. Herndl. 2004. Combining catalyzed reporter deposition-fluorescence in situ hybridization and microautoradiography to detect substrate utilization by bacteria and archaea in the deep ocean. Appl. Environ. Microbiol. **70**: 4411–4414. doi:10.1128/AEM.70.7.4411-4414.2004

Woebken, D., B. M. Fuchs, M. M. M. Kuypers, and R. Amann. 2007. Potential interactions of particle-associated anammox bacteria with bacterial and archaeal partners in the Namibian upwelling system. Appl. Environ. Microbiol. **73**: 4648–4657. doi:10.1128/AEM.02774-06

**Table S7**. Inorganic and organic nutrient concentrations (mean ± SD of all 3 depths) over a seasonal cycle in the coastal northern Adriatic Sea. Abbreviations: Phosphate (PO_4_^3-^), dissolved organic phospate (DOP), nitrate (NO_3_^-^), nitrite (NO_2_^-^), ammonium (NH_4_^+^), dissolved organic nitrogen (DON), silicate (Si), total dissolved inorganic nitrogen (DIN).

| **Date** | **PO_4_^3-^** | **DOP** | **NO_3_^-^** | **NO_2_^-^** | **NH_4_^+^** | **Si** | **DON** | **DIN:PO_4_^3-^** |
| --- | --- | --- | --- | --- | --- | --- | --- | --- |
|  | **[µmol L^-1^]** | **[µmol L^-1^]** | **[µmol L^-1^]** | **[µmol L^-1^]** | **[µmol L^-1^]** | **[µmol L^-1^]** | **[µmol L^-1^]** |  |
| 04/24/15 | 0.08 ± 0.03 | 0.36 ± 0.06 | 1.8 ± 2.0 | 0.18 ± 2.04 | 0.38 ± 0.13 | 4.9 ± 1.1 | - | 31 ± 41 |
| 04/25/15 | 0.05 ± 0.02 | 0.40 ± 0.03 | 2.4 ± 1.8 | 0.08 ± 0.06 | 0.19 ± 0.03 | 5.1 ± 1.3 | - | 58 ± 46 |
| 04/27/15 | 0.06 ± 0.02 | 0.45 ± 0.13 | 1.3 ± 1.5 | 0.03 ± 0.01 | 0.23 ± 0.05 | 3.9 ± 4.1 | - | 35 ± 39 |
| 04/28/15 | 0.07 ± 0.02 | 0.29 ± 0.16 | 3.8 ± 4.2 | 0.05 ± 0.02 | 0.19 ± 0.03 | 5.4 ± 2.1 | - | 79 ± 41 |
| 04/29/15 | 0.04 ± 0.01 | 0.48 ± 0.11 | 4.1 ± 3.0 | 0.04 ± 0.01 | 0.24 ± 0.12 | 5.5 ± 1.7 | - | 109 ± 71 |
| 04/30/15 | 0.07 ± 0.01 | 0.43 ± 0.07 | 2.5 ± 1.4 | 0.04 ± 0.01 | 0.49 ± 0.39 | 6.2 ± 2.4 | - | 43 ± 23 |
| 06/24/15 | 0.05 ± 0.02 | 0.05 ± 0.02 | 9.3 ± 1.4 | 0.04 ± 0.00 | 0.75 ± 0.68 | 8.0 ± 2.3 | - | 205 ± 27 |
| 06/25/15 | 0.02 ± 0.01 | 0.04 ± 0.01 | 4.8 ± 2.3 | 0.04 ± 0.03 | 0.57 ± 0.31 | 3.9 ± 0.8 | - | 281 ± 52 |
| 06/26/15 | 0.03 ± 0.01 | 0.04 ± 0.02 | 5.8 ± 4.9 | 0.04 ± 0.02 | 1.06 ± 0.58 | 5.6 ± 2.6 | - | 317 ± 260 |
| 06/27/15 | 0.03 ± 0.02 | 0.05 ± 0.02 | 1.2 ± 0.4 | 0.03 ± 0.00 | 0.48 ± 0.18 | 3.3 ± 0.7 | - | 94 ± 58 |
| 06/29/15 | 0.03 ± 0.02 | 0.12 ± 0.04 | 7.8 ± 6.5 | 0.03 ± 0.02 | 0.64 ± 0.28 | 6.7 ± 4.1 | - | 320 ± 243 |
| 06/30/15 | 0.04 ± 0.01 | 0.13 ± 0.07 | 6.9 ± 2.6 | 0.03 ± 0.02 | 0.76 ± 0.25 | 6.0 ± 1.6 | - | 213 ± 76 |
| 07/01/15 | 0.02 ± 0.02 | 0.07 ± 0.04 | 3.8 ± 6.2 | 0.03 ± 0.02 | 0.57 ± 0.60 | 4.6 ± 3.1 | - | 128 ± 153 |
| 07/02/15 | 0.02 ± 0.00 | 0.31 ± 0.22 | 1.5 ± 1.4 | 0.04 ± 0.00 | 0.47 ± 0.23 | 3.2 ± 1.1 | - | 102 ± 77 |
| 11/02/15 | 0.04 ± 0.02 | 0.11 ± 0.02 | 2.0 ± 1.6 | 0.43 ± 0.13 | 0.45 ± 0.38 | 5.3 ± 2.3 | 5.9 ± 0.7 | 145 ± 163 |
| 11/03/15 | 0.00 ± 0.01 | 0.09 ± 0.02 | 3.7 ± 4.5 | 0.38 ± 0.03 | 0.47 ± 0.31 | 4.8 ± 3.3 | 5.8 ± 0.8 | 146 |
| 11/04/15 | 0.03 ± 0.01 | 0.11 ± 0.02 | 1.2 ± 1.0 | 0.38 ± 0.03 | 1.84 ± 2.45 | 4.7 ± 1.1 | 5.0 ± 0.1 | 137 ± 145 |
| 11/05/15 | 0.07 ± 0.04 | 0.12 ± 0.03 | 3.2 ± 3.0 | 0.27 ± 0.03 | 0.61 ± 0.39 | 4.8 ± 2.2 | 4.4 ± 1.6 | 60 ± 31 |
| 11/06/15 | 0.02 ± 0.01 | 0.10 ± 0.01 | 1.1 ± 0.8 | 0.18 ± 0.08 | 0.28 ± 0.17 | 2.4 ± 1.2 | 3.2 ± 1.3 | 105 ± 71 |
| 11/07/15 | 0.00 ± 0.01 | 0.13 ± 0.02 | 3.2 ± 2.9 | 0.23 ± 0.07 | 0.40 ± 0.15 | 3.4 ± 1.4 | 5.8 ± 1.7 | 120 |
| 11/09/15 | 0.02 ± 0.01 | 0.12 ± 0.04 | 2.0 ± 1.7 | 0.28 ± 0.04 | 1.37 ± 0.22 | 3.3 ± 1.0 | 3.7 ± 1.9 | 154 ± 24 |
| 11/10/15 | 0.02 ± 0.01 | 0.11 ± 0.03 | 3.0 ± 1.1 | 0.32 ± 0.09 | 0.16 ± 0.11 | 4.4 ± 0.0 | 4.0 ± 1.4 | 195 ± 146 |
| 11/11/15 | 0.04 ± 0.04 | 0.09 ± 0.02 | 0.8 ± 0.8 | 0.33 ± 0.06 | 0.44 ± 0.23 | 3.7 ± 1.4 | 4.0 ± 1.0 | 69 ± 52 |
| 11/12/15 | 0.01 ± 0.00 | 0.12 ± 0.04 | 0.3 ± 0.2 | 0.28 ± 0.11 | 0.21 ± 0.14 | 2.9 ± 0.9 | 3.8 ± 0.3 | 76 ± 33 |
| 02/01/16 | 0.00 ± 0.01 | 0.12 ± 0.03 | 1.0 ± 0.4 | 0.41 ± 0.08 | 0.85 ± 0.27 | 3.4 ± 1.3 | 2.6 ± 1.6 | 264 |
| 02/02/16 | 0.02 ± 0.02 | 0.12 ± 0.04 | 1.5 ± 1.1 | 0.43 ± 0.01 | 0.10 ± 0.05 | 3.4 | 2.3 ± 0.8 | 104 ± 86 |
| 02/03/16 | 0.00 ± 0.01 | 0.14 ± 0.01 | 0.06 ± 0.1 | 0.37 ± 0.07 | 0.10 ± 0.19 | 2.6 ± 0.2 | 4.5 ± 1.4 | 103 |
| 02/04/16 | 0.03 ± 0.03 | 0.06 ± 0.04 | 1.4 ± 0.4 | 0.41 ± 0.14 | 0.62 ± 0.30 | - | 2.8 ± 0.9 | 52 ± 2 |
| 02/05/16 | 0.05 ± 0.07 | 0.09 ± 0.05 | 1.7 ± 0.4 | 0.41 ± 0.02 | 0.57 ± 0.31 | 1.5 | 2.4 ± 1.8 | 202 ± 166 |
| 02/06/16 | 0.03 ± 0.03 | 0.17 ± 0.09 | .24 ± 1.5 | 0.33 ± 0.13 | 1.14 ± 0.56 | - | 1.8 ± 0.8 | 115 ± 79 |
| 02/08/16 | 0.02 ± 0.01 | 0.11 ± 0.03 | 1.6 ± 0.3 | 0.30 ± 0.13 | 0.67 ± 0.26 | 2.8 ± 1.1 | 3.2 ± 0.9 | 107 ± 127 |
| 02/09/16 | 0.06 ± 0.04 | 0.11 ± 0.08 | 1.6 ± 0.5 | 0.45 ± 0.06 | 1.06 ± 0.32 | 3.1 | 3.0 ± 0.5 | 59 ± 24 |

**Table S8a.** Concentrations of dissolved free amino acids (in nM) throughout the water column of the northern Adriatic Sea off Rovinj (spring). The sum of the dissolved free amino acids (sum DFFA), taurine/DFAA ratios and leucine/DFAA (%), the date of sampling and the sampling depth (m) are also given.

| **Date** | **Depth** | **Asp** | **Glu** | **Asn** | **Ser** | **Gln** | **His** | **Gly** | **Arg** | **Thr** | **Ala** | **Tau** | **Gaba** | **Tyr** | **Met/Val** | **Trp** | **Pha** | **Iso** | **Leu** | **Lys** | **total DFAA** | **Tau/DFAA ratio** | **Leu/DFAA [%]** |
| --- | --- | --- | --- | --- | --- | --- | --- | --- | --- | --- | --- | --- | --- | --- | --- | --- | --- | --- | --- | --- | --- | --- | --- |
| **04/22/15** | **5** | 4.2 | 5.9 | 2.8 | 8.3 | 2.4 | 1.5 | 0.9 | 0.5 | 0.5 | 0.6 | 0.9 | 0.6 | 0.3 | 0.5 | 0.5 | 0.8 | 1.1 | 0.2 | 0.3 | 32.0 | 0.028 | 0.704 |
| **04/22/15** | **10** | 8.0 | 1.6 | 10.3 | 2.3 | 1.2 | 0.1 | 0.7 | 0.3 | 0.2 | 0.5 | 0.3 | 0.3 | 2.5 | 0.4 | 1.3 | 0.9 | 1.1 | 0.2 | 0.3 | 32.1 | 0.008 | 0.591 |
| **04/22/15** | **15** | 6.0 | 2.1 | 11.8 | 2.6 | 1.3 | 0.1 | 0.9 | 0.5 | 0.5 | 0.7 | 0.4 | 0.3 | 0.3 | 0.6 | 0.4 | 0.8 | 1.1 | 0.2 | 0.2 | 30.4 | 0.015 | 0.668 |
| **04/23/15** | **5** | 15.6 | 9.8 | 14.8 | 30.8 | 3.0 | 4.0 | 17.3 | 6.9 | 2.2 | 9.8 | 3.0 | 0.7 | 0.7 | 2.9 | 0.8 | 2.3 | 2.0 | 1.5 | 0.7 | 125.7 | 0.024 | 1.190 |
| **04/23/15** | **10** | 12.8 | 7.4 | 13.8 | 21.3 | 2.8 | 2.9 | 11.0 | 5.2 | 1.9 | 6.6 | 3.0 | 0.4 | 0.3 | 3.4 | 0.9 | 0.8 | 1.3 | 1.6 | 0.7 | 95.1 | 0.031 | 1.686 |
| **04/23/15** | **15** | 8.6 | 5.6 | 12.3 | 6.9 | 1.8 | 0.5 | 5.2 | 1.5 | 1.0 | 2.0 | 1.1 | 0.9 | 0.4 | 1.0 | 1.3 | 1.4 | 0.4 | 0.6 | 0.6 | 51.9 | 0.021 | 1.175 |
| **04/24/15** | **5** | 8.1 | 10.1 | 12.3 | 6.5 | 3.5 | 2.6 | 3.7 | 1.8 | 1.8 | 2.4 | 4.3 | 1.1 | 0.7 | 1.2 | 0.9 | 1.3 | 0.0 | 0.8 | 1.1 | 59.9 | 0.071 | 1.277 |
| **04/24/15** | **10** | 6.1 | 3.3 | 10.5 | 3.2 | 1.6 | 0.5 | 1.5 | 0.9 | 0.8 | 1.2 | 1.2 | 0.3 | 0.5 | 0.8 | 0.6 | 0.7 | 2.4 | 0.9 | 0.5 | 36.4 | 0.034 | 2.471 |
| **04/24/15** | **15** | 9.0 | 7.1 | 12.9 | 5.3 | 1.8 | 1.3 | 2.8 | 1.3 | 1.2 | 1.7 | 2.2 | 0.9 | 0.6 | 0.8 | 0.9 | 1.2 | 1.0 | 0.5 | 0.7 | 51.0 | 0.043 | 1.068 |
| **04/25/15** | **5** | 9.9 | 6.3 | 13.3 | 6.6 | 1.2 | 1.0 | 3.6 | 1.7 | 1.2 | 2.0 | 1.6 | 0.6 | 0.7 | 0.9 | 0.4 | 1.2 | 1.4 | 0.8 | 1.0 | 53.7 | 0.030 | 1.428 |
| **04/25/15** | **10** | 4.8 | 8.1 | 14.6 | 3.6 | 1.7 | 1.6 | 1.6 | 0.7 | 0.9 | 1.2 | 2.2 | 1.1 | 0.5 | 0.9 | 0.3 | 1.1 | 1.0 | 0.4 | 0.4 | 44.6 | 0.050 | 0.814 |
| **04/25/15** | **15** | 7.8 | 4.0 | 12.0 | 3.0 | 0.7 | 0.2 | 2.5 | 1.1 | 1.4 | 1.4 | 0.8 | 0.0 | 0.2 | 1.1 | 0.0 | 1.3 | 2.2 | 0.2 | 0.3 | 39.5 | 0.020 | 0.549 |
| **04/27/15** | **5** | 4.6 | 1.0 | 11.4 | 2.4 | 1.1 | 0.1 | 0.9 | 0.2 | 0.2 | 0.5 | 0.4 | 0.7 | 0.3 | 0.6 | 0.5 | 0.8 | 0.8 | 0.2 | 0.1 | 26.3 | 0.015 | 0.620 |
| **04/27/15** | **10** | 7.5 | 3.5 | 11.7 | 3.1 | 0.6 | 0.2 | 1.5 | 0.6 | 0.8 | 0.8 | 0.4 | 0.7 | 0.3 | 0.8 | 1.4 | 1.1 | 2.7 | 0.2 | 0.3 | 37.8 | 0.010 | 0.503 |
| **04/27/15** | **15** | 6.3 | 1.8 | 12.6 | 2.6 | 0.8 | 0.1 | 1.9 | 0.4 | 0.6 | 0.6 | 0.5 | 0.7 | 0.3 | 0.3 | 0.4 | 0.8 | 1.6 | 0.1 | 0.3 | 32.2 | 0.014 | 0.422 |
| **04/28/15** | **5** | 10.5 | 6.2 | 12.9 | 13.8 | 1.2 | 0.7 | 7.7 | 3.1 | 1.2 | 4.2 | 1.6 | 0.4 | 1.1 | 1.4 | 0.4 | 1.4 | 1.2 | 1.6 | 1.7 | 70.7 | 0.022 | 2.277 |
| **04/28/15** | **10** | 7.8 | 5.8 | 11.7 | 3.8 | 1.2 | 1.1 | 1.9 | 0.6 | 0.8 | 1.2 | 1.6 | 0.7 | 0.4 | 0.6 | 1.0 | 1.1 | 0.9 | 0.5 | 0.8 | 41.9 | 0.038 | 1.145 |
| **04/28/15** | **15** | 8.4 | 3.8 | 13.7 | 7.7 | 2.0 | 0.8 | 3.8 | 1.7 | 1.1 | 2.2 | 1.3 | 0.9 | 0.6 | 0.8 | 0.9 | 1.6 | 1.2 | 0.6 | 0.2 | 51.9 | 0.025 | 1.160 |
| **04/29/15** | **5** | 14.7 | 10.0 | 15.7 | 26.2 | 3.0 | 2.7 | 13.6 | 6.4 | 10.3 | 7.6 | 2.6 | 1.0 | 1.5 | 2.0 | 0.0 | 1.7 | 2.4 | 2.1 | 1.3 | 122.2 | 0.021 | 1.731 |
| **04/29/15** | **10** | 18.2 | 9.3 | 15.3 | 41.2 | 1.9 | 4.7 | 24.1 | 9.5 | 2.0 | 11.4 | 2.1 | 0.9 | 3.0 | 0.4 | 0.0 | 0.6 | 1.3 | 2.6 | 1.4 | 147.9 | 0.014 | 1.780 |
| **04/29/15** | **15** | 7.5 | 5.1 | 12.8 | 4.1 | 1.7 | 1.2 | 2.3 | 1.0 | 1.2 | 1.5 | 2.0 | 1.0 | 0.4 | 0.6 | 0.7 | 1.0 | 1.3 | 0.4 | 0.2 | 44.1 | 0.045 | 0.862 |

Abbreviations: St, station; R, replicates; Asp, Aspartic acid; Glu, Glutamic acid; Asn, Asparagine; Ser, Serine; Gln, Glutamine; His, Histine; Gly, Glycine; Arg, Arginine; Thr, Threonine; Ala, Alanine; Tau, Taurine; GABA, gamma aminobutyric acid; Tyr, Tyrosine; Met, Methionine; Val, Valine; Trp, Tryptophan; Phe, Phenylalanine; Ile, Isoleucine; Leu, Leucine; Lys, Lysine.

**Table S8b.** Concentrations of dissolved free amino acids (in nM) throughout the water column of the northern Adriatic Sea off Rovinj, (summer). The sum of the dissolved free amino acids (sum DFFA), taurine/DFAA ratios and leucine/DFAA (%), the date of sampling and the sampling depth (m) are also given.

| **Date** | **Depth** | **Asp** | **Glu** | **Asn** | **Ser** | **Gln** | **His** | **Gly** | **Arg** | **Thr** | **Ala** | **Tau** | **Gaba** | **Tyr** | **Met/Val** | **Trp** | **Pha** | **Iso** | **Leu** | **Lys** | **total DFAA** | **Tau/DFAA ratio** | **Leu/DFAA [%]** |
| --- | --- | --- | --- | --- | --- | --- | --- | --- | --- | --- | --- | --- | --- | --- | --- | --- | --- | --- | --- | --- | --- | --- | --- |
| **06/24/15** | **5** | 13.2 | 14.3 | 17.3 | 4.7 | 6.4 | 1.3 | 6.9 | 2.2 | 1.8 | 17.0 | 4.5 | 0.4 | 0.2 | 5.3 | 1.2 | 0.5 | 0.8 | 10.2 | 2.0 | 105.6 | 0.043 | 9.677 |
| **06/24/15** | **10** | 16.8 | 21.6 | 20.4 | 15.3 | 13.2 | 3.7 | 11.5 | 4.7 | 5.7 | 11.0 | 3.1 | 0.5 | 1.5 | 0.7 | 1.1 | 2.3 | 1.4 | 4.0 | 1.8 | 137.3 | 0.023 | 2.901 |
| **06/24/15** | **15** | 5.4 | 4.9 | 13.6 | 2.8 | 2.7 | 2.0 | 1.8 | 0.5 | 1.0 | 1.6 | 2.7 | 0.7 | 0.5 | 0.5 | 0.5 | 0.4 | 1.5 | 3.6 | 1.8 | 45.6 | 0.059 | 7.881 |
| **06/25/15** | **5** | 6.0 | 16.0 | 16.0 | 2.9 | 11.4 | 1.0 | 3.0 | 1.1 | 1.7 | 26.6 | 3.5 | 0.5 | 0.6 | 0.4 | 0.5 | 0.8 | 1.5 | 1.3 | 3.2 | 94.6 | 0.037 | 1.424 |
| **06/25/15** | **10** | 8.1 | 4.4 | 12.5 | 2.0 | 1.4 | 1.2 | 1.4 | 0.4 | 1.0 | 0.8 | 1.5 | 0.4 | 0.5 | 0.4 | 0.9 | 0.6 | 1.2 | 0.8 | 2.0 | 40.0 | 0.037 | 2.089 |
| **06/25/15** | **15** | 4.6 | 4.6 | 11.7 | 1.2 | 2.7 | 0.6 | 1.4 | 1.1 | 1.5 | 4.1 | 1.3 | 0.4 | 0.4 | 0.4 | 0.8 | 0.1 | 1.9 | 1.4 | 3.4 | 42.4 | 0.031 | 3.379 |
| **06/26/15** | **5** | 8.0 | 16.4 | 15.4 | 3.9 | 7.3 | 1.2 | 2.4 | 1.4 | 6.7 | 4.2 | 4.3 | 0.5 | 1.3 | 0.6 | 0.9 | 1.4 | 1.9 | 2.3 | 3.5 | 79.1 | 0.055 | 2.859 |
| **06/26/15** | **10** | 11.2 | 7.0 | 11.9 | 3.8 | 2.0 | 1.0 | 4.1 | 1.2 | 2.2 | 2.6 | 7.5 | 0.6 | 1.3 | 0.7 | 0.8 | 0.8 | 3.2 | 0.5 | 1.9 | 56.7 | 0.132 | 0.799 |
| **06/26/15** | **15** | 9.8 | 5.6 | 14.0 | 1.5 | 1.1 | 0.8 | 7.4 | 1.4 | 1.0 | 3.4 | 3.3 | 0.5 | 0.8 | 0.8 | 1.0 | 0.4 | 1.1 | 0.5 | 1.3 | 52.4 | 0.062 | 0.921 |
| **06/27/15** | **5** | 9.9 | 5.7 | 16.6 | 2.9 | 1.6 | 0.6 | 3.9 | 1.1 | 1.4 | 2.6 | 2.0 | 0.5 | 0.6 | 1.1 | 1.0 | 0.4 | 2.1 | 0.8 | 1.3 | 54.2 | 0.037 | 1.514 |
| **06/27/15** | **10** | 11.3 | 5.0 | 14.9 | 0.9 | 1.2 | 1.6 | 5.7 | 1.3 | 1.3 | 4.7 | 2.9 | 0.5 | 0.7 | 1.3 | 0.5 | 1.0 | 0.3 | 1.0 | 1.5 | 54.8 | 0.052 | 1.870 |
| **06/27/15** | **15** | 7.2 | 3.3 | 14.8 | 3.0 | 1.0 | 0.9 | 4.5 | 1.2 | 1.2 | 4.4 | 2.5 | 0.5 | 0.7 | 0.8 | 0.9 | 0.3 | 2.8 | 0.6 | 0.6 | 48.9 | 0.050 | 1.314 |
| **06/29/15** | **5** | 7.1 | 4.8 | 19.3 | 5.7 | 2.9 | 0.8 | 4.8 | 1.5 | 2.1 | 5.3 | 2.7 | 0.5 | 1.0 | 1.2 | 1.8 | 0.9 | 0.6 | 1.0 | 0.7 | 62.1 | 0.043 | 1.656 |
| **06/29/15** | **10** | 9.4 | 6.8 | 18.0 | 2.4 | 1.8 | 1.8 | 3.4 | 0.6 | 1.6 | 6.1 | 3.3 | 0.6 | 0.7 | 0.9 | 1.2 | 0.9 | 0.4 | 0.7 | 1.2 | 58.5 | 0.056 | 1.126 |
| **06/29/15** | **15** | 9.2 | 7.7 | 15.1 | 3.1 | 2.8 | 2.4 | 4.2 | 0.7 | 1.3 | 7.9 | 4.7 | 0.7 | 0.7 | 1.1 | 1.4 | 0.9 | 0.3 | 0.9 | 1.2 | 61.4 | 0.077 | 1.387 |
| **06/30/15** | **5** | 9.8 | 6.5 | 16.8 | 3.4 | 1.6 | 1.0 | 4.4 | 1.4 | 2.0 | 16.5 | 3.1 | 0.6 | 0.7 | 1.0 | 0.9 | 1.0 | 0.3 | 0.8 | 1.1 | 69.9 | 0.044 | 1.179 |
| **06/30/15** | **10** | 10.3 | 5.5 | 16.8 | 5.5 | 2.2 | 2.4 | 7.3 | 1.8 | 1.2 | 2.5 | 2.7 | 0.5 | 0.9 | 0.9 | 1.1 | 0.8 | 0.3 | 1.0 | 1.3 | 62.4 | 0.044 | 1.652 |
| **06/30/15** | **15** | 5.3 | 3.2 | 14.6 | 2.8 | 1.8 | 2.2 | 3.4 | 0.6 | 1.5 | 2.8 | 5.6 | 0.5 | 0.8 | 0.9 | 1.0 | 0.5 | 1.0 | 0.7 | 1.1 | 45.0 | 0.125 | 1.615 |
| **07/02/15** | **5** | 9.9 | 6.3 | 17.1 | 3.2 | 1.6 | 0.6 | 3.5 | 0.8 | 1.3 | 18.6 | 2.4 | 0.5 | 0.4 | 1.2 | 1.1 | 0.7 | 0.5 | 0.8 | 1.3 | 69.4 | 0.035 | 1.195 |
| **07/02/15** | **10** | 11.3 | 5.0 | 14.9 | 0.9 | 1.2 | 1.6 | 5.7 | 1.3 | 1.3 | 4.7 | 2.9 | 0.5 | 0.7 | 1.3 | 0.5 | 1.0 | 0.3 | 1.0 | 1.5 | 54.8 | 0.052 | 1.870 |
| **07/02/15** | **15** | 8.3 | 4.1 | 14.2 | 7.6 | 1.7 | 2.0 | 13.7 | 2.7 | 1.3 | 4.0 | 2.2 | 0.2 | 1.0 | 1.1 | 0.0 | 0.8 | 0.5 | 1.5 | 1.9 | 66.7 | 0.033 | 2.310 |

Abbreviations: St, station; R, replicates; Asp, Aspartic acid; Glu, Glutamic acid; Asn, Asparagine; Ser, Serine; Gln, Glutamine; His, Histine; Gly, Glycine; Arg, Arginine; Thr, Threonine; Ala, Alanine; Tau, Taurine; GABA, gamma aminobutyric acid; Tyr, Tyrosine; Met, Methionine; Val, Valine; Trp, Tryptophan; Phe, Phenylalanine; Ile, Isoleucine; Leu, Leucine; Lys, Lysine.

**Table S8c.** Concentrations of dissolved free amino acids (in nM) throughout the water column of the northern Adriatic Sea off Rovinj, (fall). The sum of the dissolved free amino acids (sum DFFA), taurine/DFAA ratios and leucine/DFAA (%), the date of sampling and the sampling depth (m) are also given.

| **Date** | **Depth** | **Asp** | **Glu** | **Asn** | **Ser** | **Gln** | **His** | **Gly** | **Arg** | **Thr** | **Ala** | **Tau** | **Gaba** | **Tyr** | **Met/Val** | **Trp** | **Pha** | **Iso** | **Leu** | **Lys** | **total DFAA** | **Tau/DFAA ratio** | **Leu/DFAA [%]** |
| --- | --- | --- | --- | --- | --- | --- | --- | --- | --- | --- | --- | --- | --- | --- | --- | --- | --- | --- | --- | --- | --- | --- | --- |
| **11/02/15** | **5** | 7.5 | 14.6 | 19.6 | 5.2 | 12.1 | 5.9 | 17.2 | 2.5 | 2.6 | 6.5 | 21.8 | 0.5 | 1.2 | 4.5 | 2.6 | 0.6 | 0.8 | 0.9 | 1.6 | 106.6 | 0.204 | 0.870 |
| **11/02/15** | **10** | 21.1 | 17.5 | 30.5 | 5.8 | 12.5 | 2.0 | 14.2 | 4.1 | 4.6 | 6.8 | 16.5 | 2.6 | 2.0 | 3.9 | 6.1 | 1.0 | 1.2 | 0.6 | 0.9 | 137.3 | 0.120 | 0.454 |
| **11/02/15** | **15** | 23.5 | 26.2 | 26.5 | 6.6 | 8.4 | 7.8 | 23.2 | 3.9 | 8.4 | 17.6 | 0.8 | 2.3 | 2.2 | 4.2 | 1.0 | 1.1 | 1.4 | 0.6 | 1.1 | 166.0 | 0.005 | 0.349 |
| **11/03/15** | **5** | 13.8 | 17.2 | 16.5 | 8.3 | 11.8 | 7.0 | 28.9 | 3.9 | 2.3 | 9.8 | 14.6 | 0.3 | 1.4 | 3.3 | 1.8 | 0.6 | 0.9 | 0.6 | 1.6 | 130.1 | 0.113 | 0.423 |
| **11/03/15** | **10** | 18.5 | 9.0 | 22.8 | 46.3 | 6.0 | 8.3 | 35.7 | 12.1 | 2.0 | 21.0 | 13.9 | 0.5 | 2.7 | 6.2 | 1.8 | 0.5 | 3.8 | 6.5 | 5.4 | 209.0 | 0.067 | 3.096 |
| **11/03/15** | **15** | 10.5 | 5.9 | 2.1 | 10.3 | 3.4 | 2.2 | 8.2 | 1.8 | 0.8 | 3.2 | 2.6 | 0.4 | 0.7 | 3.0 | 1.5 | 0.4 | 0.3 | 0.6 | 1.2 | 56.5 | 0.045 | 1.137 |
| **11/04/15** | **5** | 14.9 | 8.8 | 16.0 | 19.2 | 2.8 | 1.5 | 25.8 | 6.1 | 1.8 | 9.7 | 7.7 | 0.4 | 1.4 | 6.2 | 2.0 | 0.8 | 1.4 | 1.7 | 2.1 | 122.5 | 0.063 | 1.392 |
| **11/04/15** | **10** | 12.8 | 27.9 | 12.1 | 4.3 | 4.8 | 2.1 | 18.7 | 4.5 | 3.5 | 11.7 | 10.5 | 0.7 | 0.4 | 3.9 | 1.9 | 1.1 | 1.1 | 2.2 | 1.0 | 114.9 | 0.092 | 1.927 |
| **11/04/15** | **15** | 10.3 | 6.1 | 15.1 | 10.9 | 2.7 | 2.2 | 17.6 | 4.4 | 1.7 | 7.5 | 5.1 | 0.4 | 0.4 | 5.6 | 1.7 | 0.5 | 1.2 | 1.6 | 2.0 | 91.9 | 0.056 | 1.719 |
| **11/05/15** | **5** | 12.4 | 12.7 | 17.8 | 19.3 | 10.3 | 5.0 | 20.9 | 6.3 | 2.1 | 11.0 | 11.0 | 0.3 | 1.5 | 8.5 | 2.1 | 0.9 | 2.0 | 1.7 | 0.8 | 135.6 | 0.081 | 1.240 |
| **11/05/15** | **10** | 36.7 | 14.5 | 17.2 | 106.9 | 3.1 | 15.4 | 78.8 | 22.2 | 4.1 | 35.3 | 7.7 | 0.6 | 6.1 | 14.4 | 3.8 | 3.9 | 6.2 | 7.1 | 2.6 | 379.0 | 0.020 | 1.883 |
| **11/05/15** | **15** | 6.4 | 2.6 | 14.1 | 1.8 | 0.9 | 1.1 | 6.2 | 1.2 | 0.7 | 2.0 | 2.1 | 0.4 | 0.5 | 5.4 | 2.1 | 0.2 | 0.4 | 0.5 | 0.4 | 47.2 | 0.044 | 1.011 |
| **11/06/15** | **5** | 15.5 | 9.2 | 12.8 | 29.2 | 0.9 | 4.5 | 25.3 | 8.7 | 2.9 | 12.7 | 4.9 | 0.2 | 2.1 | 9.9 | 3.1 | 1.7 | 3.6 | 3.2 | 3.1 | 148.9 | 0.033 | 2.165 |
| **11/06/15** | **10** | 10.2 | 4.4 | 14.5 | 5.2 | 1.1 | 1.7 | 7.1 | 1.8 | 0.7 | 2.9 | 1.6 | 0.2 | 0.6 | 3.5 | 1.8 | 0.2 | 0.7 | 0.6 | 1.0 | 58.2 | 0.027 | 0.954 |
| **11/06/15** | **15** | 8.4 | 7.3 | 13.4 | 4.4 | 2.2 | 1.7 | 9.7 | 1.7 | 1.0 | 3.1 | 6.1 | 0.2 | 0.5 | 1.7 | 1.4 | 0.3 | 0.5 | 0.6 | 1.0 | 59.2 | 0.103 | 1.027 |
| **11/07/15** | **5** | 7.9 | 4.2 | 14.8 | 6.1 | 1.5 | 1.5 | 9.0 | 8.5 | 1.2 | 2.8 | 3.4 | 0.2 | 0.6 | 1.1 | 1.1 | 0.3 | 0.4 | 0.7 | 1.3 | 63.2 | 0.054 | 1.047 |
| **11/07/15** | **10** | 10.0 | 5.7 | 14.7 | 13.5 | 1.5 | 2.4 | 14.6 | 3.9 | 1.1 | 5.9 | 3.3 | 0.2 | 1.0 | 3.5 | 1.4 | 0.6 | 0.7 | 1.9 | 1.5 | 84.0 | 0.040 | 2.236 |
| **11/07/15** | **15** | 14.1 | 7.9 | 16.4 | 28.3 | 2.4 | 5.0 | 22.9 | 8.3 | 2.2 | 11.0 | 4.5 | 0.2 | 1.7 | 3.9 | 1.7 | 1.1 | 1.6 | 2.2 | 4.6 | 135.6 | 0.033 | 1.616 |
| **11/09/15** | **5** | 10.9 | 8.8 | 15.1 | 8.8 | 0.7 | 1.9 | 11.0 | 3.0 | 0.9 | 7.0 | 2.2 | 0.4 | 1.9 | 4.9 | 1.7 | 0.4 | 0.6 | 0.9 | 1.0 | 79.9 | 0.027 | 1.068 |
| **11/09/15** | **10** | 9.9 | 6.5 | 15.6 | 6.6 | 1.7 | 1.9 | 9.8 | 1.8 | 0.5 | 3.6 | 8.3 | 0.3 | 0.6 | 2.7 | 0.9 | 0.3 | 0.5 | 0.5 | 1.2 | 65.0 | 0.128 | 0.805 |
| **11/09/15** | **15** | 5.4 | 4.1 | 17.9 | 6.7 | 0.7 | 1.1 | 7.7 | 1.9 | 0.6 | 3.1 | 5.8 | 0.3 | 1.1 | 1.0 | 0.9 | 0.7 | 0.7 | 0.5 | 0.9 | 55.2 | 0.106 | 0.840 |
| **11/10/15** | **5** | 11.5 | 7.2 | 17.3 | 16.1 | 6.0 | 5.5 | 14.8 | 6.7 | 5.5 | 9.9 | 4.5 | 2.6 | 1.3 | 8.3 | 2.0 | 1.0 | 1.1 | 1.6 | 3.2 | 121.7 | 0.037 | 1.348 |
| **11/10/15** | **10** | 11.9 | 9.5 | 15.4 | 8.6 | 3.9 | 4.0 | 16.6 | 3.2 | 1.3 | 5.5 | 6.2 | 0.1 | 0.9 | 5.1 | 2.2 | 0.3 | 1.0 | 1.0 | 1.0 | 91.6 | 0.068 | 1.106 |
| **11/10/15** | **15** | 20.3 | 16.7 | 16.1 | 38.5 | 3.2 | 6.3 | 33.2 | 14.3 | 3.0 | 20.7 | 7.0 | 0.5 | 2.1 | 10.3 | 2.0 | 1.6 | 5.7 | 3.8 | 3.6 | 201.9 | 0.035 | 1.880 |
| **11/11/15** | **5** | 14.9 | 8.7 | 48.3 | 1.0 | 0.5 | 4.2 | 23.6 | 7.3 | 1.3 | 10.2 | 4.4 | 0.2 | 1.3 | 4.9 | 1.6 | 0.9 | 1.7 | 0.8 | 2.3 | 133.6 | 0.033 | 0.572 |
| **11/11/15** | **10** | 14.3 | 7.2 | 15.8 | 19.9 | 1.8 | 3.6 | 20.9 | 6.4 | 1.1 | 9.5 | 3.6 | 0.5 | 1.3 | 3.7 | 1.4 | 1.4 | 1.8 | 0.6 | 2.2 | 113.3 | 0.032 | 0.567 |
| **11/11/15** | **15** | 11.6 | 6.5 | 17.1 | 8.2 | 1.5 | 2.7 | 13.5 | 3.2 | 1.0 | 5.1 | 3.5 | 0.5 | 0.7 | 3.8 | 1.6 | 0.4 | 1.2 | 0.9 | 1.2 | 80.8 | 0.043 | 1.144 |

Abbreviations: St, station; R, replicates; Asp, Aspartic acid; Glu, Glutamic acid; Asn, Asparagine; Ser, Serine; Gln, Glutamine; His, Histine; Gly, Glycine; Arg, Arginine; Thr, Threonine; Ala, Alanine; Tau, Taurine; GABA, gamma aminobutyric acid; Tyr, Tyrosine; Met, Methionine; Val, Valine; Trp, Tryptophan; Phe, Phenylalanine; Ile, Isoleucine; Leu, Leucine; Lys, Lysine.

**Table S8d.** Concentrations of dissolved free amino acids (in nM) throughout the water column of the northern Adriatic Sea off Rovinj, (fall). The sum of the dissolved free amino acids (sum DFFA), taurine/DFAA ratios and leucine/DFAA (%), the date of sampling and the sampling depth (m) are also given.

| **Date** | **Depth** | **Asp** | **Glu** | **Asn** | **Ser** | **Gln** | **His** | **Gly** | **Arg** | **Thr** | **Ala** | **Tau** | **Gaba** | **Tyr** | **Met/Val** | **Trp** | **Pha** | **Iso** | **Leu** | **Lys** | **total DFAA** | **Tau/DFAA ratio** | **Leu/DFAA [%]** |
| --- | --- | --- | --- | --- | --- | --- | --- | --- | --- | --- | --- | --- | --- | --- | --- | --- | --- | --- | --- | --- | --- | --- | --- |
| **02/01/16** | **5** | 8.3 | 5.7 | 11.1 | 2.1 | 0.6 | 1.0 | 11.3 | 1.3 | 0.6 | 7.0 | 3.3 | 0.5 | 0.4 | 2.9 | 0.7 | 0.3 | 0.5 | 0.4 | 0.4 | 54.9 | 0.060 | 0.731 |
| **02/01/16** | **10** | 10.0 | 3.7 | 14.0 | 18.5 | 1.4 | 3.7 | 14.4 | 4.8 | 0.5 | 17.4 | 3.0 | 0.4 | 1.5 | 2.8 | 1.0 | 0.9 | 1.0 | 1.3 | 0.6 | 97.9 | 0.031 | 1.340 |
| **02/01/16** | **15** | 7.0 | 4.1 | 11.5 | 1.1 | 0.6 | 0.2 | 2.8 | 0.3 | 0.2 | 1.5 | 1.7 | 0.1 | 0.3 | 2.0 | 0.4 | 0.1 | 0.1 | 0.2 | 1.2 | 34.0 | 0.049 | 0.526 |
| **02/02/16** | **5** | 7.3 | 1.9 | 12.0 | 3.4 | 0.7 | 0.9 | 3.6 | 0.7 | 0.2 | 1.7 | 0.5 | 0.5 | 0.4 | 1.9 | 1.3 | 0.4 | 0.3 | 0.3 | 0.8 | 38.1 | 0.013 | 0.747 |
| **02/02/16** | **10** | 7.3 | 3.2 | 11.8 | 10.7 | 1.1 | 1.9 | 9.5 | 3.0 | 0.5 | 4.7 | 1.1 | 0.6 | 0.5 | 3.8 | 0.6 | 0.4 | 0.8 | 0.3 | 0.6 | 61.2 | 0.018 | 0.421 |
| **02/02/16** | **15** | 9.7 | 5.6 | 12.4 | 16.7 | 2.8 | 3.5 | 17.5 | 7.2 | 0.9 | 6.1 | 1.0 | 0.5 | 1.6 | 3.0 | 1.3 | 0.8 | 1.0 | 1.3 | 0.5 | 92.4 | 0.010 | 1.396 |
| **02/03/16** | **5** | 9.9 | 4.6 | 12.6 | 10.6 | 1.1 | 2.5 | 14.9 | 3.2 | 0.8 | 4.9 | 2.2 | 0.2 | 0.4 | 3.2 | 0.8 | 0.5 | 0.7 | 0.8 | 0.9 | 72.9 | 0.030 | 1.116 |
| **02/03/16** | **10** | 7.1 | 3.4 | 10.8 | 3.2 | 1.7 | 0.9 | 6.4 | 1.7 | 0.3 | 2.6 | 0.5 | 0.3 | 0.4 | 2.6 | 0.5 | 0.3 | 0.7 | 0.5 | 1.0 | 44.3 | 0.012 | 1.034 |
| **02/03/16** | **15** | 6.5 | 4.2 | 10.8 | 2.3 | 1.0 | 0.7 | 5.7 | 0.9 | 0.3 | 2.5 | 1.1 | 0.2 | 0.4 | 1.4 | 0.4 | 0.2 | 0.3 | 0.5 | 0.8 | 38.9 | 0.028 | 1.212 |
| **02/04/16** | **5** | 7.7 | 4.5 | 12.1 | 2.5 | 1.0 | 0.1 | 5.0 | 1.0 | 0.3 | 2.3 | 0.9 | 0.2 | 0.4 | 2.2 | 0.5 | 0.2 | 0.4 | 0.4 | 0.6 | 41.3 | 0.022 | 0.872 |
| **02/04/16** | **10** | 6.9 | 2.6 | 11.4 | 8.0 | 0.4 | 1.0 | 7.2 | 2.3 | 0.6 | 3.6 | 0.7 | 0.6 | 0.4 | 2.8 | 0.5 | 0.4 | 0.9 | 0.5 | 0.9 | 51.0 | 0.013 | 1.006 |
| **02/04/16** | **15** | 6.8 | 4.6 | 11.2 | 3.0 | 0.8 | 0.5 | 5.9 | 1.1 | 0.4 | 3.2 | 1.5 | 0.1 | 0.4 | 2.2 | 0.5 | 0.2 | 0.8 | 0.4 | 0.6 | 42.6 | 0.036 | 0.904 |
| **02/05/16** | **5** | 12.3 | 5.7 | 9.2 | 29.4 | 1.3 | 6.9 | 24.5 | 8.7 | 1.2 | 12.0 | 3.1 | 0.5 | 1.9 | 5.1 | 1.1 | 1.4 | 0.9 | 2.7 | 1.1 | 125.9 | 0.024 | 2.140 |
| **02/05/16** | **10** | 7.0 | 3.3 | 11.6 | 7.9 | 1.2 | 3.2 | 8.3 | 2.0 | 0.7 | 4.3 | 3.9 | 0.7 | 0.4 | 3.3 | 0.6 | 0.5 | 0.2 | 0.7 | 0.3 | 56.0 | 0.070 | 1.303 |
| **02/05/16** | **15** | 7.6 | 4.0 | 12.0 | 6.4 | 0.9 | 1.9 | 6.2 | 1.5 | 0.5 | 3.3 | 2.5 | 0.4 | 0.3 | 1.4 | 0.4 | 0.4 | 0.6 | 0.8 | 0.9 | 49.2 | 0.050 | 1.549 |
| **02/06/16** | **5** | 4.7 | 1.7 | 9.0 | 5.3 | 0.7 | 1.4 | 5.1 | 1.3 | 0.3 | 2.3 | 1.2 | 0.3 | 0.3 | 4.8 | 0.3 | 0.2 | 0.6 | 0.4 | 0.7 | 39.6 | 0.030 | 1.082 |
| **02/06/16** | **10** | 11.8 | 4.3 | 11.7 | 26.1 | 1.4 | 4.4 | 19.6 | 7.0 | 1.0 | 9.5 | 2.2 | 0.2 | 0.5 | 3.4 | 0.7 | 0.9 | 1.5 | 1.8 | 0.5 | 106.5 | 0.021 | 1.736 |
| **02/06/16** | **15** | 7.4 | 2.9 | 7.5 | 12.6 | 0.7 | 2.7 | 10.6 | 3.7 | 0.5 | 5.1 | 0.6 | 0.2 | 0.8 | 2.7 | 0.7 | 0.5 | 0.9 | 1.1 | 1.3 | 62.0 | 0.010 | 1.727 |
| **02/08/16** | **5** | 7.2 | 4.3 | 10.0 | 1.6 | 0.4 | 0.3 | 7.1 | 0.4 | 0.3 | 1.5 | 1.2 | 0.2 | 0.3 | 2.2 | 1.0 | 0.5 | 0.1 | 0.1 | 0.3 | 37.9 | 0.030 | 0.372 |
| **02/08/16** | **10** | 5.8 | 2.7 | 11.0 | 0.7 | 0.3 | 0.1 | 3.2 | 0.5 | 0.2 | 1.2 | 0.5 | 0.3 | 0.3 | 1.5 | 1.3 | 0.5 | 0.1 | 0.2 | 0.3 | 30.3 | 0.015 | 0.581 |
| **02/08/16** | **15** | 5.7 | 3.0 | 10.6 | 0.6 | 0.6 | 0.5 | 2.9 | 0.4 | 0.3 | 0.9 | 1.1 | 0.3 | 0.4 | 2.2 | 0.6 | 0.1 | 0.1 | 0.1 | 0.3 | 29.5 | 0.038 | 0.368 |
| **02/09/16** | **5** | 4.7 | 1.8 | 8.9 | 2.7 | 0.6 | 1.2 | 3.9 | 0.6 | 0.4 | 1.5 | 1.5 | 0.1 | 0.3 | 2.7 | 0.5 | 0.2 | 0.3 | 0.4 | 0.3 | 31.2 | 0.049 | 1.131 |
| **02/09/16** | **10** | 5.6 | 2.4 | 8.2 | 4.4 | 0.6 | 1.3 | 5.0 | 1.1 | 0.3 | 2.0 | 1.1 | 0.2 | 0.3 | 2.7 | 0.4 | 0.4 | 0.4 | 0.5 | 0.9 | 36.9 | 0.031 | 1.425 |
| **02/09/16** | **15** | 15.2 | 4.1 | 13.7 | 34.5 | 1.2 | 5.0 | 26.2 | 9.5 | 1.2 | 12.8 | 1.7 | 0.4 | 2.1 | 4.8 | 0.7 | 1.5 | 0.1 | 2.8 | 1.3 | 137.2 | 0.012 | 2.051 |

Abbreviations: St, station; R, replicates; Asp, Aspartic acid; Glu, Glutamic acid; Asn, Asparagine; Ser, Serine; Gln, Glutamine; His, Histine; Gly, Glycine; Arg, Arginine; Thr, Threonine; Ala, Alanine; Tau, Taurine; GABA, gamma aminobutyric acid; Tyr, Tyrosine; Met, Methionine; Val, Valine; Trp, Tryptophan; Phe, Phenylalanine; Ile, Isoleucine; Leu, Leucine; Lys, Lysine.

**Table S9.** Taurine turnover rates (based on total uptake and assimilation rates) and leucine turnover rates (d^-1^) in the northern Adriatic Sea (mean ± SD of all 3 depths). Taurine and leucine turnover rates based on zooplankton release rates and abundances (retrieved from literature) are also indicated (mean ± SD of the replicates).

| **Date** | **taurine turnover** | | | **leucine turnover** | |
| --- | --- | --- | --- | --- | --- |
|  | based on total uptake | based on assimilation | based on zooplankton | based on incorporation | based on zooplankton |
| 04/21/15 | - | - | 1.6 | - | - |
| 04/22/15 | 7.3 ± 6.5 | - | 0.3 ± 0.3 | 3.3 ± 0.2 | - |
| 04/23/15 | 2.1 ± 1.7 | 0.8 ± 0.5 | - | 0.8 ± 0.7 | - |
| 04/24/15 | 2.5 ± 1.7 | 0.5 ± 0.3 | - | 1.6 ± 0.1 | - |
| 04/25/15 | 3.5 ± 3.1 | 1.3 ± 1.1 | - | 3.1 ± 2.6 | - |
| 04/27/15 | 11.2 ± 1.5 | 2.8 ± 0.6 | 3.4 ± 1.9 | 6.9 ± 0.1 | 0.8 |
| 04/28/15 | 4.4 ± 0.8 | 1.2 ± 0.1 | 0.5 ± 0.6 | 2.3 ± 1.1 | - |
| 04/29/15 | 2.6 ± 0.2 | 0.7 ± 0.1 | - | 1.6 ± 1.0 | - |
| 06/24/15 | 0.9 ± 0.5 | 0.5 ± 0.1 | 0.5 ± 0.1 | 3.9 ± 1.6 | 0.8 |
| 06/25/15 | 2.4 ± 0.8 | 1.2 ± 0.1 | 0.7 ± 0.4 | 2.8 ± 1.0 | 0.7 |
| 06/26/15 | 1.2 ± 0.2 | 0.3 ± 0.2 | - | 1.7 ± 0.1 | - |
| 06/27/15 | 1.4 ± 0.6 | 0.6 ± 0.4 | 0.8 | 2.2 ± 1.8 | 1.4 |
| 06/29/15 | 1.1 ± 0.2 | 0.5 ± 0.1 | - | 3.6 ± 1.3 | - |
| 06/30/15 | 0.8 ± 0.5 | 0.4 ± 0.2 | - | 2.8 ± 0.6 | - |
| 07/01/15 | 1.7 ± 0.5 | 1.0 ± 0.1 | - | 3.2 ± 1.5 | - |
| 11/02/15 | 1.8 ± 2.3 | 1.4 ± 2.0 | - | 5.9 ± 1.5 | - |
| 11/03/15 | 0.8 ± 0.7 | 0.7 ± 0.6 | 0.3 ± 0.3 | 2.9 ± 2.1 | 4.1 ± 5.8 |
| 11/04/15 | 0.6 ± 0.1 | 0.4 ± 0.1 | 2.8 ± 0.9 | 1.1 ± 0.1 | 3.2 ± 1.8 |
| 11/05/15 | 1.0 ± 1.1 | 0.7 ± 0.6 | 1.6 ± 0.3 | 3.0 ± 3.5 | 5.8 ± 2.7 |
| 11/06/15 | 1.5 ± 1.1 | 0.9 ± 0.7 | 1.5 ± 0.5 | 3.8 ± 2.8 | 2.2 ± 1.8 |
| 11/07/15 | 1.0 ± 0.1 | 0.6 ± 0.1 | 1.4 ± 0.4 | 1.9 ± 1.2 | 2.4 ± 1.6 |
| 11/09/15 | 0.9 ± 0.6 | 0.6 ± 0.4 | - | 4.3 ± 1.2 | - |
| 11/10/15 | 0.7 ± 0.2 | 0.5 ± 0.1 | 2.3 ± 1.3 | 1.8 ± 1.1 | 2.2 ± 0.9 |
| 11/11/15 | 1.2 ± 0.1 | 0.7 ± 0.1 | - | 4.0 ± 1.0 | - |
| 02/01/16 | 0.7 ± 0.3 | 0.4 ± 0.2 | - | 1.3 ± 1.1 | - |
| 02/02/16 | 1.8 ± 0.8 | 1.2 ± 0.5 | 5.2 ± 0.9 | 1.0 ± 0.6 | 1.1 ± 0.1 |
| 02/03/16 | 1.8 ± 1.0 | 1.3 ± 1.0 | 2.6 ± 1.8 | 0.7 ± 0.3 | 1.2 |
| 02/04/16 | 2.1 ± 0.7 | 1.3 ± 0.5 |  | 1.0 ± 0.2 | - |
| 02/05/16 | 1.4 ± 0.4 | 0.6 ± 0.1 | 7.4 ± 7.5 | 0.9 ± 0.5 | 4.0 ± 3.3 |
| 02/06/16 | 4.6 ± 2.4 | 2.3 ± 1.4 | - | 1.1 ± 0.9 | - |
| 02/08/16 | 3.3 ± 2.2 | 1.7 ± 0.9 | - | 4.1 ± 1.1 | - |
| 02/09/16 | 1.8 ± 0.5 | 1.1 ± 0.4 | 4.9 ± 0.7 | 0.9 ± 0.7 | 2.6 |

**Table S10.** Percentage of taurine- and leucine assimilating cells of the total prokaryotic abundance during different seasons in the coastal northern Adriatic Sea assessed via MICRO-CARD-FISH.

| **Date** | **Depth** | **Taurine assimilating cells** | **Leucine assimilating cells** |
| --- | --- | --- | --- |
|  | **[m]** | **[%]** | **[%]** |
| **04/29/15** | **5** | 63 | 58 |
|  | **10** | 62 | 58 |
|  | **15** | 58 | 55 |
| **06/30/15** | **5** | 21 | 69 |
|  | **10** | 27 | 64 |
|  | **15** | 30 | 59 |
| **11/11/15** | **5** | 41 | 55 |
|  | **10** | 43 | 54 |
|  | **15** | 45 | 54 |
| **02/08/16** | **5** | 65 | 67 |
|  | **10** | 59 | 68 |
|  | **15** | 64 | 72 |

**Table S11.** Contribution of the bacterial and archaeal taxa to the prokaryotic community (% of total prokaryotic abundance), and percentage of taurine and leucine assimilating cells from different phylogenetic taxa to the total prokaryotic abundance (% active taxa of the total prokaryotic abundance) through the water column of the coastal northern Adriatic Sea as determined by MICRO-CARD-FISH (mean ± SD of all 3 depths).

|  |  |  | **Taurine** | **Leucine** |
| --- | --- | --- | --- | --- |
| **Date** | **Taxa** | **% taxa of total prokaryotic abundance** | **% active taxa of the total prokaryotic abundance** | **% active taxa of the total prokaryotic abundance** |
| **04/29/15** | **Bacteria** | 66 ± 3 | 43 ± 2 | 43 ± 4 |
|  | **SAR11** | 37 ± 13 | 28 ± 8 | 28 ± 9 |
|  | ***Roseobacter*** | 8 ± 1 | 6 ± 1 | 7 ± 2 |
|  | ***Alteromonas*** | 5 ± 1 | 4 ± 1 | 4 ± 2 |
|  | **Thaumarcheaota** | 6 ± 1 | 3 ± 1 | 4 ± 2 |
|  | **Euryarcheoata** | 12 ± 3 | 10 ± 4 | 9 ± 3 |
| **06/30/15** | **Bacteria** | 53 ± 6 | 20 ± 3 | 37 ± 3 |
|  | **SAR11** | 32 ± 7 | 12 ± 2 | 24 ± 6 |
|  | ***Roseobacter*** | 3 ± 2 | 1 ± 0 | 2 ± 1 |
|  | ***Alteromonas*** | 5 ± 1 | 2 ± 1 | 4 ± 1 |
|  | **Thaumarcheaota** | 5 ± 2 | 2 ± 1 | 4 ± 1 |
|  | **Euryarcheoata** | 14 ± 7 | 7 ± 3 | 11 ± 6 |
| **11/11/15** | **Bacteria** | 57 ± 1 | 26 ± 5 | 39 ± 1 |
|  | **SAR11** | 20 ± 1 | 14 ± 1 | 17 ± 2 |
|  | ***Roseobacter*** | 6 ± 2 | 5 ± 2 | 4 ± 1 |
|  | ***Alteromonas*** | 10 ± 0 | 6 ± 1 | 8 ± 0 |
|  | **Thaumarcheaota** | 8 ± 0 | 4 ± 1 | 4 ± 1 |
|  | **Euryarcheoata** | 11 ± 4 | 4 ± 1 | 7 ± 3 |
| **02/08/16** | **Bacteria** | 65 ± 2 | 47 ± 3 | 49 ± 5 |
|  | **SAR11** | 46 ± 5 | 40 ± 2 | 38 ± 6 |
|  | ***Roseobacter*** | 3 ± 0 | 2 ± 1 | 2 ± 0 |
|  | ***Alteromonas*** | 7 ± 2 | 5 ± 2 | 4 ± 1 |
|  | **Thaumarcheaota** | 6 ± 1 | 5 ± 1 | 5 ± 1 |
|  | **Euryarcheoata** | 7 ± 1 | 5 ± 1 | 6 ± 1 |

**Table S12.** Contribution of taurine-carbon, -nitrogen, -sulfur to the bulk biomass production measured via leucine incorporation (mean ± SD of all 3 depths).

| **Date** | **Taurine-C contribution** | **Taurine-N contribution** | **Taurine-S contribution** |
| --- | --- | --- | --- |
|  | **[in %]** | **[in %]** | **[in %]** |
| 04/23/15 | 2.0 ± 0.2 | 4.0 ± 0.4 | 26.3 ± 2.8 |
| 04/24/15 | 1.5 ± 0.7 | 3.1 ± 1.3 | 20.1 ± 8.5 |
| 04/25/15 | 1.9 ± 0.4 | 3.7 ± 0.8 | 24.1 ± 5.5 |
| 04/27/15 | 1.6 ± 0.6 | 3.3 ± 1.1 | 21.3 ± 7.2 |
| 04/28/15 | 1.7 ± 0.2 | 3.3 ± 0.4 | 21.7 ± 2.4 |
| 04/29/15 | 1.5 ± 0.2 | 3.1 ± 0.4 | 19.9 ± 2.8 |
| 06/24/15 | 0.1 ± 0.1 | 0.3 ± 0.1 | 1.7 ± 0.8 |
| 06/25/15 | 1.1 ± 0.2 | 2.2 ± 0.3 | 14.4 ± 2.0 |
| 06/26/15 | 1.9 ± 1.2 | 3.8 ± 2.3 | 24.9 ± 15.1 |
| 06/27/15 | 1.3 ± 0.1 | 2.6 ± 0.3 | 17.1 ± 1.8 |
| 06/29/15 | 0.9 ± 0.0 | 1.8 ± 0.1 | 11.7 ± 0.4 |
| 06/30/15 | 0.9 ± 0.2 | 1.8 ± 0.4 | 11.9 ± 2.6 |
| 07/01/15 | 1.2 ± 0.2 | 2.4 ± 0.3 | 15.5 ± 2.1 |
| 11/02/15 | 1.8 ± 0.5 | 3.5 ± 1.1 | 23.1 ± 7.1 |
| 11/03/15 | 2.4 ± 0.3 | 4.8 ± 0.6 | 31.3 ± 4.2 |
| 11/04/15 | 2.3 ± 0.1 | 4.7 ± 0.3 | 30.4 ± 1.7 |
| 11/05/15 | 1.4 ± 0.1 | 2.9 ± 0.2 | 18.8 ± 1.1 |
| 11/06/15 | 1.4 ± 0.4 | 2.8 ± 0.7 | 18.0 ± 4.8 |
| 11/07/15 | 1.6 ± 0.4 | 3.2 ± 0.9 | 20.5 ± 5.6 |
| 11/09/15 | 1.4 ± 0.2 | 2.8 ± 0.4 | 18.4 ± 2.4 |
| 11/10/15 | 1.5 ± 0.2 | 3.1 ± 0.3 | 20.1 ± 2.1 |
| 11/11/15 | 1.5 ± 0.2 | 2.9 ± 0.5 | 18.9 ± 3.2 |
| 02/01/16 | 3.7 ± 0.3 | 7.4 ± 0.5 | 48.4 ± 3.5 |
| 02/02/16 | 3.8 ± 0.4 | 7.6 ± 0.8 | 49.3 ± 5.4 |
| 02/03/16 | 4.7 ± 0.3 | 9.5 ± 0.7 | 61.6 ± 4.3 |
| 02/04/16 | 4.6 ± 0.3 | 9.1 ± 0.3 | 59.4 ± 2.0 |
| 02/05/16 | 3.0 ± 0.0 | 6.0 ± 0.1 | 38.8 ± 0.4 |
| 02/06/16 | 4.5 ± 0.4 | 9.0 ± 0.7 | 58.7 ± 4.7 |
| 02/08/16 | 3.6 ± 0.2 | 7.2 ± 0.5 | 47.0 ± 3.1 |
| 02/09/16 | 4.4 ± 1.1 | 8.9 ± 2.3 | 57.7 ± 14.9 |

**Table S13.** Contribution of taurine-nitrogen and -sulfur to the single cell biomass production (based on leucine assimilation) of the specific prokaryotic taxa (mean ± SD of all 3 depths), assessed by quantitative MICRO-CARD-FISH in the northern Adriatic Sea over a seasonal cycle.

| **Taxa** | **Taurine-N contribution (in %)** | | | | **Taurine-S contribution (in %)** | | | |
| --- | --- | --- | --- | --- | --- | --- | --- | --- |
|  | **Spring** | **Summer** | **Fall** | **Winter** | **Spring** | **Summer** | **Fall** | **Winter** |
| **Bacteria** | 4.9 ± 1.4 | 4.1 ± 0.3 | 5.4 ± 0.4 | 4.8 ± 0.3 | 31.8 ± 8.9 | 26.9 ± 1.6 | 34.9 ± 2.3 | 31.1 ± 1.9 |
| **SAR11** | 5.5 ± 1.2 | 4.8 ± 0.7 | 4.7 ± 0.4 | 4.1 ± 0.7 | 35.6 ± 8.1 | 31.2 ± 4.8 | 30.3 ± 2.9 | 26.6 ± 4.5 |
| ***Roseobacter*** | 4.8 ± 1.1 | 5.5 ± 1.4 | 5.7 ± 2.4 | 6.1 ± 1.7 | 31.4 ± 7.3 | 35.7 ± 9.3 | 36.9 ± 15.6 | 40.4 ± 10.8 |
| ***Alteromonas*** | 6.9 ± 2.6 | 5.4 ± 0.6 | 6.5 ± 0.8 | 7.5 ± 2.9 | 44.7 ± 17.1 | 35.4 ± 4.0 | 42.2 ± 5.1 | 48.6 ± 18.9 |
| **Thaumarchaeota** | 5.4 ± 1.9 | 5.5 ± 1.5 | 8.0 ± 0.4 | 10.2 ± 4.0 | 34.9 ± 12.2 | 35.8 ± 9.7 | 52.3 ± 2.9 | 66.1 ± 25.9 |
| **Euryarchaeota** | 6.9 ± 1.8 | 5.9 ± 0.2 | 7.1 ± 1.6 | 7.6 ± 1.9 | 45.0 ± 11.6 | 38.2 ± 1.6 | 46.5 ± 10.5 | 49.3 ± 12.3 |
